# Synthesis, Molecular Docking Analysis and *in Vitro* Biological Evaluation of Some New Heterocyclic Scaffolds-Based Indole Moiety as Possible Antimicrobial Agents

**DOI:** 10.3389/fmolb.2021.775013

**Published:** 2022-01-17

**Authors:** Entesar A. Hassan, Ihsan A. Shehadi, Awatef M. Elmaghraby, Hadir M. Mostafa, Salem E. Zayed, Aboubakr H. Abdelmonsef

**Affiliations:** ^1^ Chemistry Department, Faculty of Science, South Valley University, Qena, Egypt; ^2^ Department of chemistry, Pure and Applied Chemistry Research Group, College of Sciences, University of Sharjah, Sharjah, UAE

**Keywords:** oxoketene gem-dithiol, 1, 2-dithiol-3-thione, hydroquinoline, dibromochromenol, thiopyran, molecular docking, *in vitro* evaluation

## Abstract

In the present study, a general approach for the synthesis of 1-(1*H*-indol-3-yl)-3,3-dimercaptoprop-2-en-1-one **(1)** and 5-(1*H*-indol-3-yl)-3*H*-1,2-dithiole-3-thione **(2)** was performed. They are currently used as efficient precursors for the synthesis of some new compounds bearing five- and/or six-membered heterocyclic moieties, e.g., chromenol **(3, 4)**, 3,4-dihydroquinoline **(7, 8)** and thiopyran **(10, 12)**-based indole core. In addition, molecular docking studies were achieved, which showed that all the newly synthesized compounds are interacting with the active site region of the target enzymes, the targets UDP-N-acetylmuramatel-alanine ligase (MurC), and human lanosterol14α-demethylase, through hydrogen bonds and pi-stacked interactions. Among these docked ligand molecules, the compound (**9)** was found to have the minimum binding energy (−11.5 and −8.5 Kcal/mol) as compared to the standard drug ampicillin (−8.0 and −8.1 Kcal/mol) against the target enzymes UDP-N-acetylmuramatel-alanine ligase (MurC), and Human lanosterol14α-demethylase, respectively. Subsequently, all new synthesized analogues were screened for their antibacterial activities against Gram-positive (*Bacillus subtilis*), and Gram-negative bacteria (*Escherichia coli*), as well as for antifungal activities against *Candida albicans* and *Aspergillus flavus*. The obtained data suggest that the compounds exhibited good to excellent activity against bacterial and fungi strains. The compound (E)-2-(6-(*1H*-indole-3-carbonyl)-5-thioxotetrahydrothieno [3,2-b]furan-2(3H)-ylidene)-3-(*1H*-indol-3-yl)-3-oxopropanedithioic acid **(9**) showed a high binding affinity as well as an excellent biological activity. Therefore, it could serve as the lead for further optimization and to arrive at potential antimicrobial agent.

## Introduction

Infective diseases have become one of the most serious threats to global health due to appearance and expansion of microorganisms’ resistance to a majority of therapeutics currently utilized for their treatment (Abo-Bakr et al., 2021). Therefore, the discovery and synthesis of new types of antibacterial and antifungal agents is a very important demand.

**FIGURE 1:**
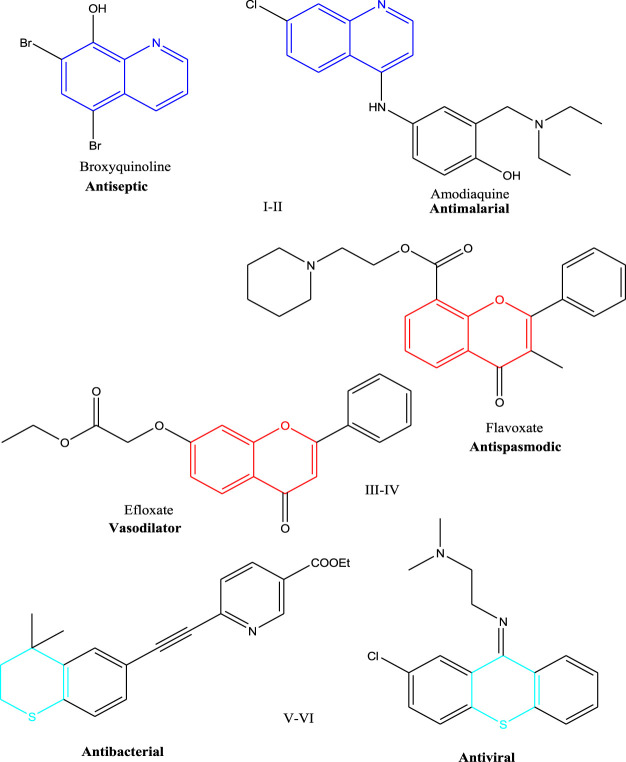
Examples of biologically active molecules containing quinoline, chromene, and thiopyran moieties (**I-VI**).

Heterocyclic cores are versatile privileged scaffolds present in many biologically active molecules (Welsch et al., 2010) and pharmaceuticals (Balaban et al., 2004; Kowada et al., 2010). The heterocyclic-based compounds with N, O, or S atoms such as quinolines **I-II**, chromenes **III-IV**, and thiopyrans **V-VI** are biologically useful molecules in drug discovery and development (Welsch et al., 2010), as shown in Figure 1.

In addition, 1,2-dithiol derivatives show many significant pharmacological activities (He et al., 2004). Among these, the ketene *gem*-dithiols act as 1,3-electrophilic synthons besides the activity role as building blocks in the synthesis of cyclic molecules. Oxoketene *gem*-dithiols are important multifunctional substrates for the synthesis of heterocyclic compounds, in addition to the nematicidal and bactericide properties (Zayed, 1996, 2007; Liang et al., 2014; Hassan et al., 2020).

Furthermore, 3*H*-1,2-dithiole-3-thione derivatives have diverse biological activities such as chemotherapeutic, antioxidant, and radio protective properties (Smith et al., 2001; Begleiter and Lange, 2002), besides being treated as chemo preventive, and sialagogue agents in various biomodels (Munday et al., 2006). In addition, the 1,2-dithiole-3-thione derivatives were found to possess anti-HIV activities (Munday et al., 2010) and cytoprotective effects (Zhu et al., 2009).

On the other hand, the indole moiety is found in various bioactive heterocycles of alkaloids as well as agrochemicals and pharmaceuticals (Baumann et al., 2011). The molecular structures of well-known drugs such as fluvastatin and rizatriptan are based on indole framework (Shafakat Ali et al., 2013). Indoles with five- and/or six-membered heterocyclic systems in the 3-position have gained considerable interest due to their significant anti-cancer activity (Yuan et al., 2009; Verma et al., 2012; Chadha and Silakari, 2017; Bhale et al., 2019).

Inspired by these facts, the aim of our research is the synthesis of a series of compounds containing five- and/or six-membered heterocycles-based indole moiety and evaluation their biological potentials through *in silico* and *in vitro* techniques. Herein, we reported the synthesis of a dataset of heterocycles such as chromenol, quinoline and pyran derivatives, compounds with promising antimicrobial properties, integrated with indole nucleus based on ketene *gem*-dithiol (**1**) and/or 1,2-dithiole-3-thione (**2)**. In an effort to elucidate the plausible mechanism by which these compounds could be used as antimicrobial drug candidates, the *in silico* molecular docking approach was carried out for all synthesized molecules against the active site regions of UDP-N-acetylmuramatel-alanine ligase (MurC), and human lanosterol14α-demethylase enzymes. In addition, they were *in vitro* tested for their antimicrobial activity against various strains of bacteria and fungi. Further, theoretical ADMET predictions were also calculated for all compounds.

## Results and Discussion

### Chemistry

In the present work, 1-(1*H*-indol-3-yl)-3,3-dimercaptoprop-2-en-1-one **(1)** was easily prepared through the reaction of 3-acetylindole with CS_2_ in the presence of K. *tert*.butoxide. Its mass spectrum showed a molecular ion peak *m/z* at 234.89, while IR spectrum revealed the presence of carbonyl group at 1,665 cm^−1^.^1^H-NMR spectrum showed signals assigned for thiol group protons, aromatic protons, and C (*sp* ^*2*^)-H proton.

**SCHEME 1:**
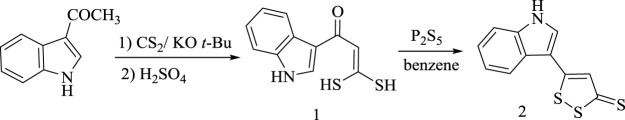
Synthesis of compounds 1 and 2.

Sulfurization of compound **(1)** with P_2_S_5_ in dry benzene afforded 1,2-dithiole-3-thione derivative **(2)** in a moderate yield, as represented in Scheme 1. The full analyses for adduct **(2)** were found to be supported the postulated structure. The ^1^H-NMR spectrum represented olefinic C (*sp* ^*2*^)-H proton signal at δ 5.93 ppm and signals assigned for the aromatic protons. In addition, its IR spectrum revealed the disappearance of the carbonyl group band and the appearance of a peak characteristic for C=S at 1,143 cm^−1^. The mass spectrum showed a molecular ion peak *m/z* at 249.10 which is in agreement with the suggested structure.

**SCHEME 2:**
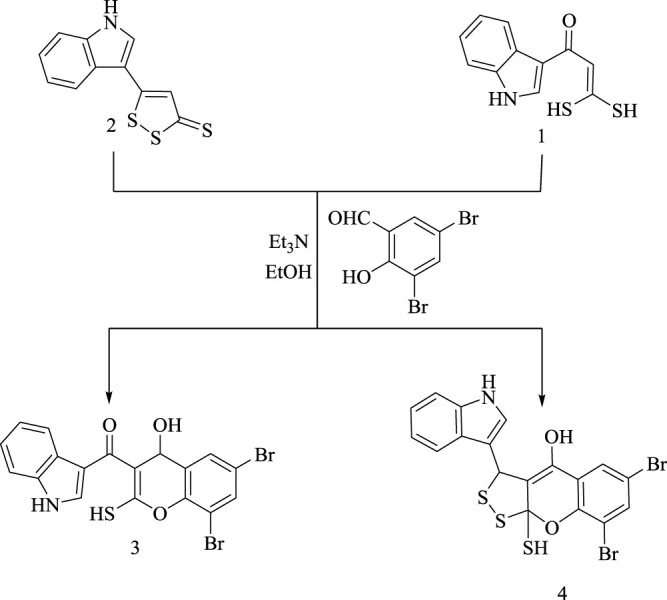
Synthetic methods for compounds 3 and 4.

In continuation of our efforts on the development of synthetic methodologies for heterocycles using the starting materials (**1**), and (**2**), herein, we reported the synthesis of a new dibromomercaptochromenol derivative (**3**) *via* reaction of α-oxoketene gem-dithiol (**1**) with 3,5-dibromosalicyldehyde. Furthermore, on reacting 1,2-dithiole-3-thione **(2)** with 3,5-dibromosalicyldehyde in the presence of Et_3_N as a base catalyst in ethanol afford dibromodithiolochromenol derivative **(4)**, as shown in Scheme 2.

The structures of the products were elucidated using spectral and elemental analyses. For compound (**3**), the ^1^H-NMR spectrum showed aliphatic C (*sp* ^*3*^)-H proton and aromatic protons, in addition to ^13^C-NMR spectrum which showed signals assigned for aromatic carbon atoms, carbonyl carbon, olefinic carbons, and C (*sp* ^*3*^)-H carbon. For compound **(4)**, the mass spectrum exhibited a molecular ion peak *m/z* at 528.74. The ^1^H-NMR spectrum showed peaks assigned for aromatic protons, thiol group, C (*sp* ^*3*^)-H proton, and hydroxyl group proton, while ^13^C-NMR spectrum showed signals assigned for aromatic carbon atoms, C (*sp* ^*3*^)-H carbons, and olefinic carbon atoms. Further, IR spectrum revealed the presence of the hydroxyl group at 3,412 cm^−1^.

**SCHEME 3:**
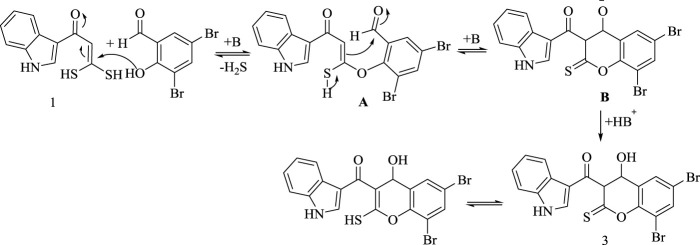
Synthetic pathway for compound 3.

The suggested synthetic route for the formation of **(3)** is assumed to proceed *via* displacement of one sulfohydryl functional group by hydroxyl group in 3,5-dibromo- salicyldehyde under base catalyst followed by nucleophilic addition of C (*sp* ^*2*^)-H to aldehyde group, as represented in Scheme 3.

**SCHEME 4:**
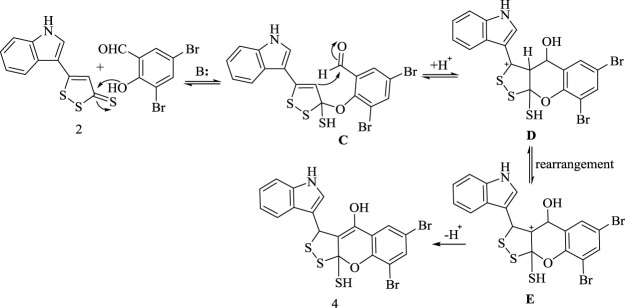
Synthetic route of compound 4.

On the other hand, the suggested synthetic route for formation of **(4)** is assumed to proceed *via* nucleophilic addition of hydroxyl group in 3,5-dibromosalicyldehyde under the effect of the base to C3 bearing thione group in **(2)** prior to Michael addition of C4 in species **(C)** to aldehyde group followed by intramolecular rearrangement, as represented in Scheme 4.

**SCHEME 5:**
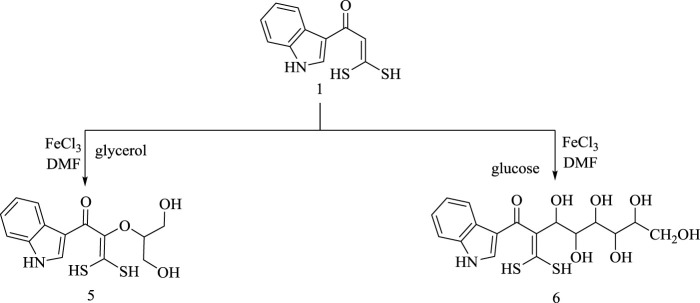
Synthesis of compounds 5 and 6.

Oxoketene *gem*-dithiol **(1)** was allowed to react with glycerol and/or glucose in the presence of ferric chloride as an oxidant reagent (Monrad and Madsen, 2011) and DMF yielded dihydroxypropan-2-yl)oxy)-1-(1*H*-indol-3-yl)-3,3-dimercapto-prop-2-en-1-one **(5)**, and 2-(dimercaptomethylene)-3,4,5,6,7,8-hexahydroxy-1-(1*H*-indol-3-yl)octan-1-one **(6)**, respectively, as shown in Scheme 5.

The structures of compounds **(5)** and **(6)** were confirmed by elemental and spectral data. The ^1^H-NMR spectrum of product (**5**) revealed the presence of C (*sp* ^*3*^)-H protons, thiol group proton, aromatic protons, and disappearance of olefinic C (*sp* ^*2*^)-H signals which illustrate the formation of the desired product. ^13^C-NMR showed peaks assigned for aromatic carbon atoms, carbonyl carbon, C (*sp* ^*3*^)-H carbons, and olefinic carbon atoms. The IR spectrum showed absorption bands at 1,708 and 3,360 cm^−1^ corresponding to carbonyl and hydroxyl groups, respectively. The mass spectrum indicated a molecular ion *m/z* at 326.68.

The proposed reaction pathway for formation of product **(6)** is assumed to be performed *via* nucleophilic addition of α-C (*sp* ^*2*^)-H carbon in **(1)** to aldehyde group in glucose. The ^1^H-NMR spectrum exhibited the disappearance of olefinic C (*sp* ^*2*^)-H peak and showed peaks assigned for aromatic protons, hydroxyl group, NH group, aliphatic (C-H) protons, and thiol group only. ^13^C-NMR spectrum showed peaks assigned for aromatic carbon atoms, carbonyl carbon, aliphatic carbons, and olefinic carbon atoms. IR spectrum revealed the presence of the carbonyl group and the hydroxyl group at 1,659 and 3,395 cm^−1^, respectively. Mass spectrum showed a molecular ion peak *m/z* at 415.14.

**SCHEME 6:**
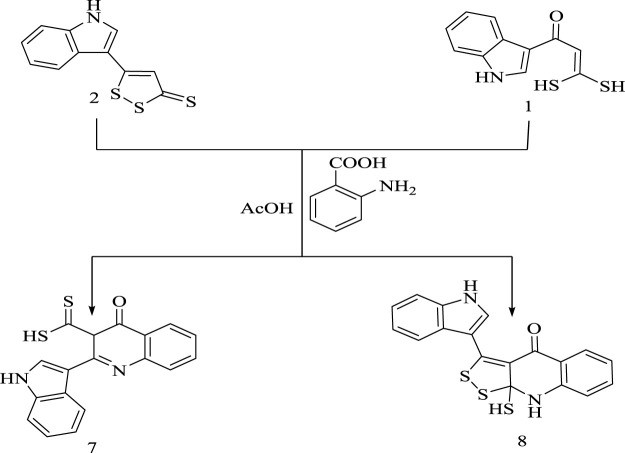
Synthesis of compounds 7 and 8.

At the same time, the behavior of oxoketene *gem*-dithiol **(1)** as a dienophile and 1,2-dithiol-3-thione **(2)** towards anthranilic acid in the presence of acetic acid as a Lewis acid was also studied. These reactions resulted in the formation of dihydroquinoline derivatives **(7)** and **(8)**, respectively, as declared in Scheme 6.

^1^H-NMR of **(7**) showed signals assigned for aromatic protons, C (*sp* ^*3*^)-H proton, NH group proton, and SH group proton. ^13^C-NMR showed peaks assigned for aromatic carbons, carbonyl carbon, thione group carbon, and one aliphatic carbon only. The mass spectrum showed a molecular ion peak *m/z* at 336.03. IR spectrum revealed the presence of carbonyl and thione groups at 1,677 and 1,142 cm^−1^, respectively.

On the other hand, mass spectrum of **(8)** exhibited a molecular ion peak *m/z* at 367.33, while ^1^H-NMR spectrum showed peaks assigned for the aromatic protons, SH proton, and 2NH protons. ^13^C-NMR spectrum represented signals assigned for aromatic carbon atoms, aliphatic carbons, olefinic carbon, and carbonyl carbon receptively. IR spectrum revealed the presence of carbonyl group at 1,698 cm^−1^.

**SCHEME 7:**
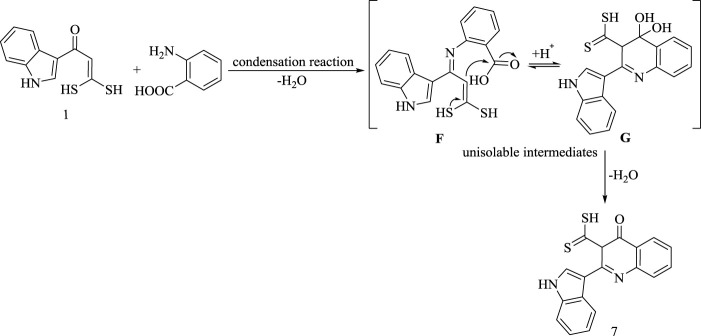
Synthesis of compound 7.

The formation of hydroquinoline derivative **(7)** is assumed to proceed *via* condensation reaction followed by nucleophilic addition of C (*sp* ^*2*^)-H carbon to carbonyl group and finally elimination of water, as represented in Scheme 7.

**SCHEME 8:**
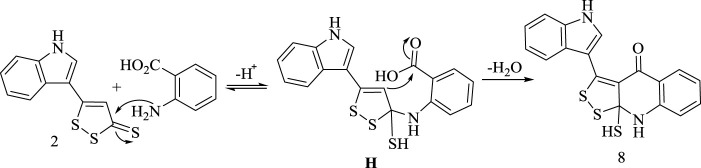
Synthetic route for formation of compound 8.

Furthermore, formation of **(8)** is assumed to proceed through acid catalyst activating nucleophilic addition of amino group in anthranilic acid to C3 bearing thione group in **(2)** followed by dehydration and ring closure, as shown in Scheme 8.

**SCHEME 9:**
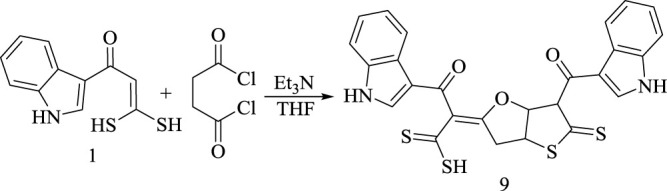
Synthesis of compounds 9.

On treating **(1)** with succinyl dichloride in 2:1 M ratio, in boiling THF and Et_3_N as catalyst, the product 2-(6-(1*H*-indole-3-carbonyl)-5-thioxotetrahydrothieno [3,2-*b*]furan-2(3*H*)-ylidene)-3-(1*H*-indol-3-yl)-3-oxo-propanedithioic acid **(9)** was obtained, as represented in Scheme 9. Its structure was proved according to the obtained spectral measurements. ^1^H-NMR spectrum exhibited no olefinic C (*sp* ^*2*^)-H proton signal and showed peaks assigned for aromatic protons, NH group, aliphatic C (*sp* ^*3*^)-H protons, and thiol group. ^13^C-NMR showed signals assigned for aromatic carbon atoms, carbonyl carbons, thione carbons, C (*sp* ^*3*^)-H carbons, and olefinic carbon atoms. IR spectrum revealed the presence of two carbonyl groups at 1,714 and 1,616 cm^−1^. Mass spectrum showed a molecular ion peak *m/z* at 533.72.

**SCHEME 10:**
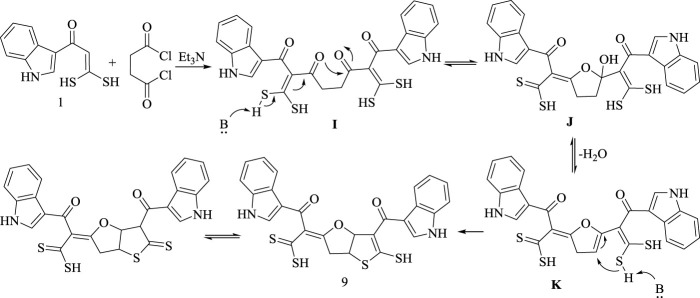
Synthetic route for compound 9.

Formation of the product **(9)** is assumed to proceed *via* acetylation of 2 mol of **(1)**, resulting in the intermediate **(I)**. Subsequently **(I)** subjects to intramolecular nucleophilic attack of carbonyl oxygen at C3 to carbonyl carbon at C6 followed by dehydration, forming species **(K)**. Finally, under the effect of basic catalyst species **(K)** undergoes to intramolecular sulfur attack to afford dihydrothienofuranoxopropandithioic acid derivative **(9)**, as declared in Scheme 10.

**SCHEME 11:**
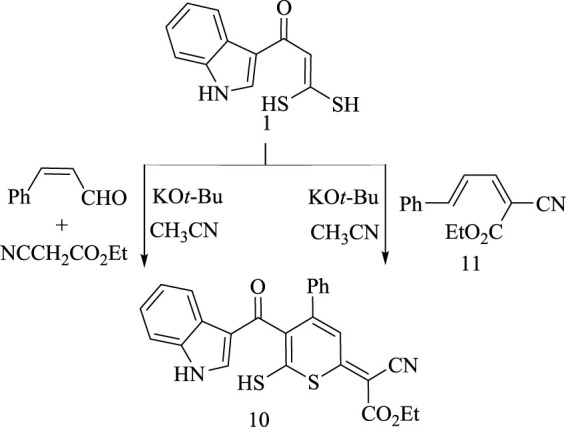
Procedures for the synthesis of product 10.

Based on the structural nature of α-oxoketene *gem*-dithiol **(1)**, a kind of olefinic substrate bearing a suitably placed sulfur-based nucleophilic group, it has been allowed to react with cinnamaldehyde and ethyl cyanoacetate through one-pot three-component reaction under basic conditions using K. *tert*.butoxide in acetonitrile to yield thiopyran derivative **(10)**, as shown in Scheme 11. Structure confirmation of **(10)** was identified by its elemental analysis and spectral data. ^1^H-NMR spectrum showed signals assigned for aromatic protons, ethyl group, and SH group. ^13^C-NMR showed peaks assigned for aromatic carbons, carbonyl carbon, and two aliphatic carbons. IR spectrum revealed the presence of carbonyl group and cyano group at 1,737 and 2,250 cm^−1^, respectively. Mass spectrum exhibited a molecular ionic peak *m/z* at 458.96. Also, to confirm the structure of the product **(10)** and to elucidate the reaction mechanism of α-oxoketene *gem*-dithiol **(1)** with cinnamaldehyde and ethyl cyanoacetate, a control experiment was performed. On treatment oxoketene *gem*-dithiol **(1)** with 2,4-pentadiene derivative **(11)** in the presence of K.*t*.BuO in acetonitrile afford a product identical to that resulted from the previous one pot three component reaction, as declared in Scheme 11.

**SCHEME 12:**
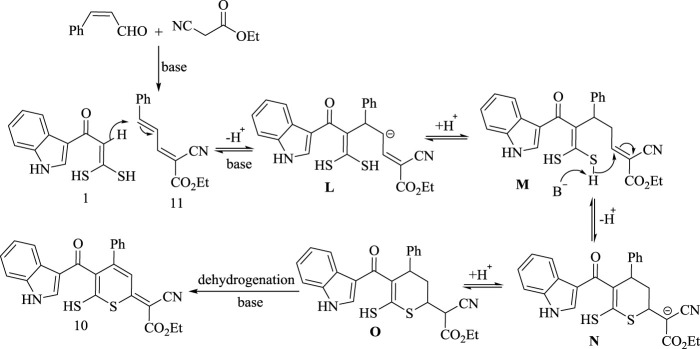
Synthetic strategy for compound 10.

Based on the experimental results, K. *tert*.butoxide promoted acceptor dehydrogenation plus actions as a basic catalyst reported in literature (Liu et al., 2019), the reaction mechanism is understood. Initially, 2,4-pentadiene derivative **(11)** is prepared *via* condensation of cinnamaldehyde and ethyl cyanoacetate under the effect of basic catalyst. There by, the intermolecular nucleophilic attack of C (*sp* ^*2*^)-H carbon in oxoketene *gem*-dithiol **(1)** to C5 in 2,4-pentadiene derivative **(11)** resulted in the formation of **(M)** which then undergoes to intramolecular sulfur attack to C5 forming anion **(N)** which finally undergoes adehydrogenation reaction, forming the desired product **(10)**, as declared in Scheme 12.

**SCHEME 13:**
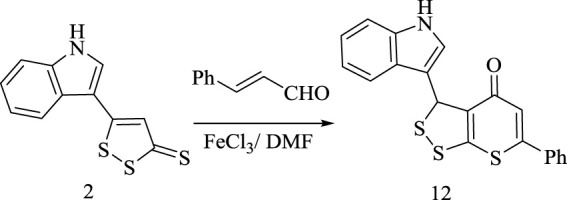
Synthesis of compounds 12.

Moreover, the reaction behavior of 1,2-dithiol-3-thione **(2)** with cinnamaldehyde under the effect of ferric chloride as a Lewis acid catalyst in DMF, was explored to afford 3-(1*H*-indol-3-yl)-6-phenyl-3*H*,4*H*-[1,2]dithiolo [3,4-*b*]thiopyran-4-one **(12)**, as shown in Scheme 13. Structural elucidation of **(12)** was confirmed based on its spectral and analytical data. ^1^H-NMR spectrum represented no olefinic C (*sp* ^*2*^)-H proton signal and showed peaks assigned for aromatic protons and aliphatic C (*sp* ^*3*^)-H proton. IR spectrum revealed the presence of carbonyl group at 1,661 cm^−1^. Mass spectrum showed a molecular ion peak *m/z* at 378.97.

**SCHEME 14:**
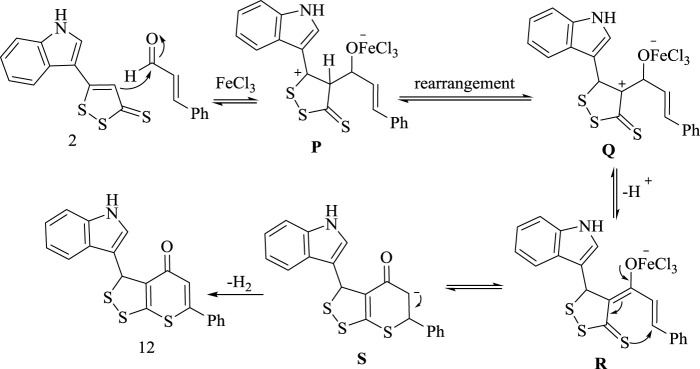
Suggested mechanism for the formation of compound 12.

The suggested synthetic route for the formation of product **(12)** is assumed to proceed *via* nucleophilic attack of C4 in 1,2-dithiol-3-thione **(2)** to the polarized carbonyl carbon in cinnamaldehyde under the effect of Lewis acid followed by rearrangement to form intermediate **(R)** which undergoes an intramolecular sulfur attack resulting in the formation of the thiopyranone derivative **(12)** *via* dehydrogenation of intermediat**e (S)**, as declared in Scheme 14.

### In Silico Docking Protocol

In an effort to elucidate the plausible mechanism by which these new synthesized chromenol, dihydroquinoline, and thiopyran compounds could exhibit their antimicrobial activity, in the present study, *in silico* molecular docking studies (Abdelmonsef et al., 2021; Abo-Bakr et al., 2021) on bacterial target enzyme UDP-N-acetylmuramatel-alanine ligase (MurC) (PDB ID: 2F00) and Human lanosterol 14α-demethylase (PDB ID: 6UEZ) were performed to demonstrate the mechanism of antibacterial and antifungal activity, and to get insights regarding the binding affinity and the intermolecular interactions of the newly synthesized compounds with the active sites of the target enzymes (Abdelmonsef et al., 2016; HA and SP, 2016; Dasari et al., 2017; Rondla et al., 2017; Haredi Abdelmonsef, 2019; Abdelmonsef and Mosallam, 2020; El-Maghraby and Abdelmonsef, 2020; El-Naggar et al., 2020; Haredi Abdelmonsef et al., 2020; Noser et al., 2020; Rashdan et al., 2020; El-Saghier et al., 2021; Sobhi et al., 2021). Ampicillin was used as a standard drug for in silico screening of the newly synthesized compounds. The docking study was carried out by using the PyRx-virtual screening tool. The molecular docking technique was achieved in a flexible docking mode which automatically generates nine confirmations for each docked molecule (Shehadi et al., 2020). The obtained data showed that all compounds were well accommodated with the active site of the enzymes (Table 1). The docked compounds were prioritized according to their binding energies after docking to active site pocket of the target protein as tabulated in Table 1. Amongst various compounds, the analogue **(9**) had the best docking score (−11.5 and −8.5 Kcal/mol, respectively) higher than of the standard drug against the target enzymes.

**TABLE 1:** The binding energies and molecular interactions between the docked compounds **1–12** with the prospective enzymes.

	Antibacterial			Antifungal		
	(ΔG_bind_)	Docked complex (amino acid–ligand) interactions	Distance (Å)	(ΔG_bind_)	Docked complex (amino acid–ligand) interactions	Distance (Å)
1	−5.4	**H-bonds**	2.11	−6.4	**π- π interaction**	3.72
		Asn296:OD1--compound 1			Phe234--compound 1	
		**π-sigma interaction**	3.68		Phe234--compound 1	3.98
		His292:CB--compound 1				
2	−6.1	**H-bonds**	2.49	−7.1	**π- π interaction**	3.93
		Asn296:OD1--compound 2			Phe234--compound 2	
		**π-sigma interaction**	3.61		Phe234--compound 2	3.97
		His292:CB--compound 2				
3	−7.3	**H-bonds**	2.23	−9.1	**H-bonds**	2.99
		Gln322:O--compound 3				
		Phe328:O--compound 3	2.28		Tyr145:OH--compound 3	
		**π- π interaction**				3.90
		Trp370--compound 3	5.66		**π- π interaction**	
		Trp370--compound 3	5.07			
		Trp370--compound 3	5.75		Phe234--compound 3	3.92
		Phe330--compound 3	3.61			
		Phe330--compound 3	4.37		Phe234--compound 3	
4	−7.2	**H-bonds**	2.45	−9.4	**π- π interaction**	3.99
		Asn287:OD1-compound4				
		**π- π interaction**	5.52		Phe234--compound 4	
		Trp370--compound 4	4.99			
		Trp370--compound 4				3.78
		Phe330--compound 4	3.90			
		**π-sigma interaction**			Phe234--compound 4	
		Gly323:CA--compound 11	3.91			
5	−5.5	**H-bonds**	2.48	−6.8	**H-bonds**	2.78
		Phe321:O--compound 5	4.99		Tyr145:OH--compound 5	3.01
		**π- π interaction**	3.96		Cys449:N--compound 5	
		Trp370--compound 5	5.43		Ile379:O--compound 5	2.29
		Trp370--compound 5			**π- π interaction**	
		Trp370--compound 5	5.55		Tyr131--compound 5	4.11
		Phe330--compound 5				
		Phe330--compound 5	5.41		Tyr 131--compound 5	3.78
6	−6.1	**H-bonds**	2.81	−6.9	**H-bonds**	3.01
		Lys157:NZ--compound 6				
		Phe330:N--compound 6	2.99		Tyr 145:OH--compound 6	3.16
		Asn287:OD1-compound6			Tyr 145:OH--compound 6	
		Phe328:O--compound 6	2.28		Tyr 145:OH--compound 6	2.92
		Gln322:O--compound 6	2.34		Tyr 145:OH--compound 6	
		Phe328:O--compound 6	2.31		Tyr 145:OH--compound 6	2.20
		**π- π interaction**	2.26		Cys449:N--compound 6	2.00
		Trp370--compound 6	5.55		Ile379:O--compound 6	3.11
		Trp370--compound 6	4.74		His447:O--compound 6	2.30
		Trp370--compound 6	5.17		**π- π interaction**	2.05
		Phe330--compound 6	4.31		Tyr131--compound 6	4.11
		Phe330--compound 6	3.72		Tyr131--compound 6	4.15
7	−7.3	**H-bonds**	2.33	−8.8	**H-bonds**	2.27
		Asn287:O--compound 7				
		**π- π interaction**	4.61		Ile379:O—compound7	
		Phe330—compound7				
		Phe330—compound7	3.85		**π- π interaction**	4.09
		Trp370---compound7	5.63		Tyr131—compound7	
		Trp370---compound7	5.36		Tyr131—compound7	3.94
		Trp370---compound7	4.22			
8	−7.4	**π- π interaction**	3.63	−8.5	**π- π interaction**	2.88
		Phe330—compound8			Phe234--compound 8	
					Phe234--compound 8	2.54
					**π-cation interaction**	4.29
		Trp370---compound8	5.67		Arg382:NH1-compound 8	4.33
		Trp370---compound8	5.06		Arg382:NH2-compound 8	
9	−8.5	**H-bonds**	2.89	−11.5	**H-bonds**	2.77
		Asn287:ND2—compound9			Tyr145:OH—compound9	
		**π- π interaction**	3.65			
		Phe330—compound9			Ile379:O—compound9	2.32
		Phe330—compound9	3.96			
		Trp370---compound9	5.54		**π- π interaction**	4.08
		Trp370---compound9	4.88		Tyr131—compound9	
		Trp370---compound9	4.29		Tyr131—compound9	3.92
		**π-sigma interaction**				
		Lys157---compound9	5.88		**π-cation interaction**	5.94
		Lys157---compound9	5.80		Arg382:NH1-compound9	
10	−7.5	**H-bonds**	3.17	−9.0	**H-bonds**	2.51
		Lys157:NZ---compound10				
		**π- π interaction**	4.49			
		Phe330—compound10			Tyr131:OH—compound10	
		Trp370---compound10	5.43			
12	−7.8	**π- π interaction**	5.06	−9.4	**π- π interaction**	3.99
		His292---compound12			Tyr131—compound12	
					Tyr131—compound12	3.89
Ampicillin (Reference drug)	−8.0	**π- π interaction**	5.37	−8.1	**H-bonds**	2.95
					Tyr 145:OH-- Ampicillin	
					Cys449:N—Ampicillin	2.99
					**π- π interaction**	4.12
		His292--- Ampicillin			Phe234—Ampicillin	

### Antibacterial Activity

**FIGURE 2:**
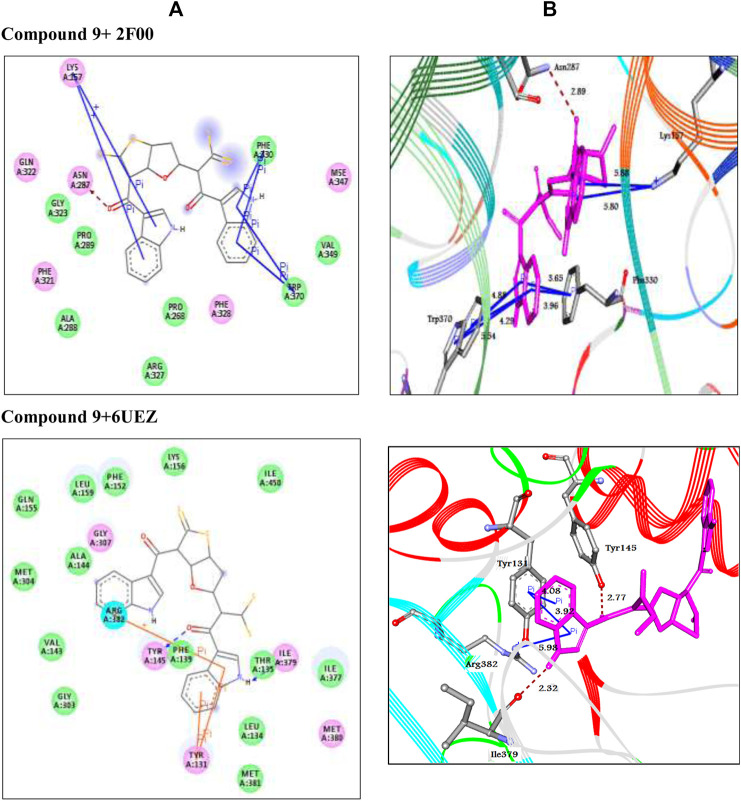
The interaction between analogue **(9**) with target enzymes. Left side representing 2D and the right side representing 3D complex enzyme-ligand interaction. In 2D, the docked compounds are represented by grey stick color, while the binding active residues are represented by three-letter codes. The HB interactions are shown by green and blue dotted lines while the π-stacking interactions are shown by orange lines. In the 3D representation, the docked compounds are represented by cyan stick models, while the binding active residues are represented by grey stick models. The HB interactions are shown by the black dotted line while the π-stacking interactions are shown by the orange line.

Mur family ligases possess a pivotal role in the bacterial cell wall peptidoglycans biosynthesis. MurC is the third enzyme in the Mur ligases of peptidoglycan pathway, initiates the addition of the first residue (l-alanine) onto the nucleotide precursor UDP-MurNAc. As MurC is functionally important for the bacterial survival, in the present work, *Escherichia coli* UDP-N-acetylmuramatel-alanine ligase (MurC) has been selected as attractive target for identification of antibacterial agents. As presented in Table 1 and Figure 2, the compound (9) exhibited good docking score (−11.5 Kcal/mol) as well as good intermolecular interaction network like H-bond, π-π, and π-cation stacking with the amino acid residues Asn287, Phe330, Trp370, and Lys157 at the distances of 2.89, 3.65, 3.96, 5.36, 4.88, 5.54, 4.29, 5.80, and 5.87 Å, respectively.

The 2D representations of other docked compounds and the standard drug with the target enzyme are shown in Supplementary Materials File as Supplementary Figure S1.

### Antifungal Activity

The fungal cytochrome P450 enzyme lanosterol14α-demethylase is responsible for the biosynthesis of sterol in eukaryotes and is the main target for azole antifungal candidates. To determine the fungal lanosterol 14α-demethylase inhibition of these compounds, human CYP51 enzyme (PDB code: 6UEZ) was selected as a target for treatment of fungal infections. The analogue **(9**) represented the minimum binding energy (−8.5 Kcal/mol) and showed a network of interactions. It formed four hydrogen bonds and two π-π stacked with the active site residues through Gln374, Thr437, Ser461, Val403, and Tyr404 at 2.98, 2.94, 2.97, 2.00, 5.11, and 4.35 Å, respectively as shown in Table 1 and Figure 2. The 2D representations of other docked compounds and the standard drug with the target enzyme are shown in the Supplementary Materials as Supplementary Figure S2.

### ADMET Predictions of the Compounds

The compound **(9**) with indole, thieno, and furan moieties revealed the most inhibitory effect against bacterial target enzyme UDP-N-acetylmuramatel-alanine ligase (MurC) (PDB ID: 2F00) and Human lanosterol 14α-demethylase (PDB ID: 6UEZ).

Towards understanding the pharmacokinetics properties of the newly synthesized compounds, the *in silico* tools admetSAR, and mol inspiration were used to calculate ADMET properties. The physicochemical and pharmacokinetic properties of all synthesized compounds are tabulated in Table 2. Firstly, the physicochemical properties were in agreement with the applied criteria, as the compounds had M. Wt in the ranges of (235.33–534.71 g/mol), indicating good absorption and orally bioavailable. The absorption percentage calculations showed high absorption percentage of values (97.19–100%). Hence, it can be concluded that the compounds possess good absorption and distribution properties.

**TABLE 2:** ADMET and drug-likeness properties of the prepared molecules **1–12**.

	1	2	3	4	5	6	7	8	9	10	12
Molecular Weight (g/mol)	235.33	249.37	481.16	529.29	325.41	415.48	336.44	368.51	534.71	458.55	379.53
Blood-Brain Barrier (BBB+)	0.96	0.97	0.66	0.89	0.88	0.80	0.94	0.90	0.89	0.58	0.98
Caco-2 Permeability (Caco2+)	0.53	0.56	0.54	0.56	0.61	0.65	0.59	0.56	0.56	0.57	0.56
%Human Intestinal Absorption (HIA+)	100	100	99.56	100	99.54	99.25	100	97.19	99.73	100	100
Logp	2.49	3.67	4.31	3.65	1.89	1.5	4.14	4.30	2.06	2.47	3.55
TPSA A^2^	110.46	104.36	101.12	134.65	160.15	154.23	116.11	134.29	103.23	147.05	111.70
HBA	2	2	3	3	5	8	3	3	5	5	2
HBD	1	1	2	2	3	6	1	2	2	1	1
N rotatable	2	1	2	1	6	8	2	1	5	6	2
Lipinski violations	0	0	0	0	1	1	0	0	1	1	0
Volume A^3^	194.97	193.71	307.25	330.19	270.19	343.79	278.18	291.97	424.89	388.80	307.78
Bioavailability score	0.55	0.55	0.55	0.55	0.55	0.55	0.55	0.55	0.55	0.55	0.55
GI absorption	High	High	High	Low	Low	Low	High	Low	High	Low	Low

HBA, number of hydrogen bond acceptors; HBD, number of hydrogen bond donors; logp, logarithm of partition coefficient between n-octanol and water; n rotatable, number of rotatable bonds; TPSA, topological polar surface area.

The acceptable ranges are as follows; Mol wt, (130–725); %Human intestinal absorption: >80% high, <25% low; Volume (500–2000); Donor HB (0.0–6.0); Accept HB (2.0–20.0); Predicted BBB permeability (−3 to 1.2); Predicted Caco cell permeability in nm/s (<25 is poor and >500 is great).

The topological polar surface area (TPSA) values for molecules were in acceptable range (below 140 A^2^) except for compounds **5**, **6**, and **10**, confirming that the compounds had considerable permeability into the cellular plasma membrane. All target compounds were in acceptable range for HBD (1–6), HBA (2–8) and rotatable bonds (1–8). Also, most compounds had high gastrointestinal (GI) absorption, confirming that they have excellent absorption possibility from the intensity after oral administration. All the ligands had good bioavailability with a score of 0.55, which is an indication that all compounds reach the circulation system easily. Additionally, the pharmaceutical properties of molecules exactly provided Lipinski’s rule of five. The results have indicated that the compounds are non-toxic and non-carcinogenic. The obtained results have showed that the molecules can exhibit good drug-likeness properties.

### Evaluation of Antimicrobial Activity

**FIGURE 3:**
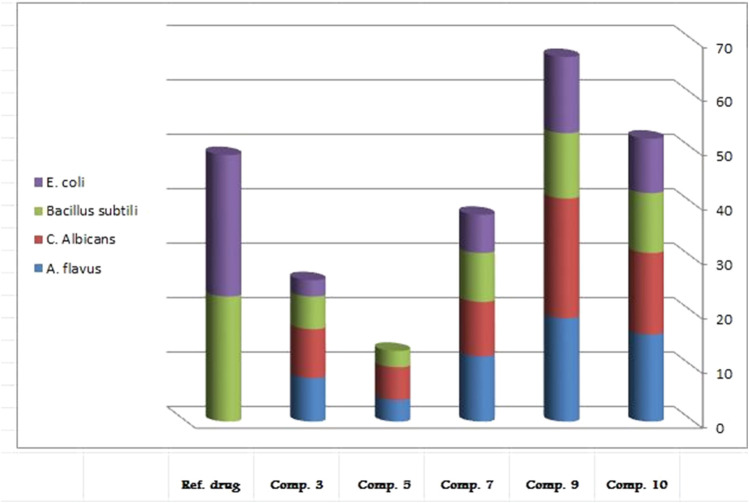
Antibacterial and antifungal activities of newly synthesized compounds.

Ampicillin was used as a standard drug for *in vitro* screening of the newly synthesized compounds. The results showed good to excellent activity against bacterial and fungal strains as documented in Table 3 and Figure 3. Compound **(9)** exhibited excellent activity against Gram-ve bacteria (*Escherichia coli*), and Gram + ve (*Bacillus subtilis*), as well as for antifungal activities against two fungi *Candida albicans* and *Aspergillus flavus* as compared to the standard drug, as shown as Supplementary Figure S43, in the Supplementary Materials File. The mechanism of killing of compound 9 against the tested microorganisms could be due to its effectively binding to bacterial cell membrane and inhibition of the active transport process which consequently causes bacterial cell death (Potbhare et al., 2019).

**TABLE 3:** Antimicrobial activity of some newly synthesized compounds.

Compound No	*E. coli*		*B. subtilis*		*C. Albicans*		*A. flavus*	
	Diameter of inhibition zone (mm)	% Activity index	Diameter of inhibition zone (mm)	% Activity index	Diameter of inhibition zone (mm)	% Activity index	Diameter of inhibition zone (mm)	% Activity index
10	10 ± 0.6	38.5	11 ± 0.6	47.8	15 ± 0.5	55.5	16 ± 0.6	64
9	14 ± 0.5	53.8	12 ± 0.7	52.2	22 ± 0.7	81.5	19 ± 0.4	76
7	7 ± 0.6	26.9	9 ± 0.5	39.1	10 ± 0.6	37	12 ± 0.6	48
5	NA	----	3 ± 0.2	13	6 ± 0.3	22.2	4 ± 0.2	16
3	3 ± 0.2	11.5	6 ± 0.3	26.1	9 ± 0.2	33.3	8 ± 0.3	32
Ampicillin (Reference drug)	26 ± 0.9	100	23 ± 0.8	100	NA	----	NA	----

NA; not available

## Experimental

### Materials and Equipment

All solvents and chemicals were commercially available from Sigma-Aldrich (USA). All melting points were determined on the Kofler melting point apparatus and were uncorrected. The progress of the reactions was followed up by the TLC technique. Infrared spectra (IR) were carried out using Bruker Tensor 37 spectrophotometer and absorption was expressed in wave number (cm^−1^) using KBr disc. ^1^H-NMR and ^13^C-NMR spectra were recorded on a Bruker Avance 400 MHz spectrometer, at Mansoura University, Faculty of Pharmacy, and Sohag University, using TMS as an internal standard; chemical shifts are expressed as δ and DMSO-*d*_6_ as solvent. Mass spectra were recorded on Shimadzu GCMS QP5050A spectrometer, at 70 eV (EI) at the regional center for mycology and biotechnology, Al-Azhar University, Egypt. Elemental analysis was carried out at the Microanalytical Center at Cairo University, Egypt.

### Synthesis of 1-(1H-indol-3-yl)-3,3-Dimercaptoprop-2-en-1-one

Carbon disulfide (1.80, 0.03 mol) was added to 3-acetylindole (4.78, 0.03 mol), in dry benzene (50 ml). The mixture was cooled to 0–5°C in an ice bath, with adding K. *tert*.butoxide (6.73 gm, 0.06 mol) with continuous shaking, then left over night in the fridge. Cold water was then added with continuous shaking of the previously prepared mixture, then it was agitated vigorously and poured into a separating funnel where it was divided into two layers (benzene layer and aqueous layer which contains the product as soluble salt). After separation, the aqueous layer was washed several times with petroleum ether and then it was acidified with cold concentrated sulfuric acid. The solution was left in the refrigerator to settle the precipitate, which was collected by filtration and recrystallized from benzene to afford the desired product **(1)** as orange crystals, mp. 140–142°C, yield 76%. Mass spectrum (*m/z*): 234.89. Elemental analysis for C_11_H_9_NOS_2_, M. Wt. 235.32; Cal. %: C, 56.14; H, 3.85; N, 5.95; S, 27.25; Found%: C, 55.89; H, 3.92; N, 5.75; S, 27.06.^1^H-NMR (DMSO-*d*_*6*_): δ 2.46 (s, 2H, 2SH), 6.77 (s, 1H, C(*sp* ^*2*^)-H), 7.15–8.29 (m, *J* = 8, 2d, *J* = 8, 5H, aromatic-H) and 11.88 (s, 1H, NH). IR (cm^−1^): 1,665 for C=O and 3,159 for NH. ^13^C-NMR (DMSO): δ 111.48, 119.31, 122.45, 122.71, 127.55, 129.49, 137.42, 139.43, 145.51, 198.49.

### Synthesis of 5-(1H-Indol-3-yl)-3H-1,2-Dithiole-3-Thione

To oxoketene *gem*-dithiol **(1)** (0.94 gm, 0.004 mol) in (50 ml) dry benzene, phosphorous pentasulfide (5.33 gm, 0.012 mol) was added. The reaction mixture was heated in a boiling water bath for 5 h, and then it was filtered while hot. The clear benzene layer was concentrated to half of its volume followed by the addition of appropriate quantity of petroleum ether. The precipitated solid product was filtered off and washed twice with petroleum ether and recrystallized from benzene to afford the desired compound **(2)** as red crystals, mp. 120–122°C, yield 82%. Mass spectrum (*m/z*): 249.10. Elemental analysis for C_11_H_7_NS_3_, M. Wt. 249.37; Cal. %: C, 52.98; H, 2.83; N, 5.62; S, 38.57; Found%: C, 53.14; H, 2.75; N, 5.85; S, 38.23.^1^H-NMR (DMSO-*d*_*6*_): δ 5.93 (s, 1H, C (*sp* ^*2*^)-H), 7.16–8.86 (m, *J* = 4, d, *J* = 4, 5H, aromatic-H), 11.96 (1H, NH), IR (cm^−1^): 1,143 for C=S and 3,385 for NH. ^13^C-NMR (DMSO): δ111.23, 112.34, 119.63, 120.66, 121.92, 127.84, 132.91, 139.75, 181.34, 198.23.

### Synthesis of (6,8-Dibromo-4-Hydroxy-2-Mercapto-4H-chromen-3-yl) (1H-indol-3-yl) Methanone

A mixture of 3,5-dibromosalicyldehyde (0.28 gm, 0.001 mol) and oxoketene *gem*-dithiol **(1)** (0.24 gm, 0.001 mol) in (20 ml) DMF was refluxed in DMF (20 ml) in the presence of Et_3_N for 6 h. The mixture was concentrated and left to cool at RT, the precipitate obtained was separated by filtration and recrystallized from ethanol to give the desired product **(3)** as brown crystals, mp. 180–182°C, yield 86%. Mass spectrum (*m/z*): 481.22. Elemental analysis for: C_18_H_11_Br_2_NO_3_S, M. Wt. 481.14; Cal. %: C, 44.93; H, 2.30; Br, 33.21; N, 2.91; S, 6.66; Found %: C, 44.69; H, 2.33; N, 2.73; Br, 33.42; S, 6.91.^1^H-NMR (DMSO-*d*_*6*_): δ 2.46 (s, 1H, SH), 2.90 (s, 1H, C(*sp* ^*3*^)-H), 3.27 (s, 1H, OH), 6.99–8.27 (m, *J* = 8, 7H, aromatic-H) and 10.22 (s, 1H, NH). IR (cm^−1^): 1,650 for C=O, 3,121 for NH and 3,054 for OH. ^13^C-NMR (DMSO): δ 55.02, 110.37, 110.49, 111.21, 111.91, 112.57, 113.25, 117.25, 117.76, 118.11, 118.19, 118.71, 119.27, 119.83, 120.39, 120.70, 121.42, 121.61, 121.79, 122.13, 122.80, 123.01, 123.19, 123.55, 124.01, 124.48, 125.75, 126.68, 127.13, 127.62, 129.51, 129.79, 130.91, 131.35, 131.92, 134.15, 134.89, 135.27, 136,76, 137.08, 137.12, 137.48, 138.96, 160.37,162.79, 190.12.

### Synthesis of 6,8-Dibromo-3-(1H-indol-3-yl)-9a-Mercapto-3H,9aH-[1,2]Dithiolo[3,4-b] Chromen-4-ol

A mixture of 1,2-dithiole-3-thione **(2)** (0.25 gm, 0.001 mol), 3,5-dibromosali-cyldehyde (0.28 gm, 0.001 mol) and Et_3_N in absolute ethanol (30 ml) was heated under reflux for 8 h, then the solution was concentrated to a third of its volume and left to cool at RT. The precipitated product was collected and recrystallized from methanol to yield the desired compound **(4)** as brown crystals, mp. 120–122°C, yield 61%. Mass spectrum (*m/z*): 528.74. Elemental analysis for C_18_H_11_Br_2_NO_2_S_3_, M. Wt. 529.29; Cal. %: C, 40.84; H, 2.09; Br, 30.19; N, 2.65; S, 18.17; Found%: C, 40.64; H, 1.96; N, 2.88; S, 18.11.^1^H-NMR (DMSO-*d*_*6*_): δ 1.19 (s, 1H, SH), 3.06 (s, 1H, C(*sp* ^*3*^)-H), 7.02–8.26 (m, *J* = 8, 7H, aromatic-H) and 10.22–10.23 (s, 1H, OH+1H, NH). IR (cm^−1^): 2,676 for SH, 3,238 for NH and 3,412 for OH. ^13^C-NMR (DMSO): δ 45.92, 110.89, 119.03, 120.18, 120.51, 120.80, 121.22, 124.44, 124.71, 129.09, 130.19, 130.53, 130.85, 131.92, 132.69, 135.28, 138.44, 138.76, 139.06, 140.71, 160.45, 189.90.

### Synthesis of 2-((1,3-Dihydroxypropan-2-yl)oxy)-1-(1H-Indol-3-yl)-3,3-Dimercaptopr-op-2-en-1-one

Oxoketene *gem*-dithiol **(1)** (0.47 gm; 0.002 mol) was heated under reflux for 7 h with glycerol (0.15 ml; 0.002 mol) in DMF (20 ml) and a catalytic amount of ferric chloride hexahydrate. The reaction mixture was concentrated, cooled at RT and the formed precipitate was filtered off and recrystallized from ethanol to give the product **(5)** as dark brown crystals, mp. 110–112°C, yield 83%. Mass spectrum (*m/z*): 326.68. Elemental analysis for C_14_H_15_N O_4_S_2_, M. Wt. 325.40; Cal. %: C, 51.67; H, 4.65; N, 4.30; S, 19.70; Found %: C, 51.59; H, 4.52; N, 3.97; S, 19.44.^1^H-NMR (DMSO-*d*_*6*_): δ 1.06 (s, 2H, 2OH), 2.44 (s, 2H, 2SH), 3.31–3.44 (br, 5H, C(*sp* ^*3*^)-H), 7.16–8.27 (m, *J* = 8, 2d, *J* = 8, s, 5H, aromatic-H) and 11.89 (s, 1H, NH), IR (cm^−1^): 1708 for CO, 3,162 for NH and 3,360 for OH. ^13^C-NMR (DMSO): δ 56.77, 63.67, 72.99, 111.37, 111.88, 112.79, 116.28, 117.22, 121.79, 122.29, 123.35, 125.71, 126.92, 135.21, 137.06, 193.23.

### Synthesis of 2-(Dimercaptomethylene)-3,4,5,6,7,8-Hexahydroxy-1-(1H-indol-3-yl)oct-an-1-one

To oxoketene *gem*-dithiol **(1)** (0.24 gm, 0.001 mol) in DMF (20 ml), glucose (0.18 gm, 0.001 mol) and a catalytic amount of ferric chloride hexahydrate were added. The reaction mixture was refluxed for 5 h, then the solution was concentrated to a third of its volume and left to cool at RT. The precipitated product was collected by filtration and recrystallized from DMF to afford the desired compound **(6)** as a brown powder, mp. 295–297°C, yield; 65%. Mass spectrum (*m/z*): 415.73. Elemental analysis for: C_17_H_21_NO_7_S_2_, M. Wt. 415.48, Cal. %: C, 49.14; H, 5.09; N, 3.37; S, 15.43; Found %: C, 49.21; H, 5.16; N, 3.19; S, 15.59.^1^H-NMR (DMSO-*d*_*6*_): δ 2.74 (s, 2H, SH), 2.90 (s, 7H, C(*sp* ^*3*^)-H), 3.26 (s, 6H, OH), 7.19–8.27 (m, *J* = 8, 5H, aromatic-H) and 11.85 (1H, NH). IR (cm^−1^): 1,659 for C=O, 3,265 and 3,395 for OH group. ^13^C-NMR (DMSO): δ 31.27, 36.24, 112.52, 121.77, 162.79.

### Synthesis of 2-(1H-Indol-3-yl)-4-oxo-3,4-Dihydroquinoline-3-Carbodithioic Acid

A mixture of oxoketene *gem*-dithiol **(1)** (0.24 gm, 0.001 mol) and anthranilic acid (0.14 gm, 0.001 mol) in glacial acetic acid (30 ml) was refluxed for 12 h, then the solution was concentrated to its third volume and left to cool at RT. The precipitated product was collected by filtration and recrystallized from ethanol to give the desired compound **(7)** as brown crystals, mp. 120–123°C, yield 93%. Mass spectrum (*m/z*): 335.03. Elemental analysis for: C_18_H_12_N_2_OS_2_, M. Wt. 336.43; Cal. %: C, 64.26; H, 3.60; N, 8.32; S, 19.06; Found %: C, 64.32; H, 3.48; N, 8.21; S, 19.39.^1^H-NMR (DMSO-*d*_*6*_): δ 2.14 (s, 1H, C (*sp* ^*3*^)-H), 2.46 (s, 1H, SH), 7.16–8.28 (m, *J* = 4, 3d, *J* = 8, 9H, aromatic-H) and 11.87 (1H, NH). IR (cm^−1^): 1,439 for C=S, 1,677 for C=O and 3,169 for NH. ^13^C-NMR (DMSO): δ 27.70, 112.51, 117.30, 120.42, 121.78, 122.07, 122.97, 123.14, 125.77, 126.73, 131.49, 134.31, 134.70, 137.13, 141.30, 168.87, 169.89, 193.03.

### Synthesis of 3-(1H-Indol-3-yl)-9a-Mercapto-9,9a-Dihydro-4H-[1,2]Dithiolo[3,4-b]qui-Nolin-4-one

A mixture of 1,2-dithiole-3-thione **(2)** (0.50 gm, 0.002 mol) and anthranilic acid (0.27 gm, 0.002 mol) in glacial acetic acid (30 ml) was refluxed for 8 h, then the solution was concentrated to its fourth volume and left to cool at RT. The precipitated product was collected by filtration and recrystallized from ethanol to afford the desired adduct **(8)** as brown crystals, mp. 170–172°C, yield 55%. Mass spectrum (*m/z*): 367.33. Elemental analysis for C_18_H_12_N_2_OS_3_, M. Wt. 368.49; Cal. %: C, 58.66; H, 3.28; N, 7.60; S, 26.10; Found %: C, 58.39; H, 3.38; N, 7.51; S, 26.42.^1^H-NMR (DMSO-*d*_*6*_): δ 2.14 (s, 1H, SH), 3.90 (s, 1H, NH hydroquinolone), 7.12–8.47 (3m, *J* = 4, d, *J* = 8, 9H, aromatic-H) and 11.02 (s, 1H, NH). IR (cm^−1^): 1,698 for C=O, 3,183 for NH and 3,419 for NH. ^13^C-NMR (DMSO): δ 52.89, 112.53, 113.32, 116.83, 117.23, 117.84, 119.42, 119.85, 120.33, 121.78, 122.10, 122.96, 123.16, 125.74, 129.10, 130.42, 131.21, 131.51, 131.96, 132.17, 134.44, 134.83, 136.12, 137.10, 141.35, 160.55, 168.93, 172.55.

### Synthesis of €-2-(6-(1H-Indole-3-Carbonyl)-5-Thioxotetrahydrothieno[3,2-b]Furan-2(3H)-Ylidene)-3-(1H-Indol-3-Yl)-3-Oxopropanedithioic Acid

To a solution containing 2:1 M ratio of oxoketene *gem*-dithiol **(1)** (0.47 gm, 0.002 mol) in THF (25 ml) and succinyl dichloride (0.11 ml, 0.001 mol), respectively, a catalytic amount of Et_3_N (0.3 ml) was added. The mixture was left for 2 h in fridge and then it was heated under reflux for 5 h. The reaction mixture was concentrated, cooled at RT and the formed precipitate was filtered and recrystallized from methanol to yield the desired compound **(9)** as red crystals, mp. 155–157°C, yield 70%. Mass spectrum (*m/z*): 533.72. Elemental analysis for C_26_H_18_N_2_O_3_S_4_, M. Wt. 534.69; Cal. %: C, 58.40; H, 3.39; N, 5.23; S, 23.99; Found %: C, 58.28; H, 3.26; N, 5.33; S, 23.78 ^1^H-NMR (DMSO-*d*_*6*_): δ 1.07–1.37 (m, *J* = 4, 1H, C (*sp* ^*3*^)-H) 1.65–1.77 (m, *J* = 4, 2H, CH_2_), 2.46 (s, 1H, SH), 3.09–3.10 (br, 1H, C(*sp* ^*3*^)-H), 3.61–3.66 (br, 1H, C (*sp* ^*3*^)-H), 7.–7 - 8.27 (m, *J* = 8, d, *J* = 8, 10H, aromatic-H), and 11.88 (s, 2H, 2NH). IR (cm^−1^): 1,616, 1714 for 2’ = O’s and 3,262, 3,393 for ‘NH’s. ^13^C-NMR (DMSO): δ 45.47, 45.49, 63.71, 67.50, 111.83, 112.60, 114.32, 114.99, 117.23, 118.42, 118.69, 119.51, 121.11, 121.80, 122.13, 122.79, 123.18, 123.97, 125.76, 127.06, 127.75, 129.57, 130.39, 134.88, 135.67, 136.52, 136.91, 137.15, 137.51, 138.19, 172.42, 172.64, 173.97, 174.16, 193.21.

### Synthesis of ethyl(z)-2-(5-(1H-Indole-3-Carbonyl)-6-Mercapto-4-Phenyl-2h-Thiopyr-an-2-Ylidene)-2-Cyanoacetate

Method 1: A mixture of three components, oxoketene *gem*-dithiol **(1)** (0.24 gm, 0.001 mol), cinnamaldehyde (0.13 ml, 0.001 mol) and ethyl cyanoacetate (0.13 ml; 0.001 mol) in acetonitrile (20 ml) and a catalytic amount of K. *tert*.butoxide was heated under reflux for 5 h, then the solution was concentrated and left to cool at RT. The precipitated product was collected by filtration and recrystallized from ethanol to afford the desired compound **(11)** as brown crystals, mp. 130–132°C, yield 88%.

Method 2: A mixture of oxoketene *gem*-dithiol **(1)** (0.24 gm, 0.001 mol), and 2,4-pentadiene derivative **(11)** (0.23 gm, 0.001 mol) in acetonitrile (30 ml) and K. *tert*.butoxide was heated under reflux for 4 h, then the solution was concentrated and left to cool at RT. The precipitated product just formed was collected by filtration, and recrystallized from ethanol to yield the desired adduct **(10)** as brown crystals, mp. 130–132°C, yield 51%. Mass spectrum (*m/z*): 458.96. Elemental analysis for C_25_H_18_N_2_O_3_S_2_, M. Wt. 458.55; Cal. %: C, 65.48; H, 3.96; N, 6.11; S, 13.99; Found %: C, 65.55; H, 3.74; N, 5.88; S, 13.57.^1^H-NMR (DMSO-*d*_*6*_): δ 1.06–1.09 (t, *J* = 8, 3H, CH_3_), 2.46 (s, 1H, SH), 3.44–3.49 (q, *J* = 8, 2H, CH_2_), 7.–6 - 8.28 (m, *J* = 8, d, *J* = 8, d, *J* = 4, 10H, aromatic-H + 1H, thiopyran-H) and 11.98 (1H, NH). IR (cm^−1^): 1737 for C=O, 2,250 for CN and 3,399 for NH. ^13^C-NMR (DMSO): δ 27.70, 56.51, 63.62, 112.52, 117.30, 121.78, 122.06, 123.14, 125.78, 128.96, 129.40, 134.70, 137.14, 193.03.

### Synthesis of 3-(1H-indol-3-yl)-6-phenyl-3H,4H-[1,2]dithiolo[3,4-b]thiopyran-4-one (12)

A mixture of 1,2-dithiole-3-thione **(2)** (0.25 gm, 0.001 mol) and cinnamaldehyde (0.13 gm, 0.001 mol) in DMF (20 ml) and a catalytic amount of ferric chloride hexahydrate was heated under reflux for 4 h, then the solution was concentrated and left to cool at RT. The separated solid product was collected by filtration and recrystallized from chloroform/ethanol (1:1) to afford the desired product **(12)** as brown crystals, mp. 100–102°C, yield 85%. Mass spectrum (*m/z*): 378.97. Elemental analysis for C_20_H_13_NOS_3_, M. Wt. 379.51; cal. %: C, 63.26; H, 3.45; N, 3.69; S, 25.34; Found %: C, 63.33; H, 3.48; N, 3.56; S, 25.16.^1^H-NMR (DMSO-*d*_*6*_): δ 3.26 (s, 1H, C (*sp* ^*3*^)-H), and 7.26–7.61 (m, *J* = 8, 10H, aromatic-H + 1H, C (*sp* ^*2*^)-H). IR (cm^−1^): 1,661 for C=O and 3,421 for NH. ^13^C-NMR (DMSO): δ 34.23, 111.34, 112.40, 120.31, 123.45, 125.56, 128.36, 136.37, 163.61, 150.38, 183.43, 190.39.

### Molecular Docking Study

The docking protocol has been applied both to study the binding mode of all compounds in the active sites of UDP-N-acetylmuramatel-alanine ligase (MurC), and Human lanosterol14α-demethylase enzymes, and to understand the antimicrobial mechanism (Bagade et al., 2019; Chaudhary et al., 2019). A dataset of the target compounds were sketched using ChemDraw Ultra 7.0, then converted to SDF format using open Babel GUI tool (O’Boyle et al., 2011). The X-ray crystal structures of the target enzymes were retrieved from the PDB Data Bank (Berman et al., 2002). In addition, the energy of the compounds and targets was minimized by using UFF Force Field (Rappé et al., 1992) in Open Babel and CHARMM Force Field (Brooks et al., 2009) in Discovery Studio, respectively. The *in silico* molecular docking studies were accomplished using PyRx-virtual screening tool (Dallakyan and Olson, 2015).

### *In Silico* Physicochemical and Pharmacokinetic Prediction

To predict the physicochemical and pharmacokinetic properties of all synthesized compounds, the free available websites such as admetSAR, SwissADME, and Mol inspiration were used.

### Antimicrobial Activity

The anti-microbial activity of the synthesized compounds was tested against Gram-positive bacteria (*Bacillus subtilis*), Gram-negative bacteria (*Escherichia coli*), and two fungi strains (*Candida albicans,* and *Aspergillus flavus*) by using disc diffusion method (Heatley, 1944). Each of the compounds was dissolved in DMSO and a solution of the concentration 1 mg/ml was prepared separately. Whatman filter paper discs were prepared with a standard size (5 cm), and they were cut and sterilized in an autoclave. The paper discs soaked in the desired concentration of the complex solution were places aseptically in the Petri dishes containing nutrient agar media (agar 20 g + beef extract 3 g + peptone 5 g) seeded with *Bacillus subtilis, E. coli, Candida albicans and Aspergillus flavus.* The Petri dishes were incubated at 36°C and the inhibition zones were recorded after 24 h of incubation. Each treatment was replicated three times. The antibacterial activity of a common standard antibiotic ampicillin was also recorded using the same procedure as above at the same concentration and solvents. The % activity index for the complex was calculated as:

$$\%\mathrm{Activity}\;\mathrm{Index}=\frac{\mathrm{Zone}\;\mathrm{of}\;\mathrm{inhibition}\;\mathrm{by}\;\mathrm{test}\;\mathrm{compound}\left(diametre\right)}{\mathrm{Zone}\;\mathrm{of}\;\mathrm{inhibition}\;\mathrm{by}\;\mathrm{standard}\left(diametre\right)}$$

## Conclusion

In this study, a series of eleven novel compounds bearing heterocycles such as chromenol, dihydroquinoline, and thiopyran moieties-based indole core were synthesized and characterized by means of elemental and spectral analyses. Further, *in silico* docking studies for all newly synthesized compounds were performed against the target enzymes UDP-N-acetylmuramatel-alanine ligase (MurC), and human lanosterol14α-demethylase, both to study their binding affinities and to identify the mechanism of antimicrobial activity. In addition, all newly synthesized compounds were evaluated for their *in vitro* antibacterial and antifungal activities. The *in silico* and *in vitro* findings represented that compound **(9**) with thieno and furan moieties attached to indole core, was the most biologically active molecule against bacterial and fungi strains. Therefore, it could serve as the lead for further optimization to arrive at potent molecules targeting the microbial diseases Rashdan et al., 2021.

## Data Availability

The original contributions presented in the study are included in the article/Supplementary Material, further inquiries can be directed to the corresponding author.
