# Supplementary material for: Synthesis, Molecular Docking Analysis and in Vitro Biological Evaluation of Some New Heterocyclic Scaffolds-Based Indole Moiety as Possible Antimicrobial Agents
Source: Front Mol Biosci. 2022 Jan 17;8:775013. doi: 10.3389/fmolb.2021.775013 (PMC8801890; doi:10.3389/fmolb.2021.775013)
Supplement: Supplementary file 1 [file DataSheet1.PDF]

Supplementary data (Figure S1- Figure S43)

Synthesis, Molecular Docking Analysis and In vitro Biological Evaluation of Some New Heterocyclic Scaffolds-based Indole Moiety as Possible Antimicrobial Agents

Entesar A. Hassan[1], Ihsan A. Shehadi[2], Awatef M. Elmaghraby[1], Hadir M. Mostafa[1], Salem E. Zayed[1], Aboubakr H. Abdelmonsef [1,*]

[1] Chemistry Department, Faculty of Science, South Valley University, Qena 83523, Egypt

[2] Chemistry Department, Faculty of Science, University of Sharjah, Sharjah 27272, UAE

*Correspondence; aboubakr.ahmed@sci.svu.edu.eg

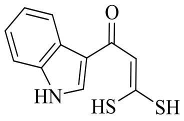

1

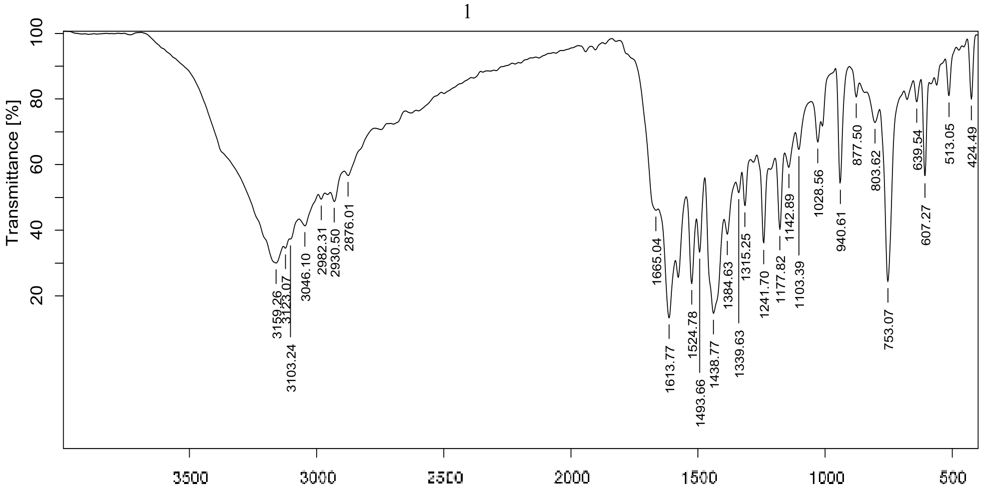

Figure 1: IR analysis of 1

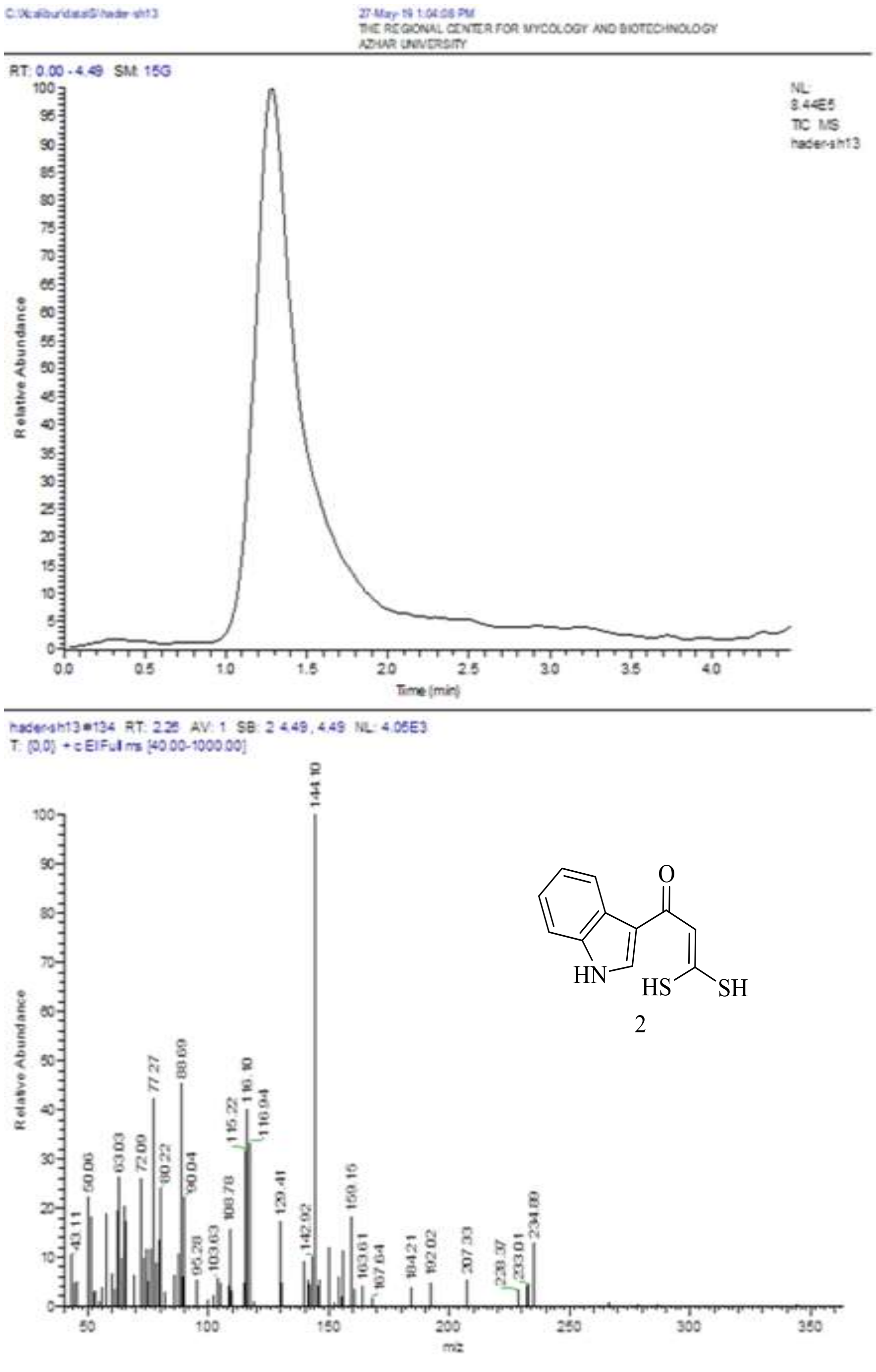

Figure 2: Mass analysis of 2

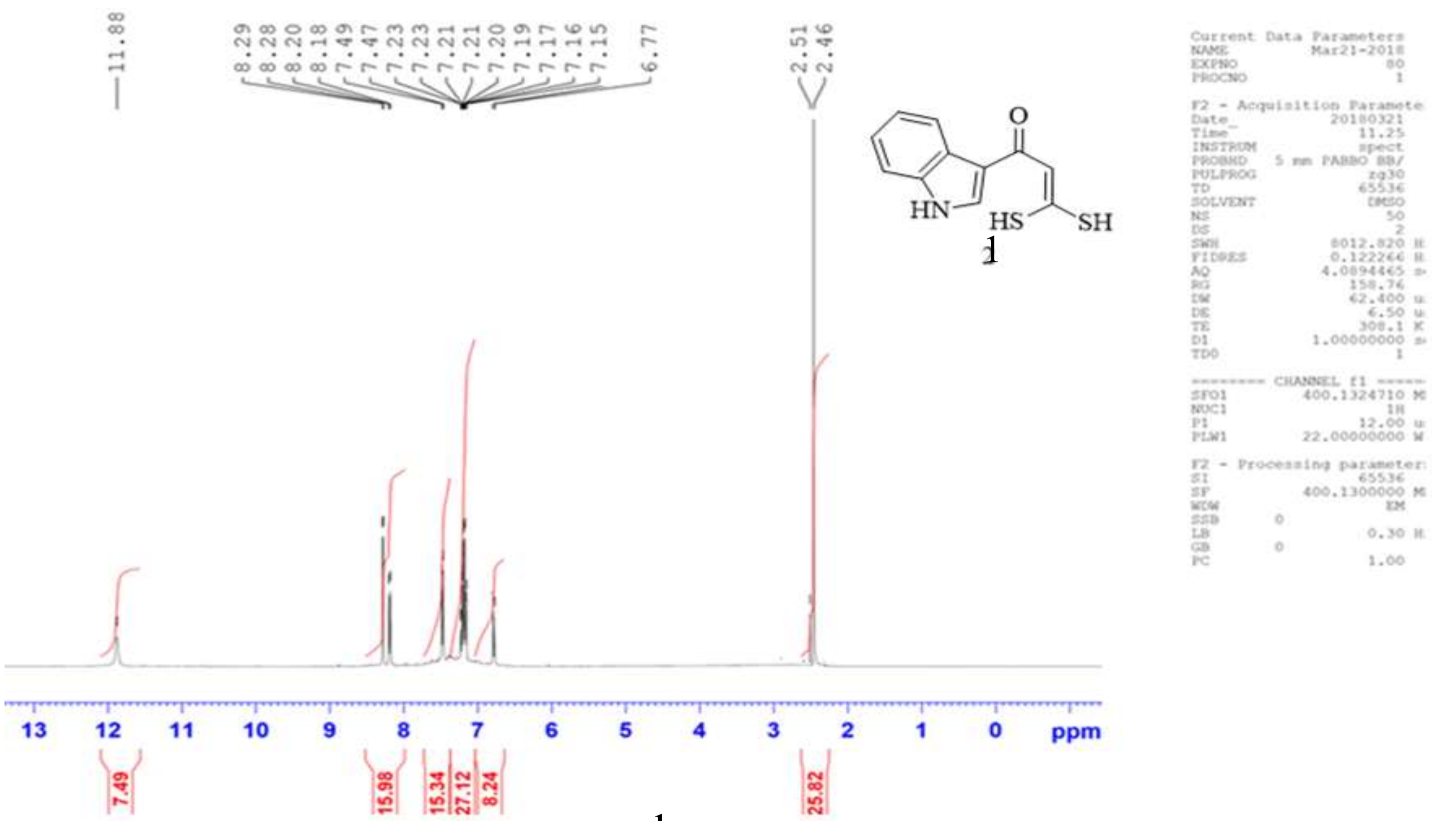

Figure 3: ^{1}HNMR analysis of 1

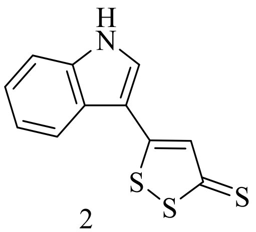

2

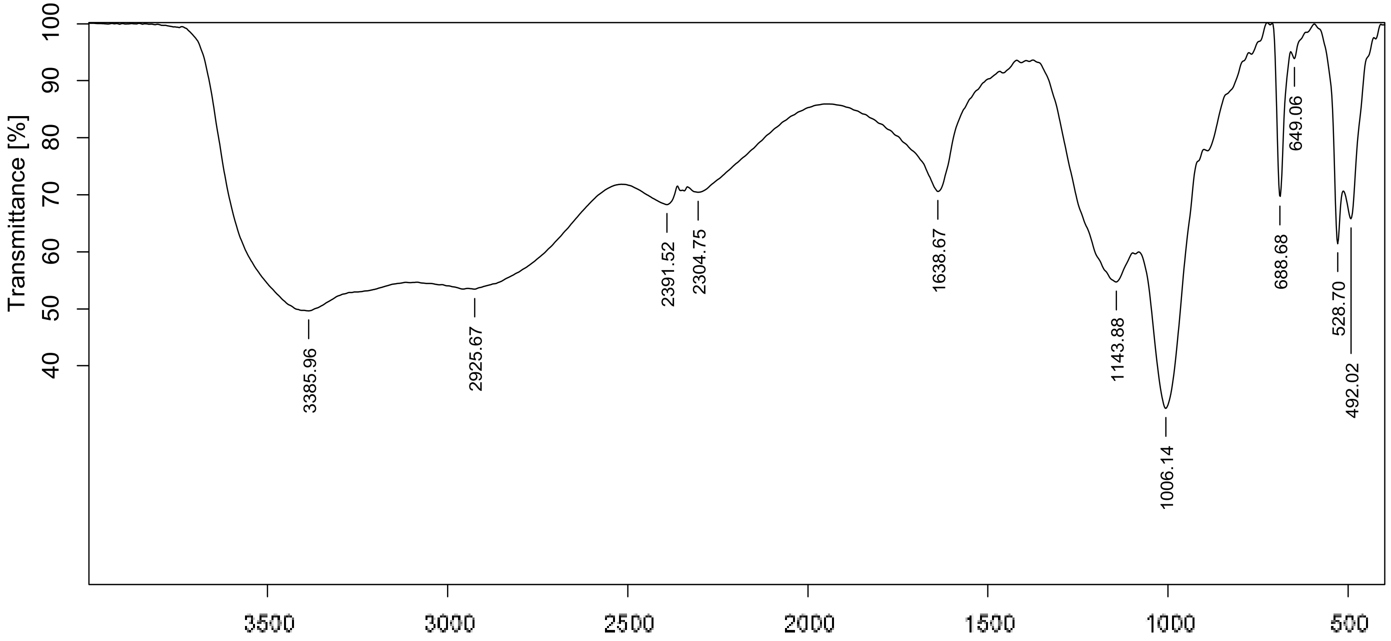

Figure 4 : IR analysis of 2

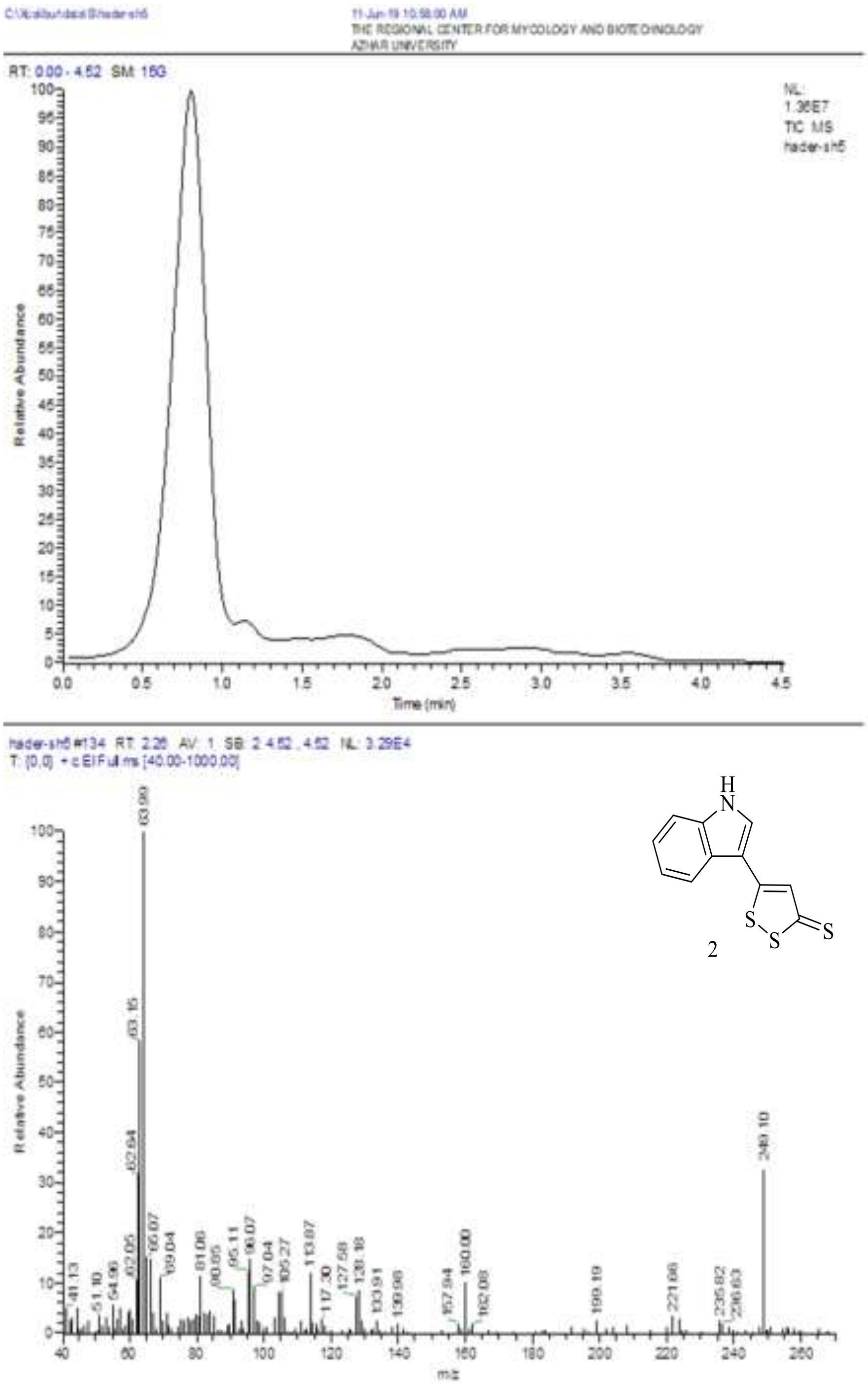

Figure 5: Mass analysis of 2

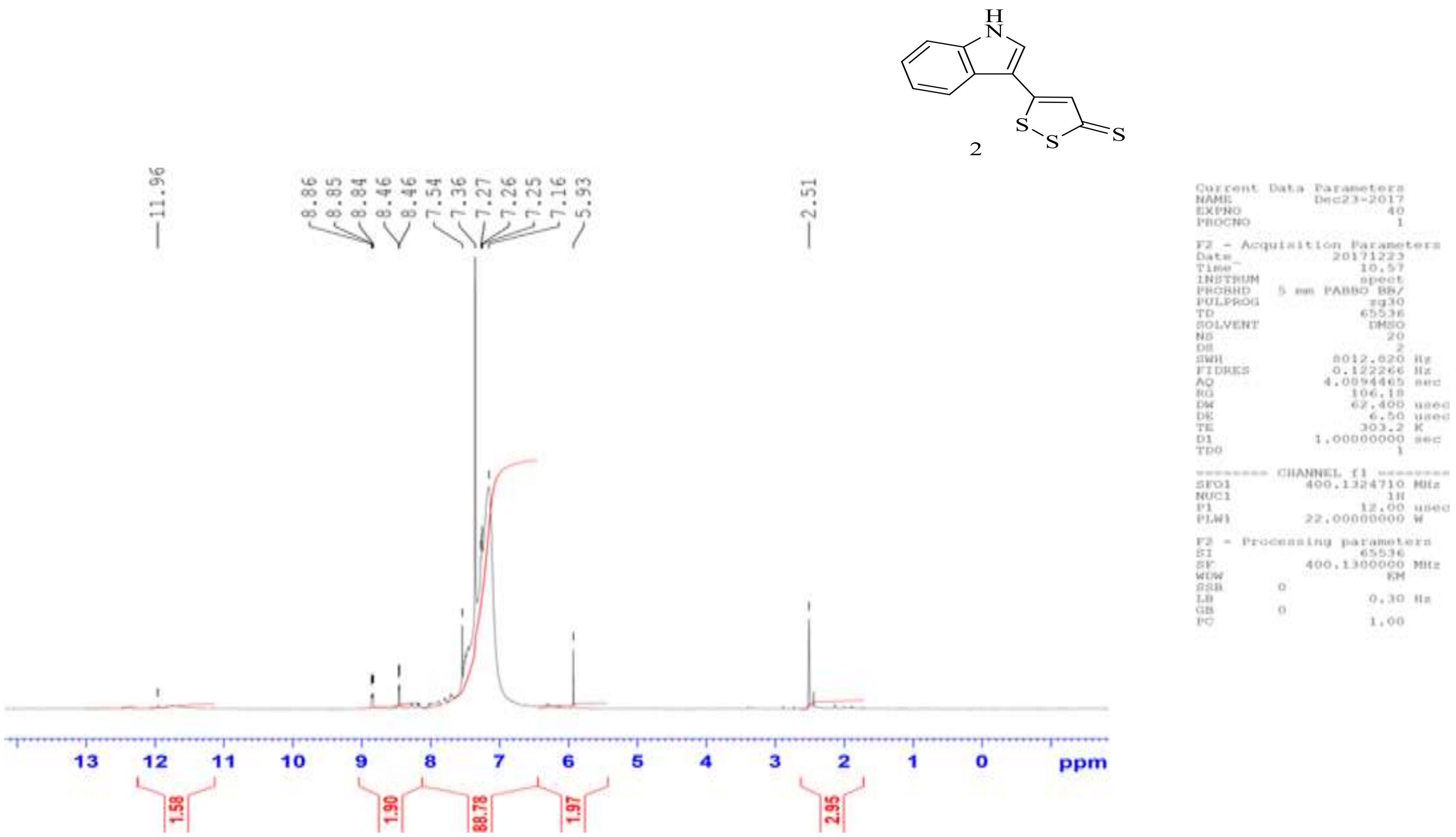

Figure 6 : ^{1}HNMR analysis of 2

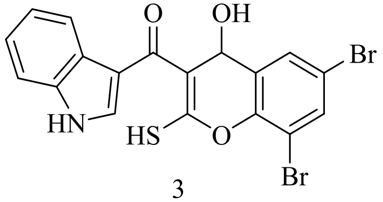

3

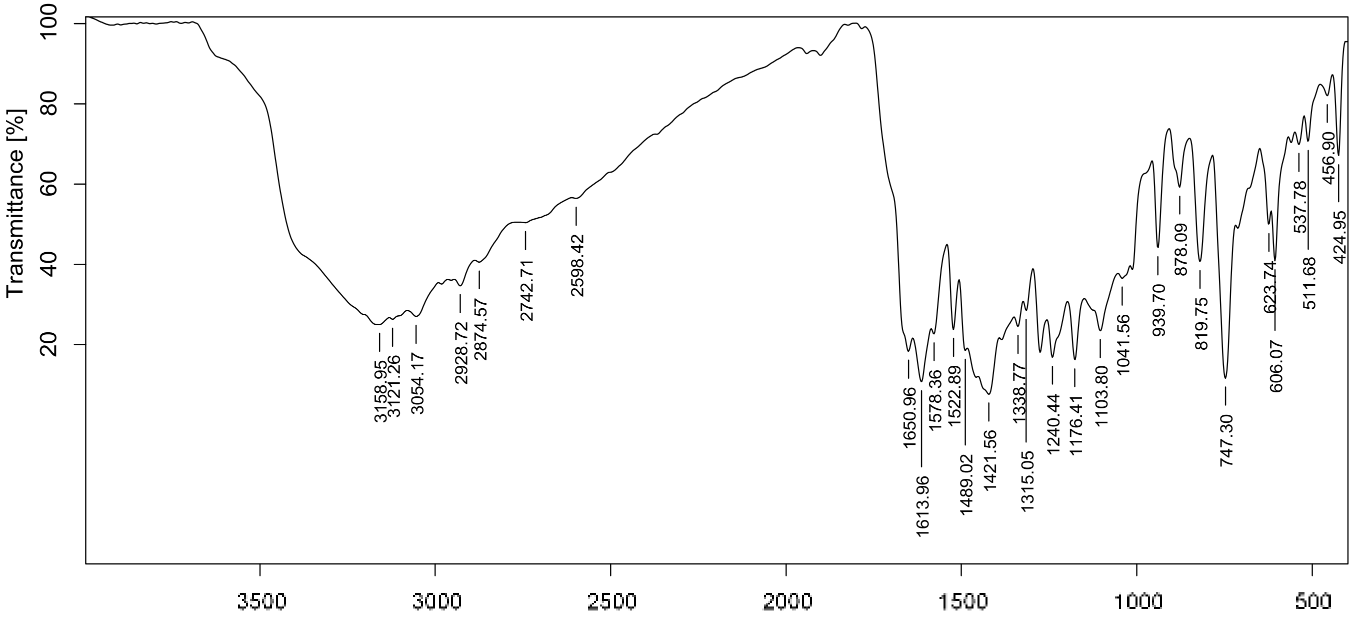

Figure 7: IR analysis of 3

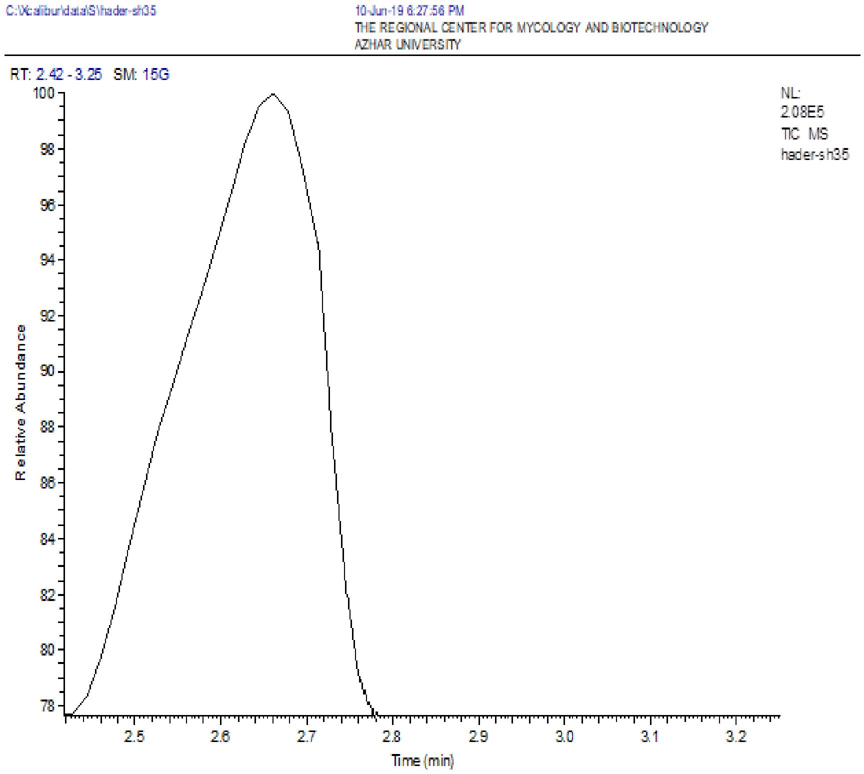

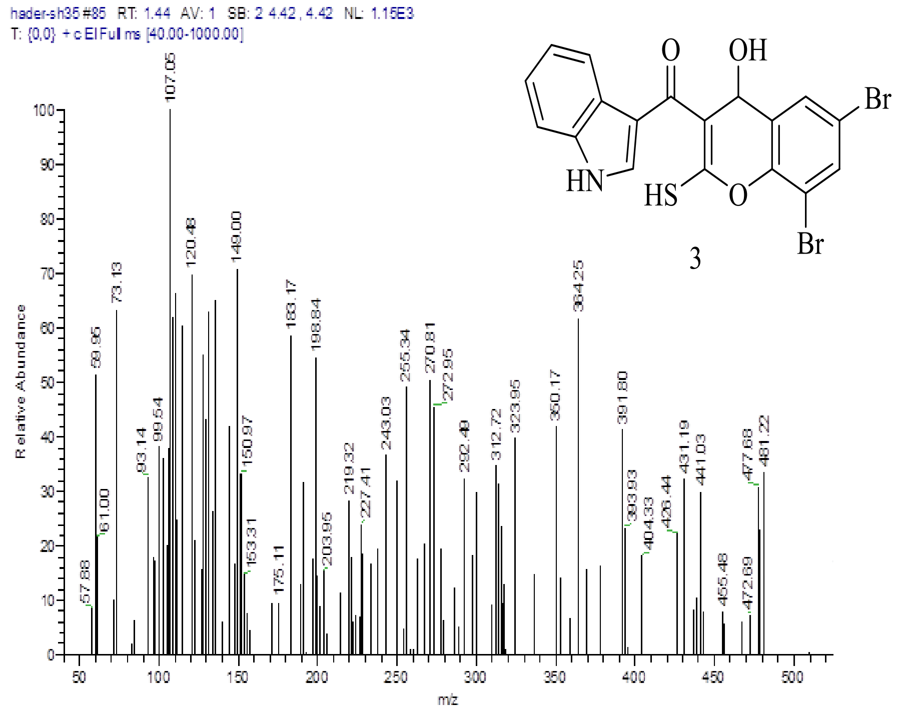

Figure 8: Mass analysis of 3

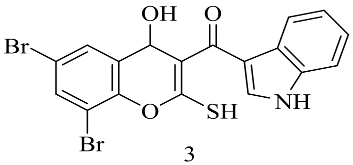

3

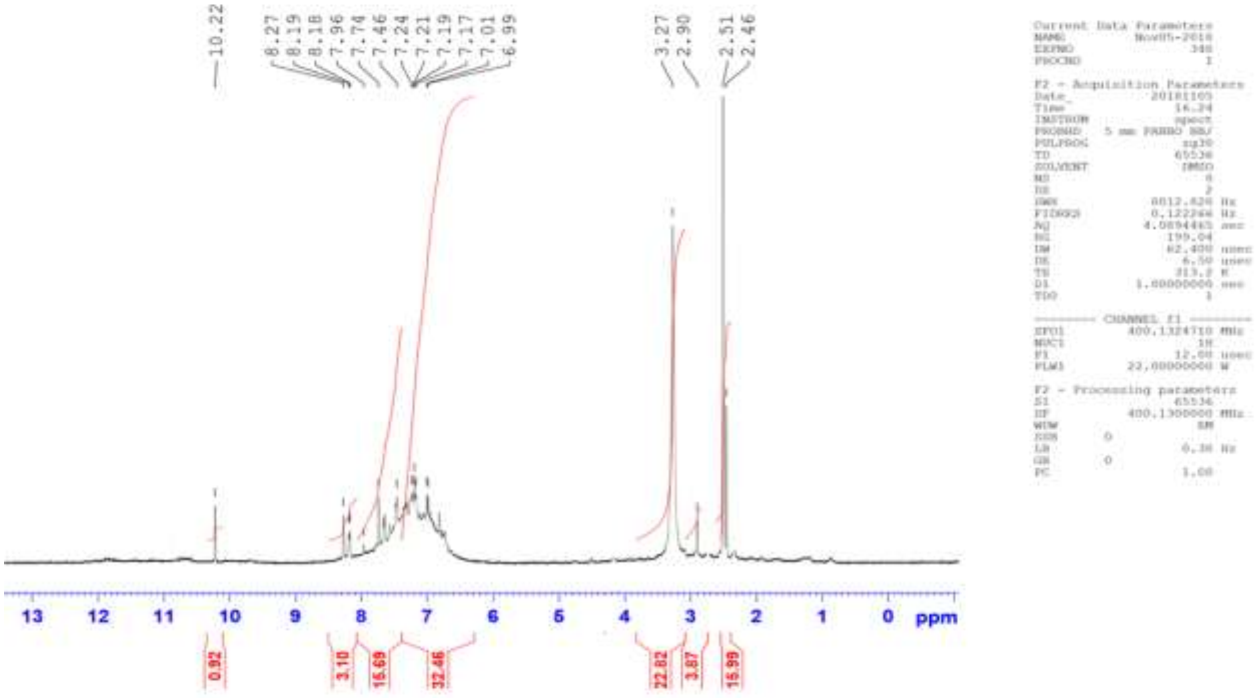

Figure 9: ^{1}HNMR analysis of 3

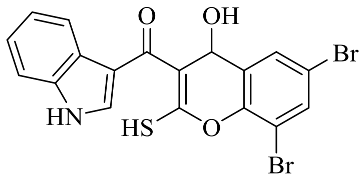

3

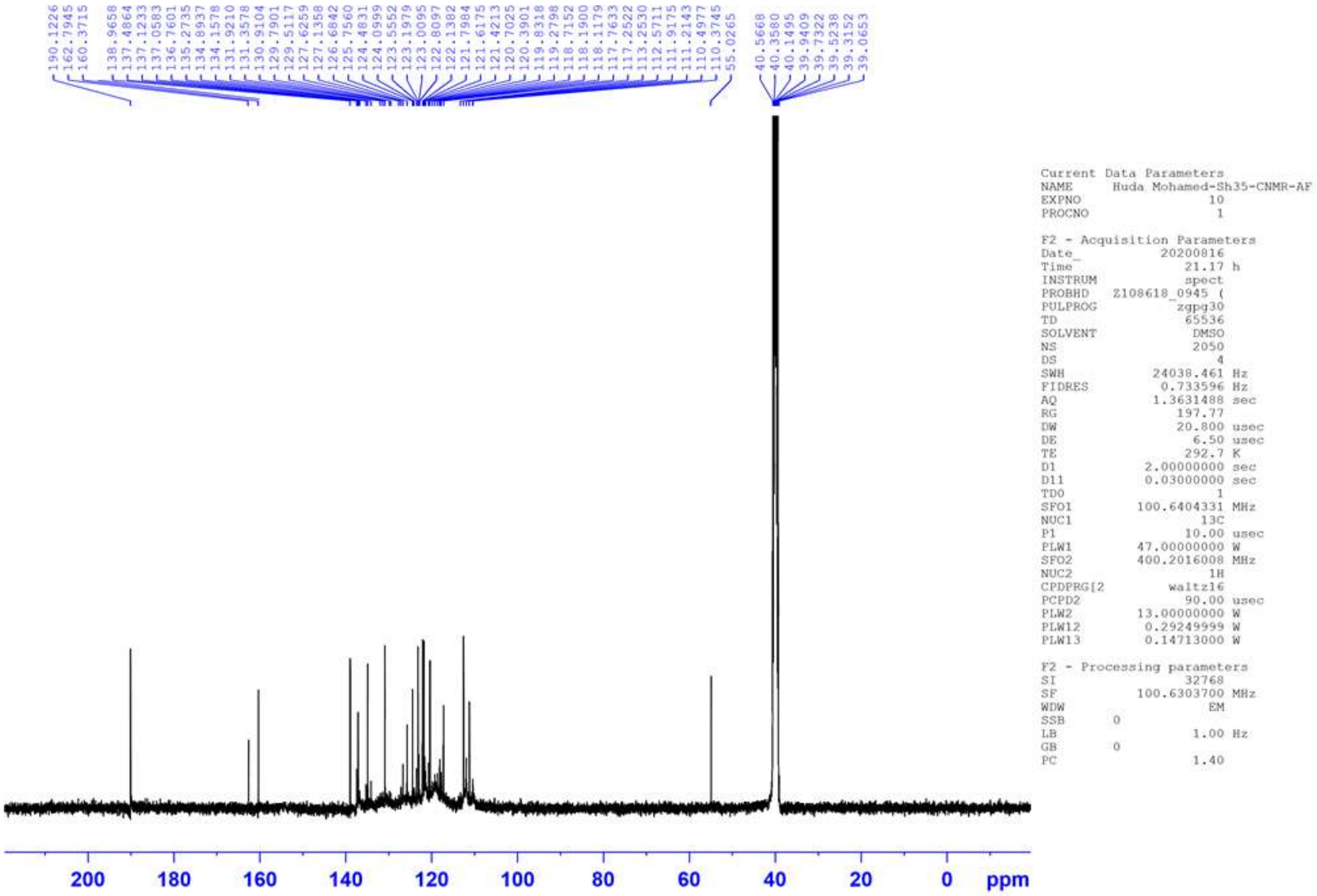

Figure 10: ^{13}CNMR analysis of 3

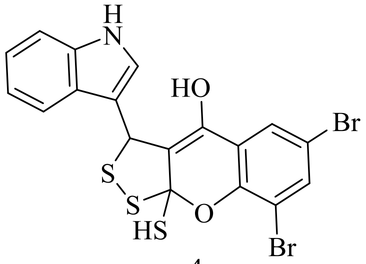

4

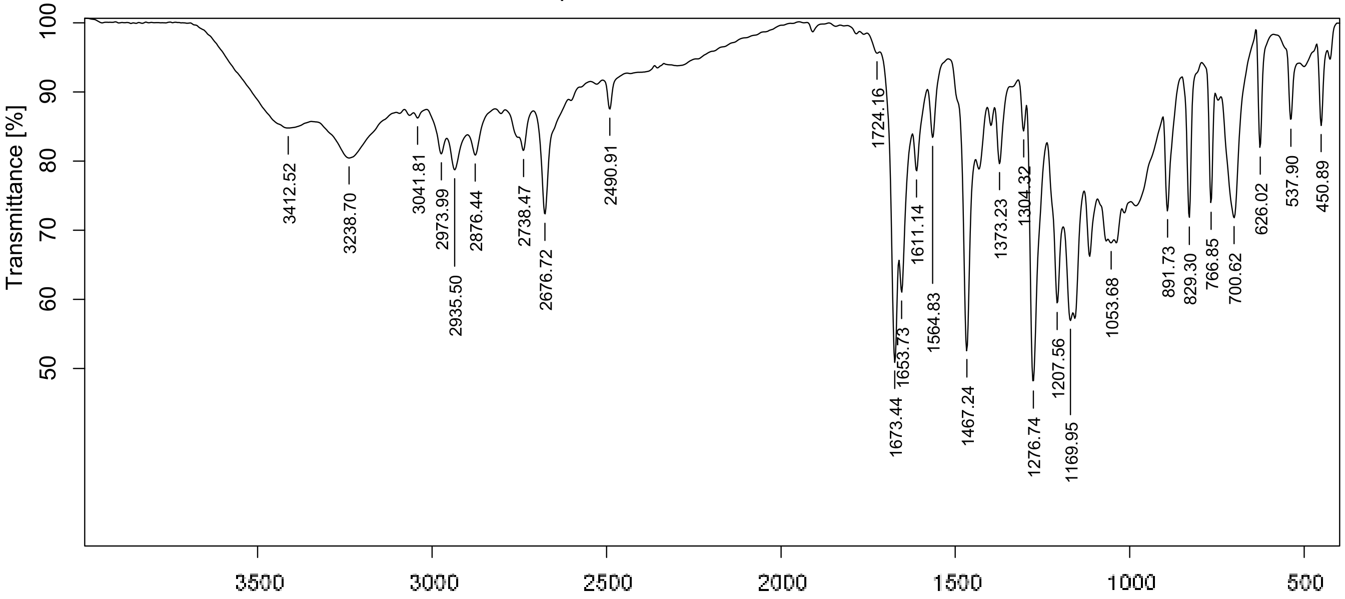

Figure 11: IR analysis of 4

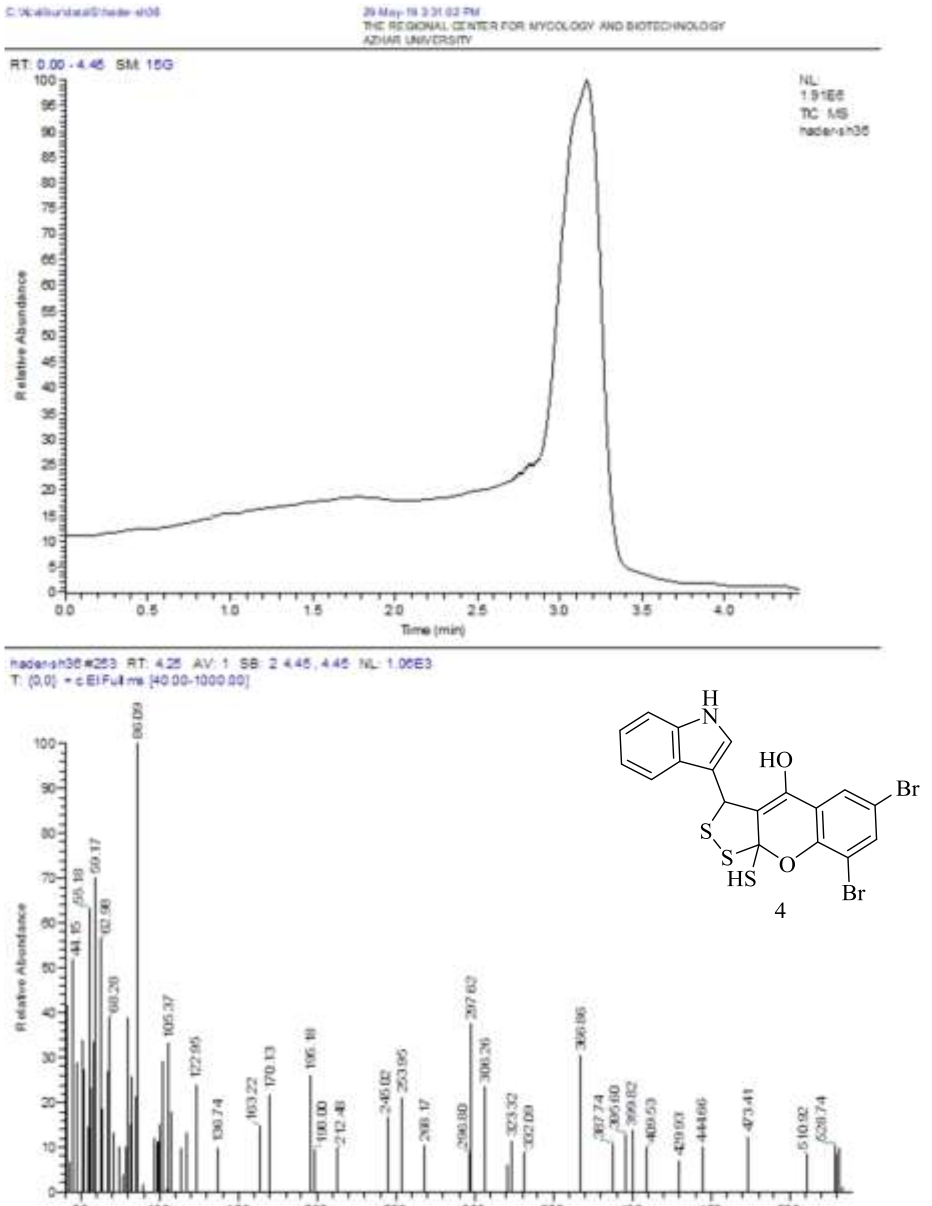

Figure 12 : Mass analysis of 4

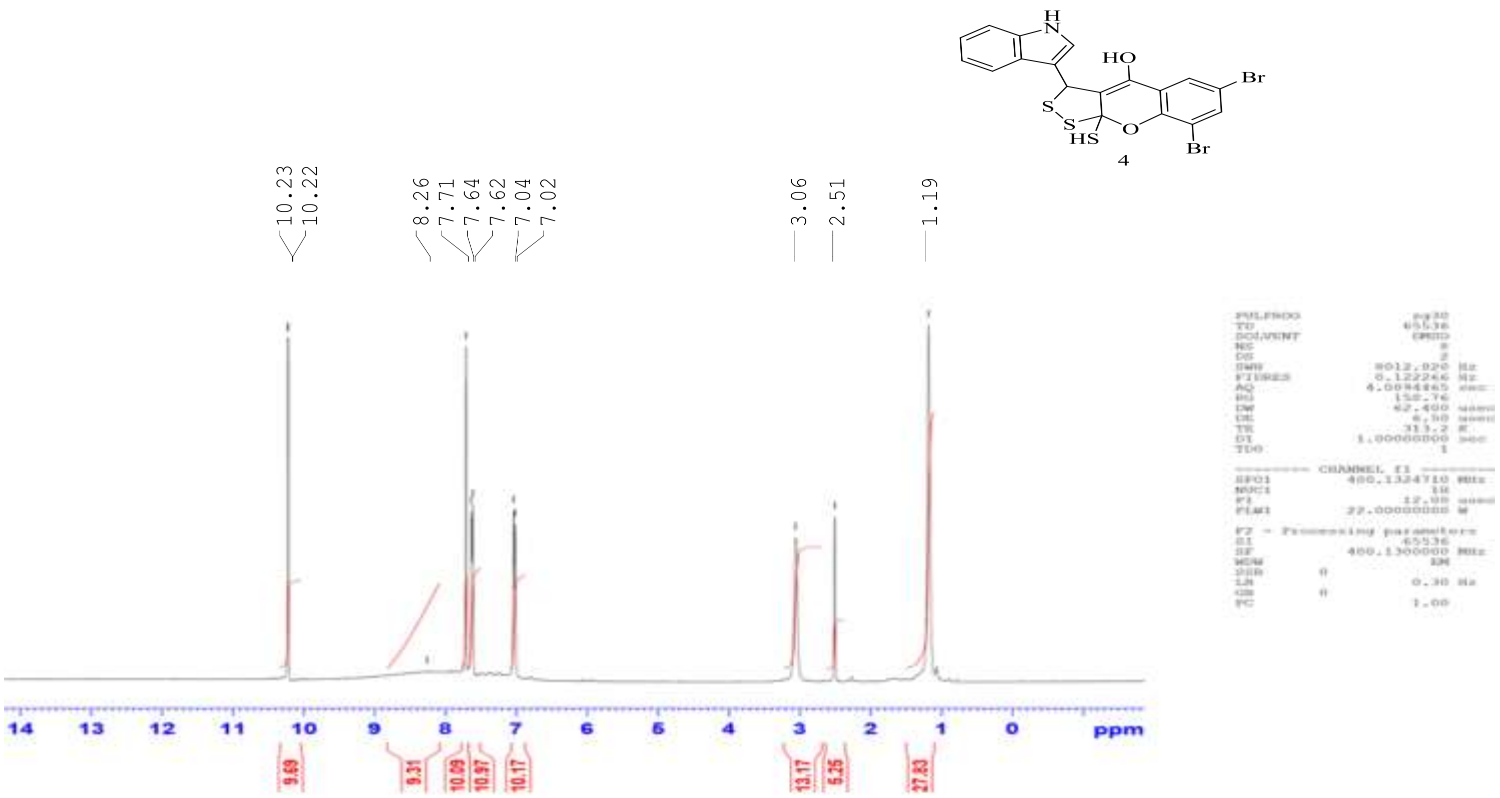

Figure 13 : ^{1}HNMR analysis of 4

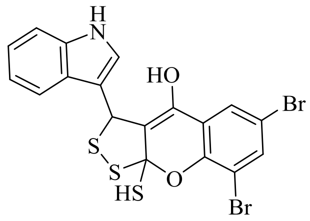

4

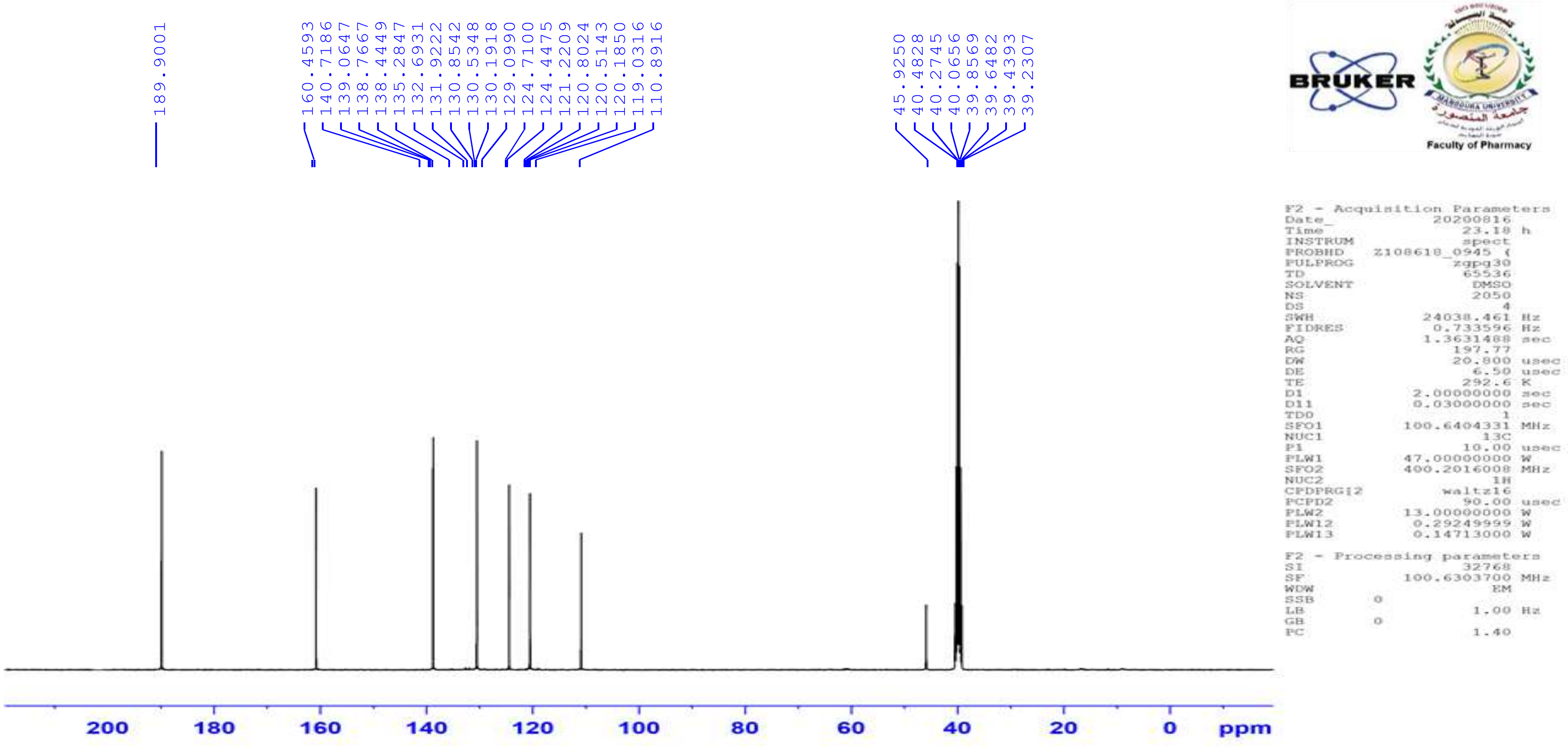

Figure14 : ^{13}CNMR analysis of 4

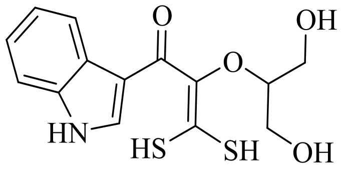

5

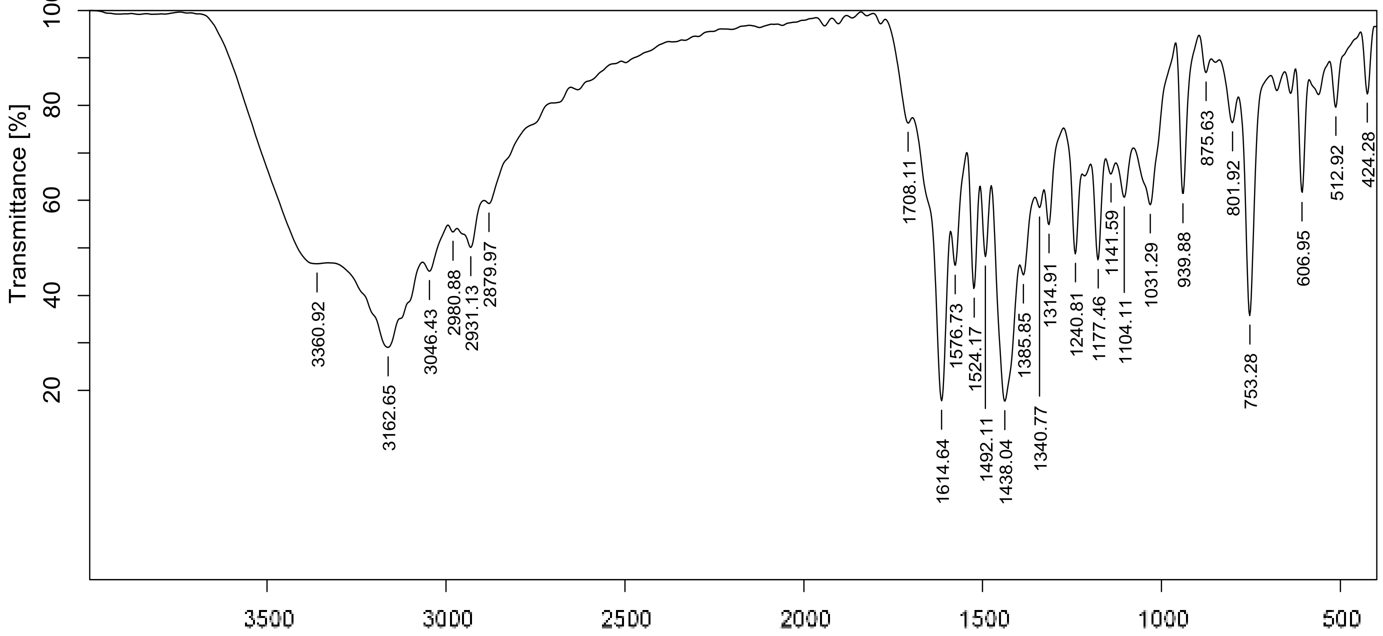

Figure 15 : IR analysis of 5

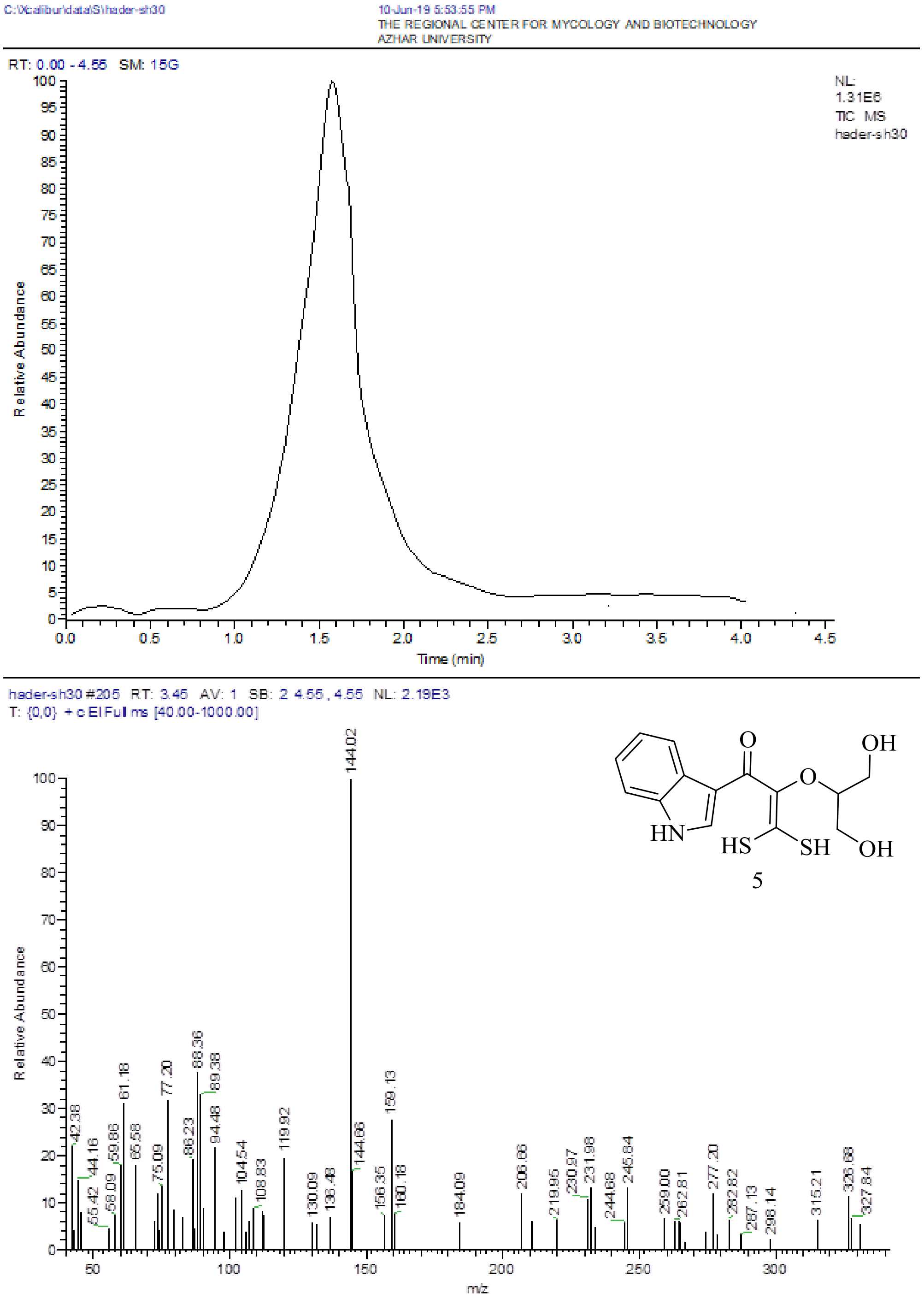

Figure 16 : Mass analysis of 5

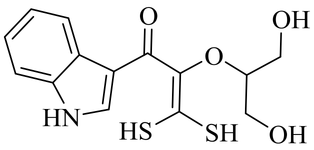

5

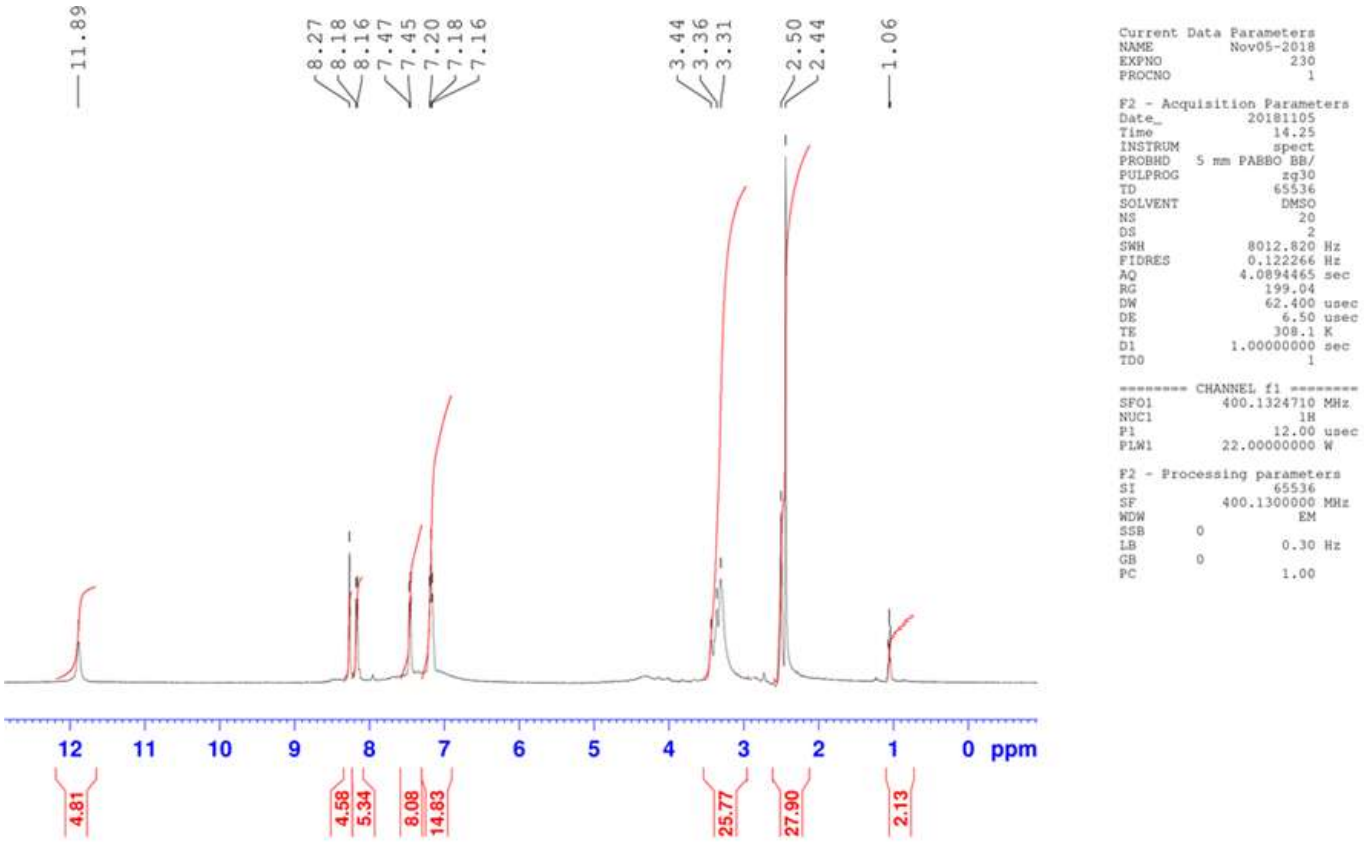

Figure 17: ^{1}HNMR analysis of 5

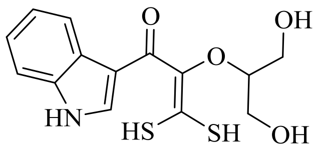

5

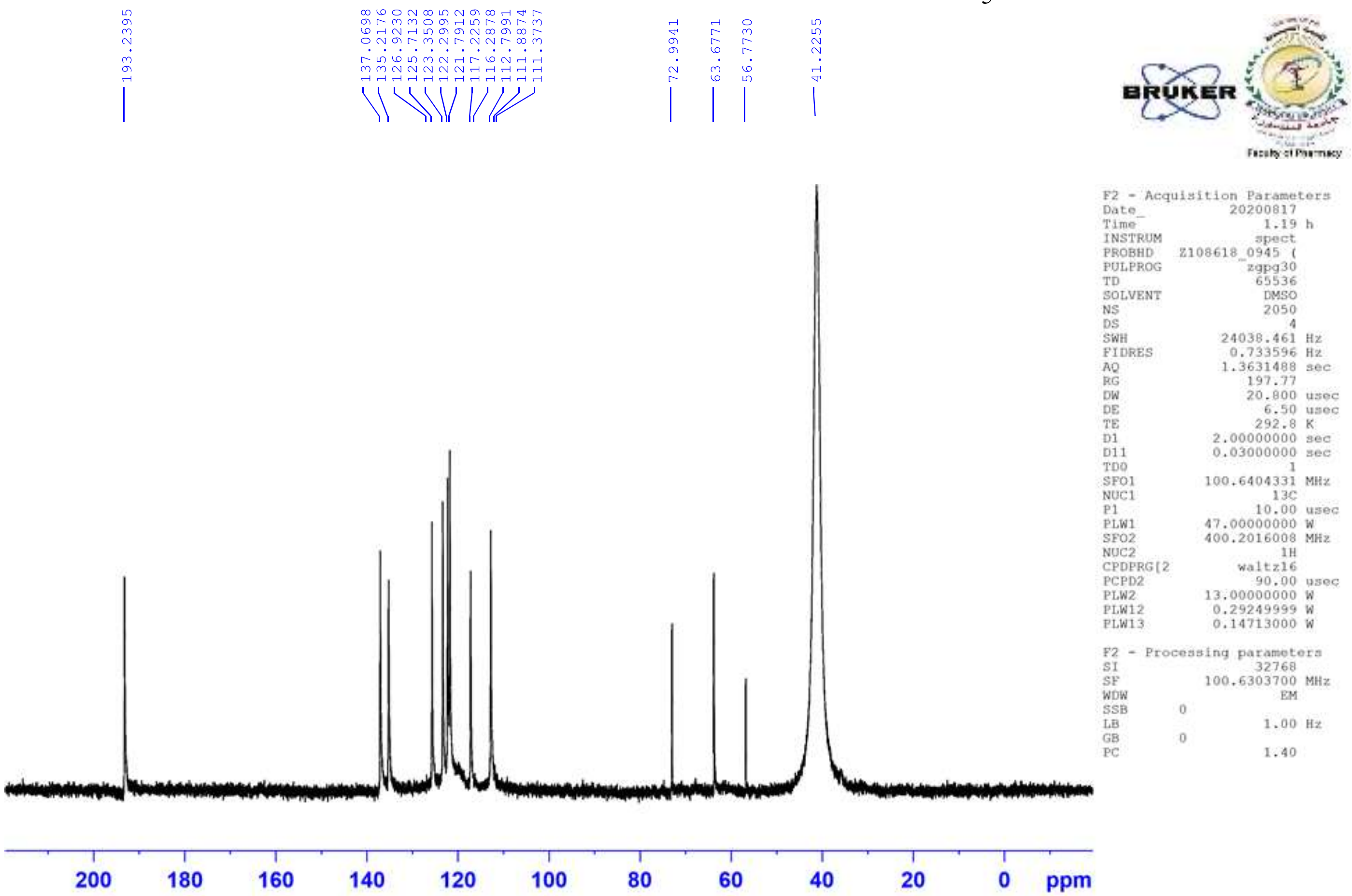

Figure 18: ^{13}CNMR analysis of 5

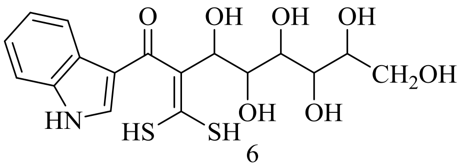

6

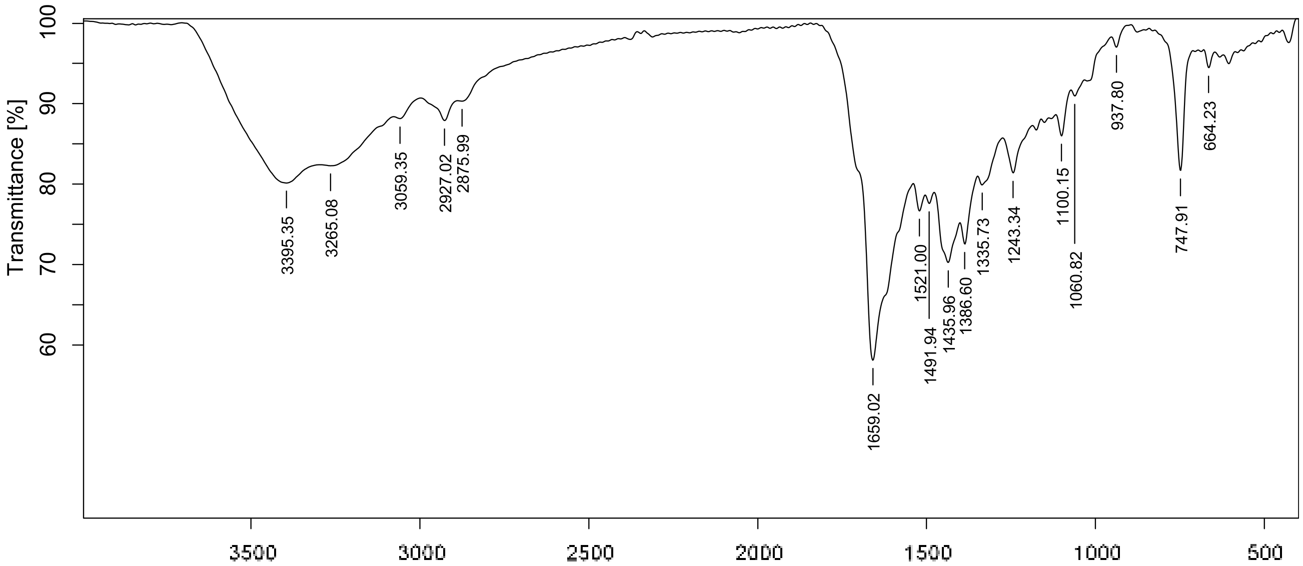

Figure 19: IR analysis of 6

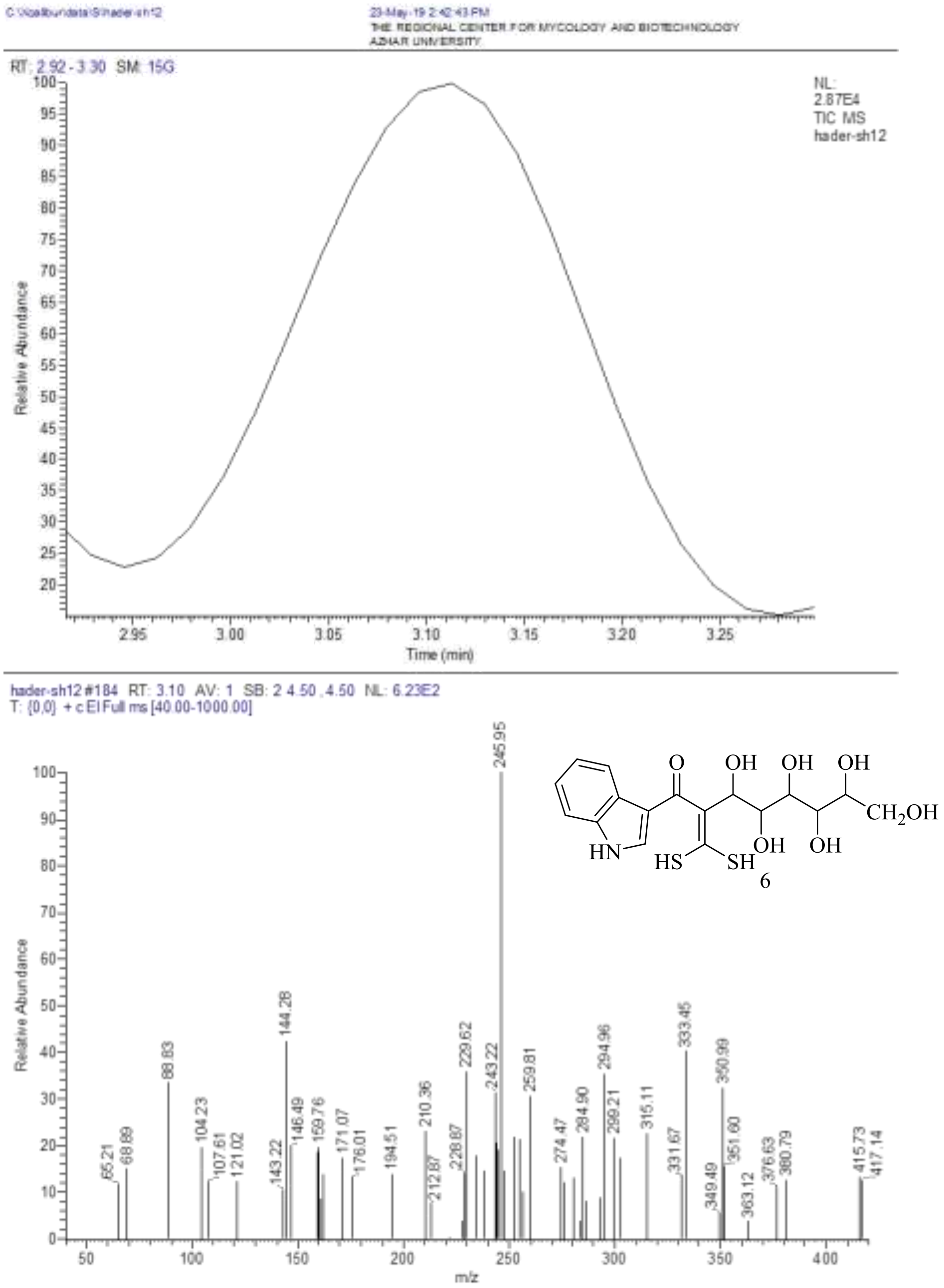

Figure 20 : Mass analysis of 6

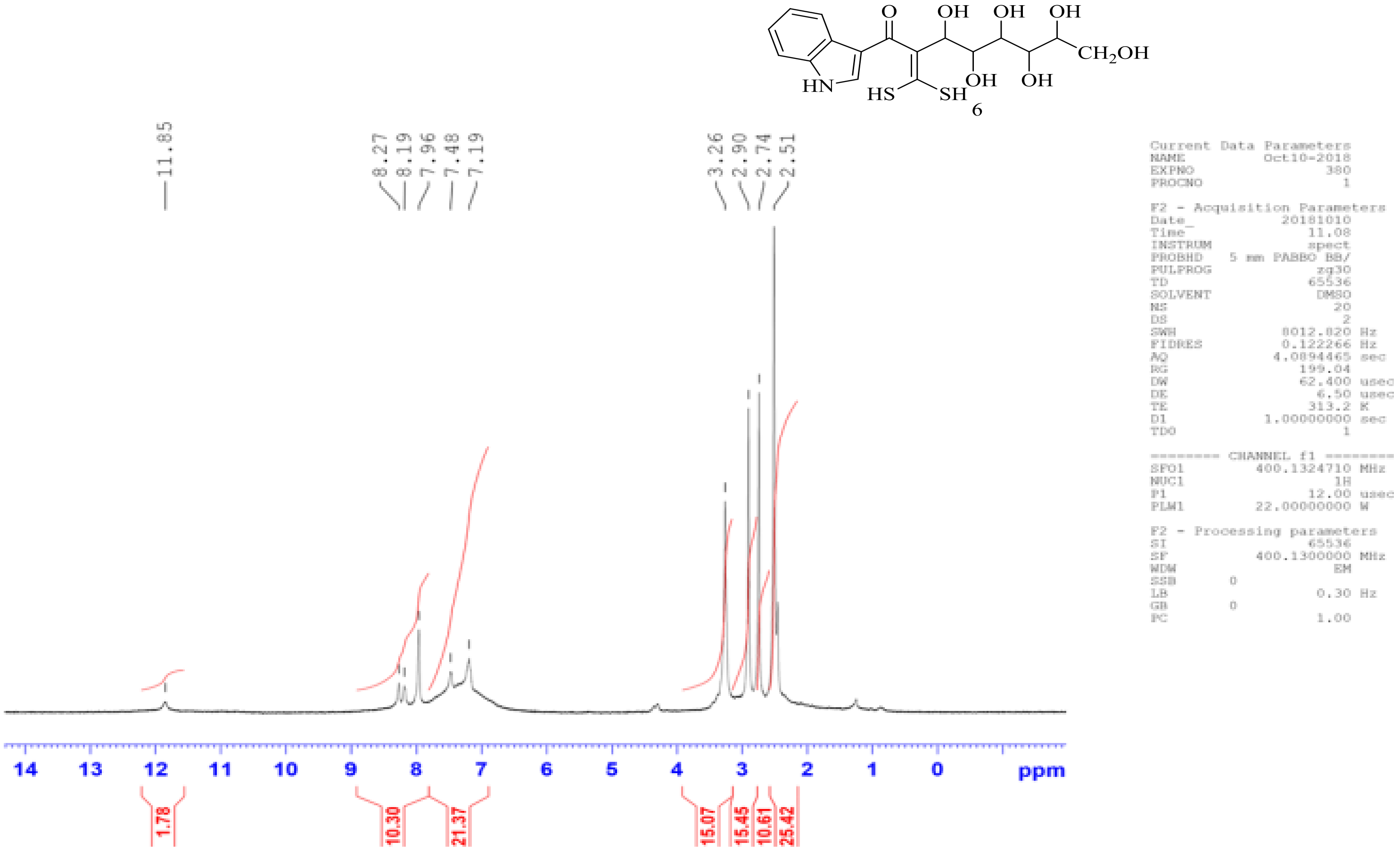

Figure 21 : ^{1}HNMR analysis of 6

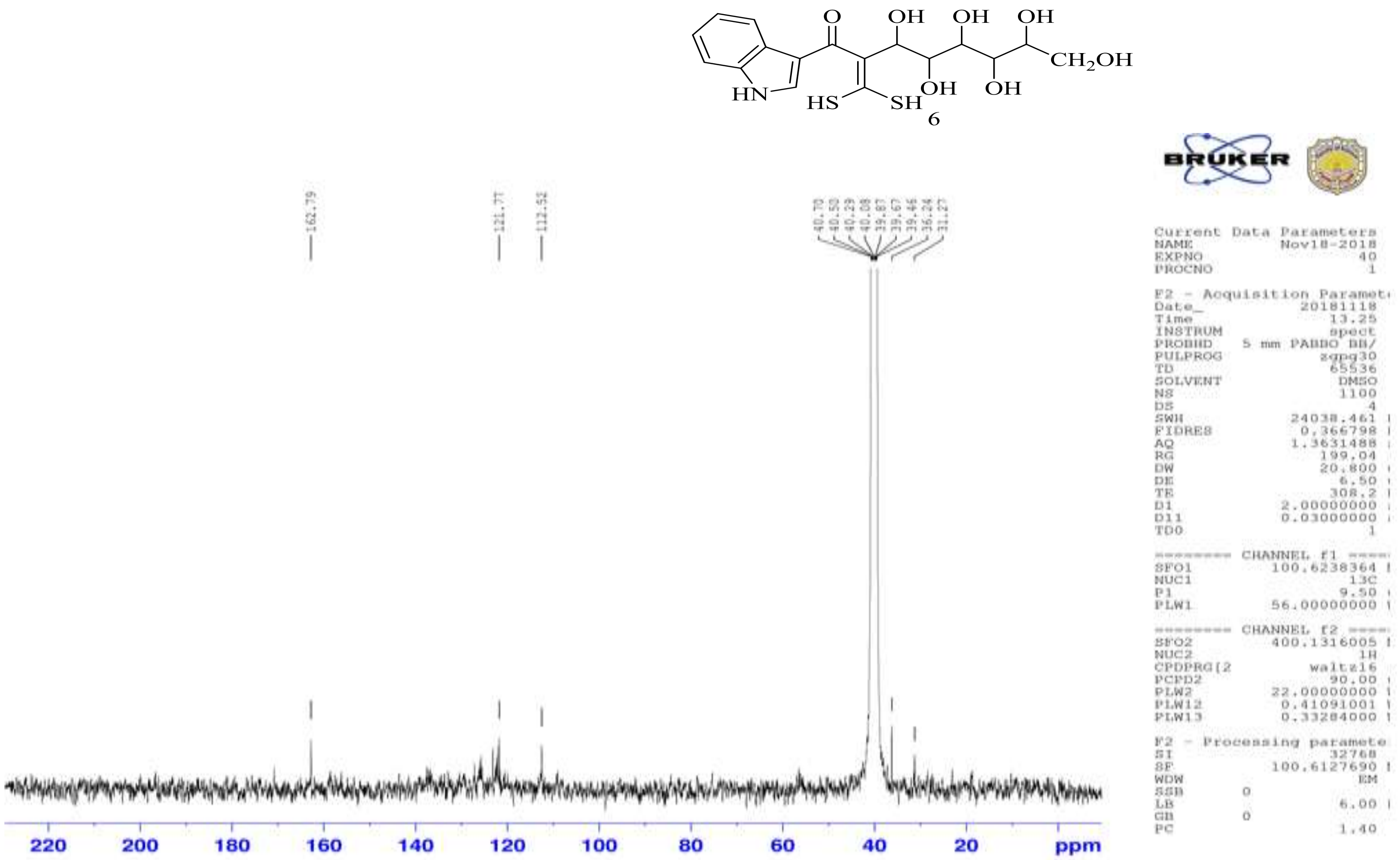

Figure 22 : ^{13}CNMR analysis of 6

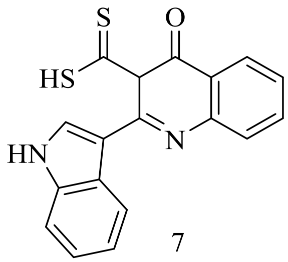

7

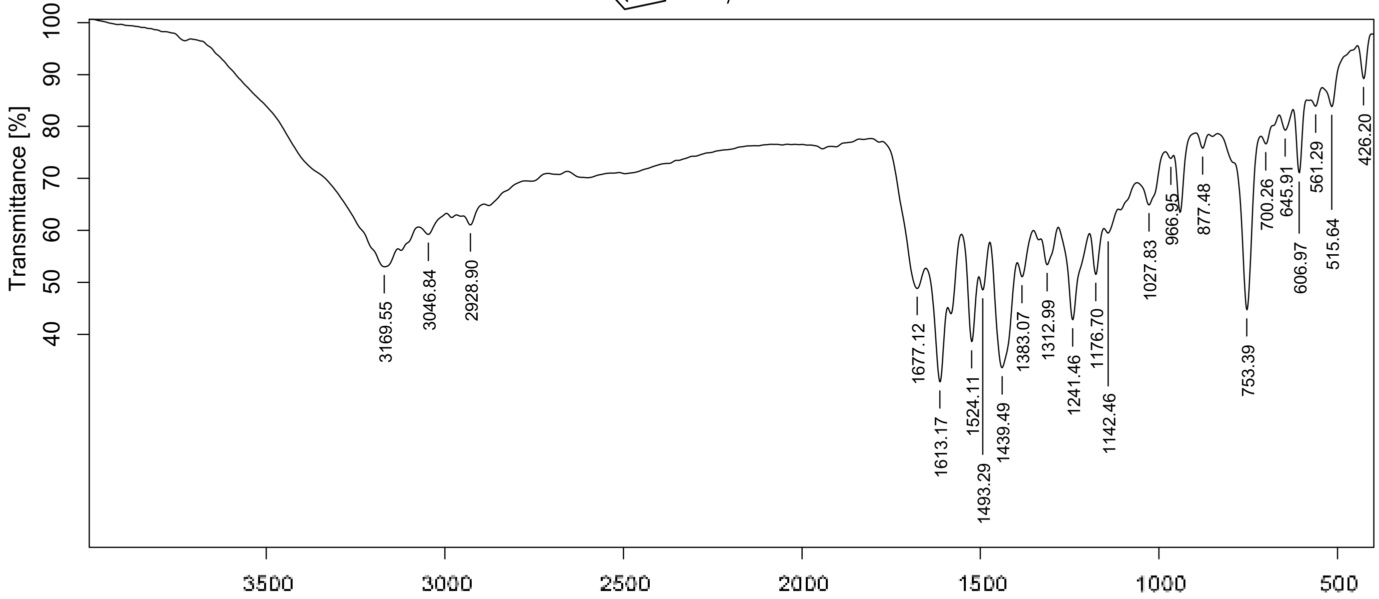

Figure 23 : IR analysis of 7

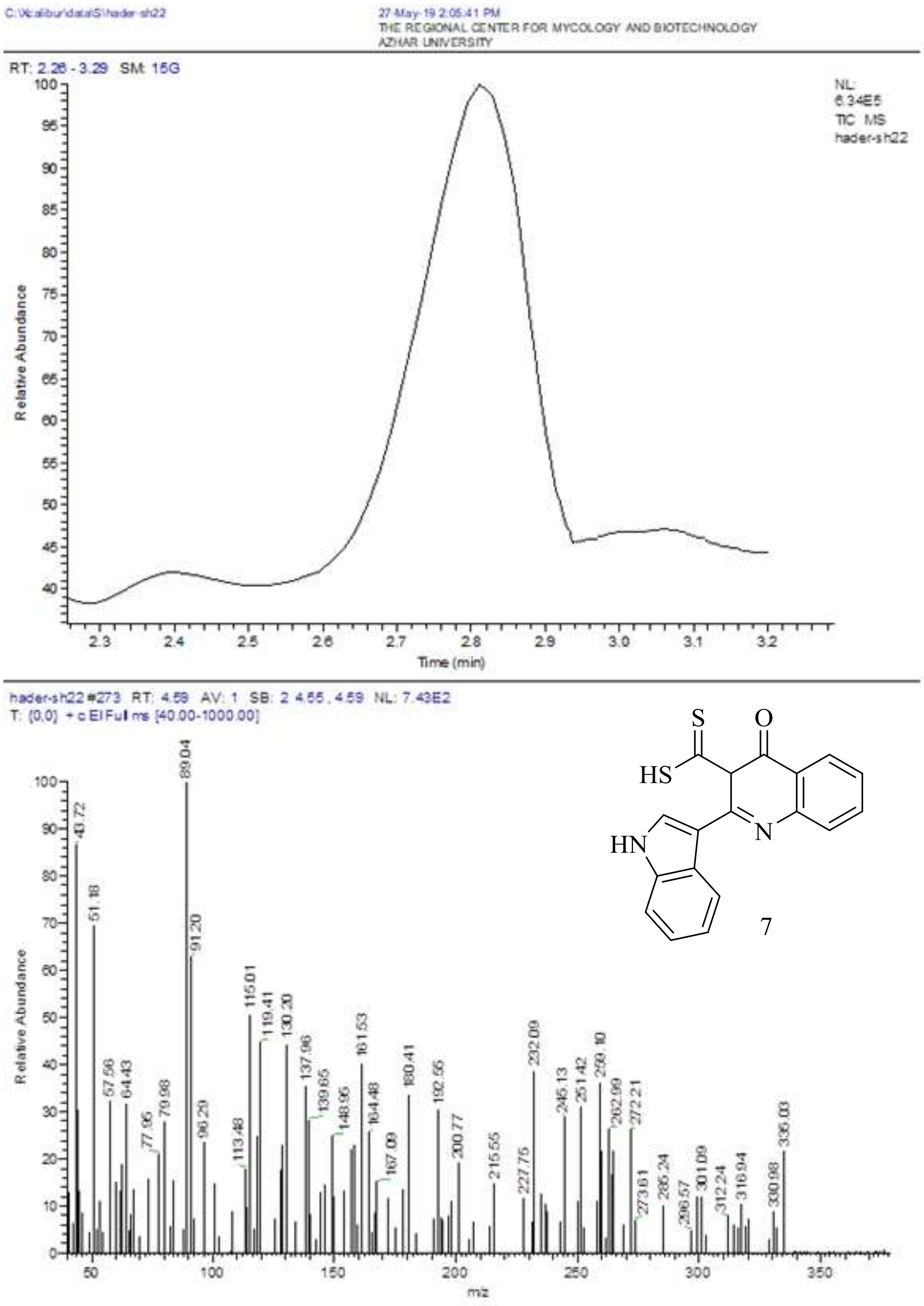

Figure 24: Mass analysis of 7

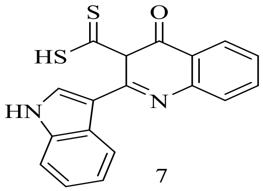

7

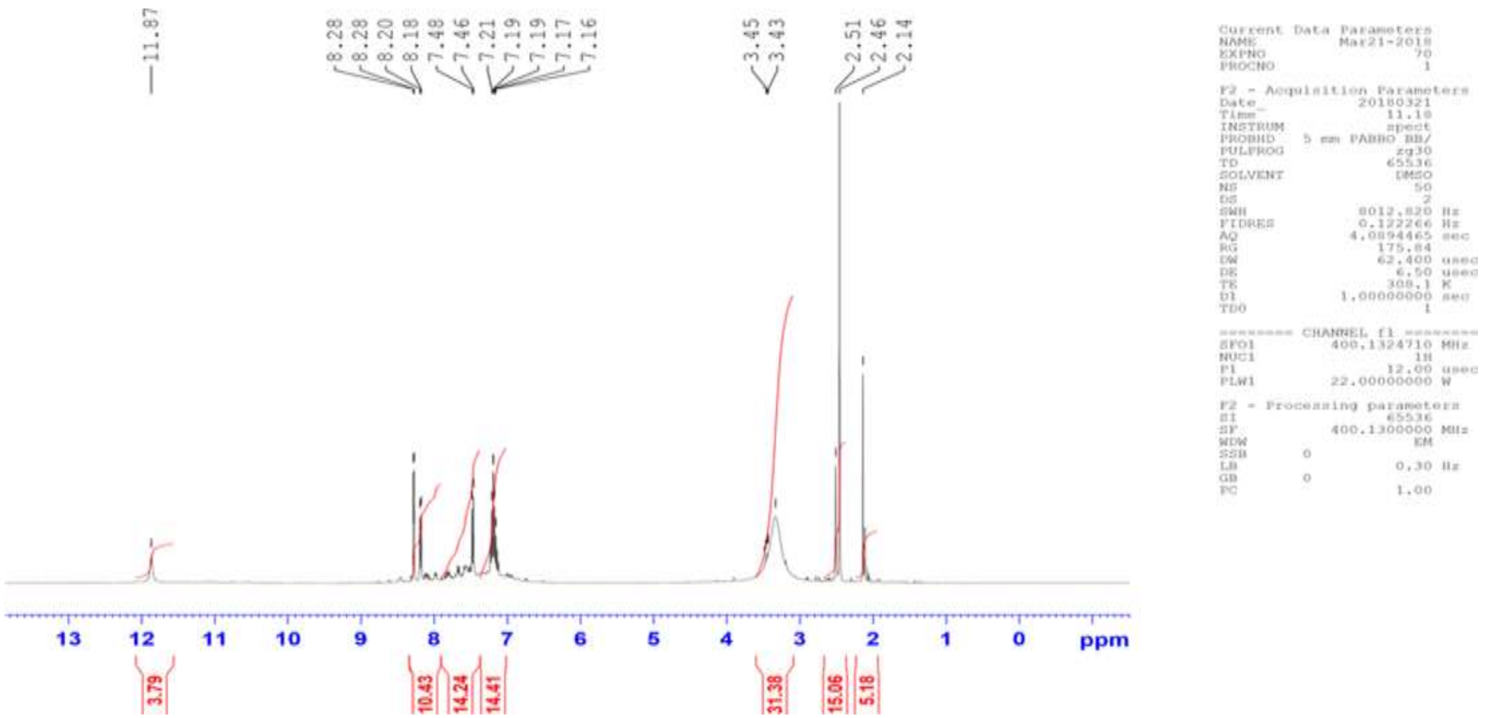

Figure 25 : ^{1}HNMR analysis of 7

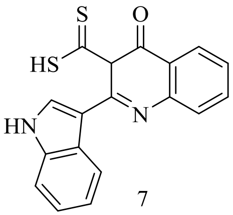

7

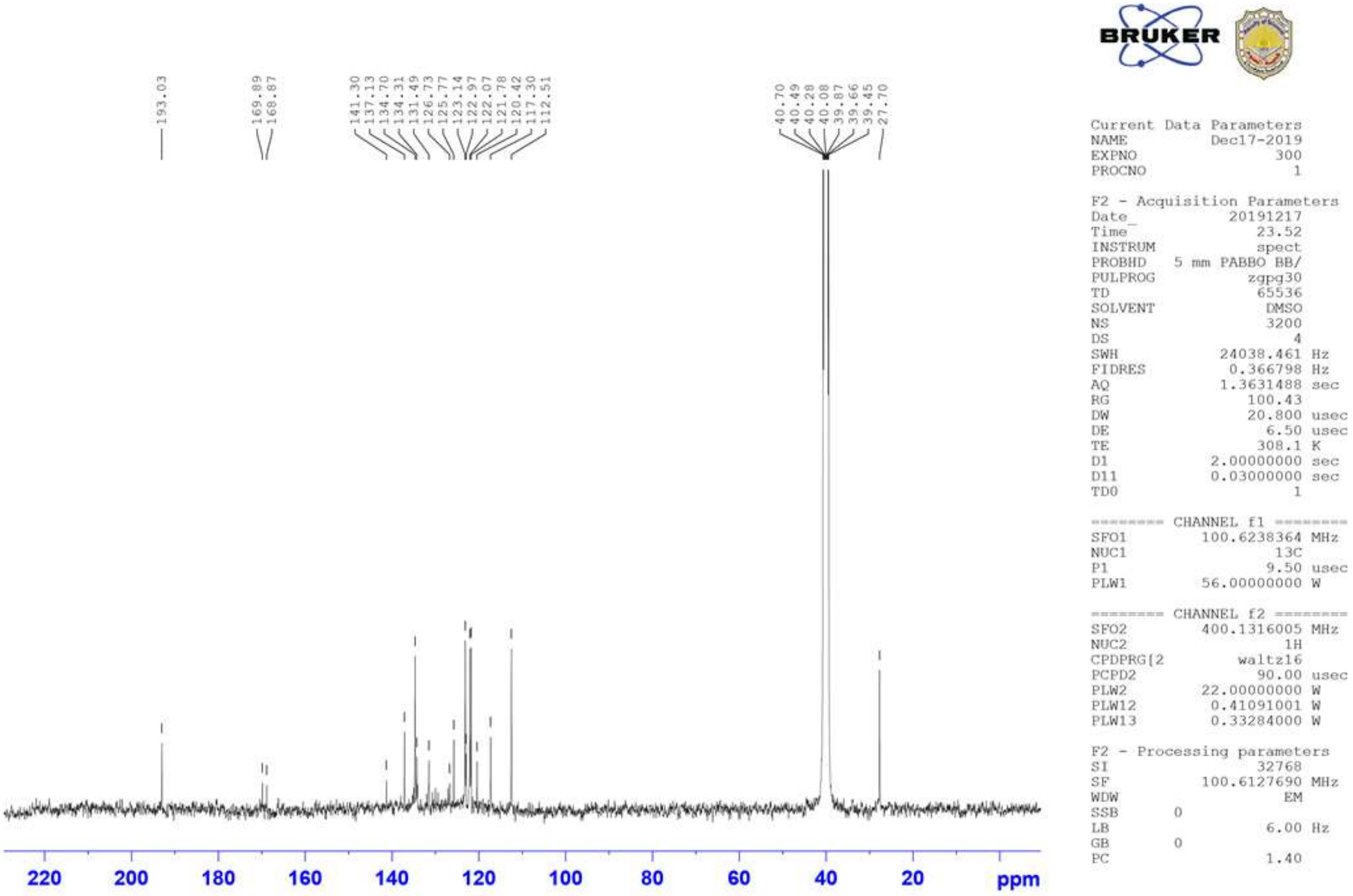

Figure 26 : ^{13}CNMR analysis of 7

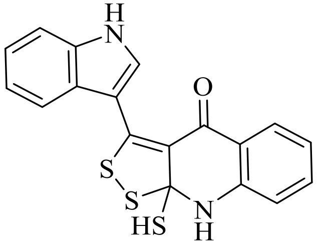

8

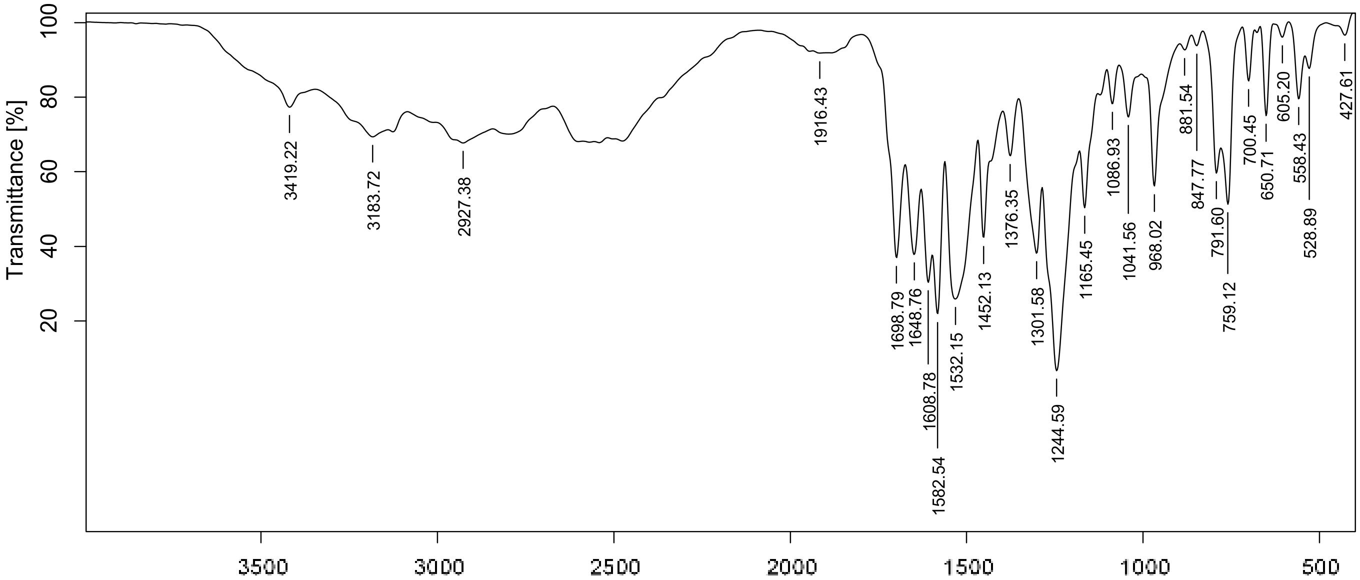

Figure 27 : IR analysis of 8

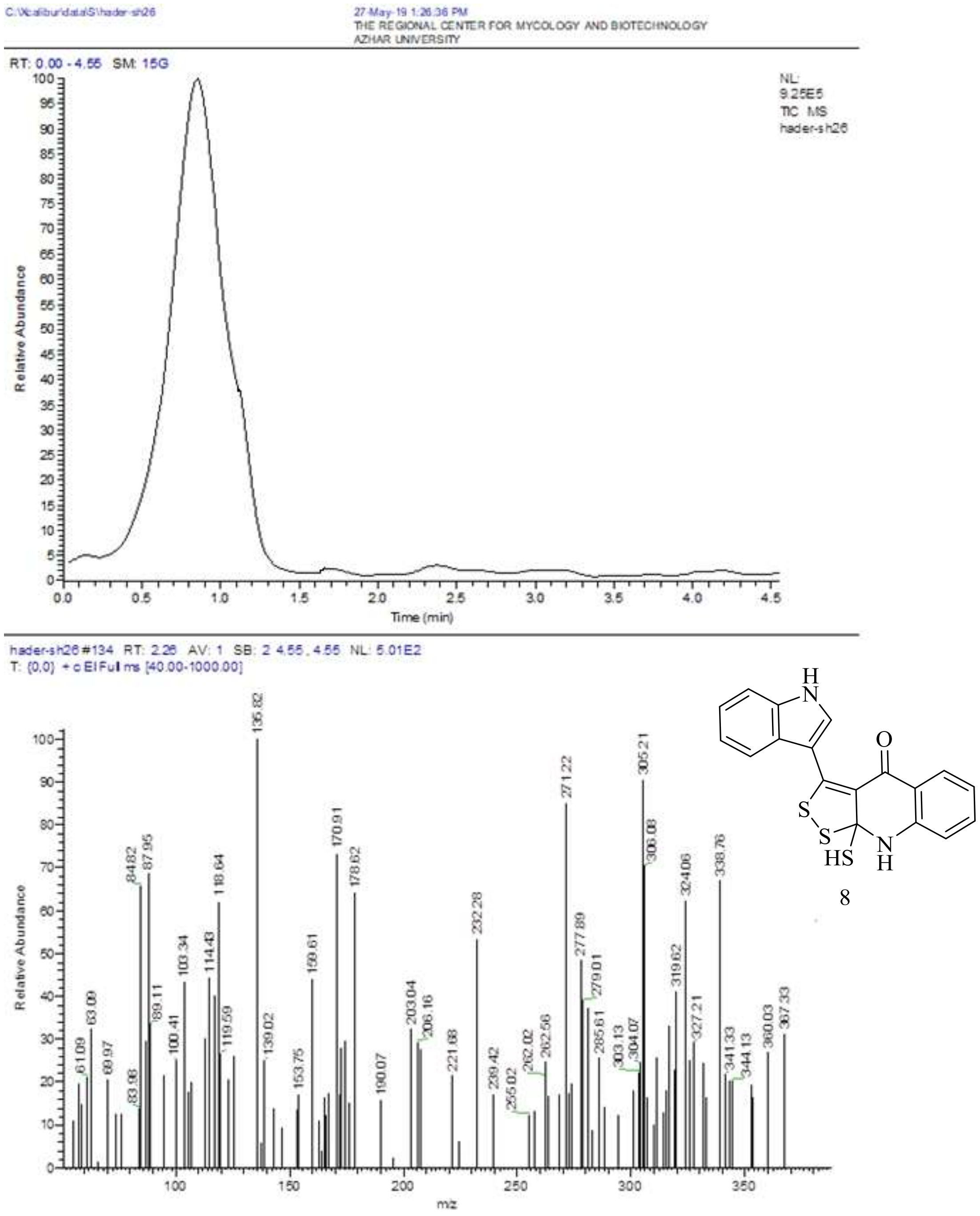

8

Figure 28 : Mass analysis of 8

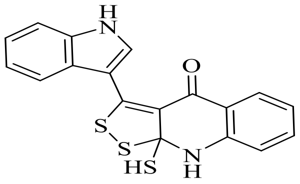

8

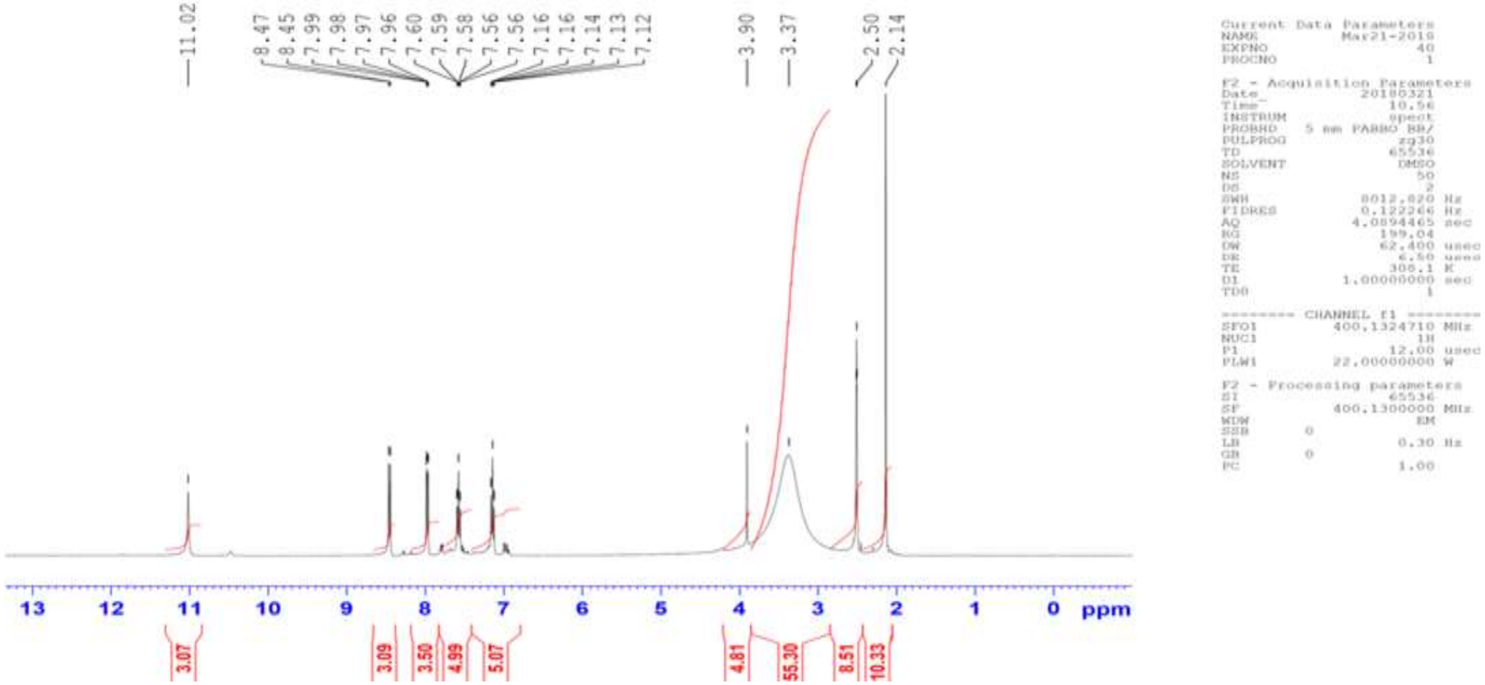

Figure 29 : ^{1}HNMR analysis of 8

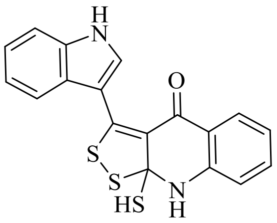

8

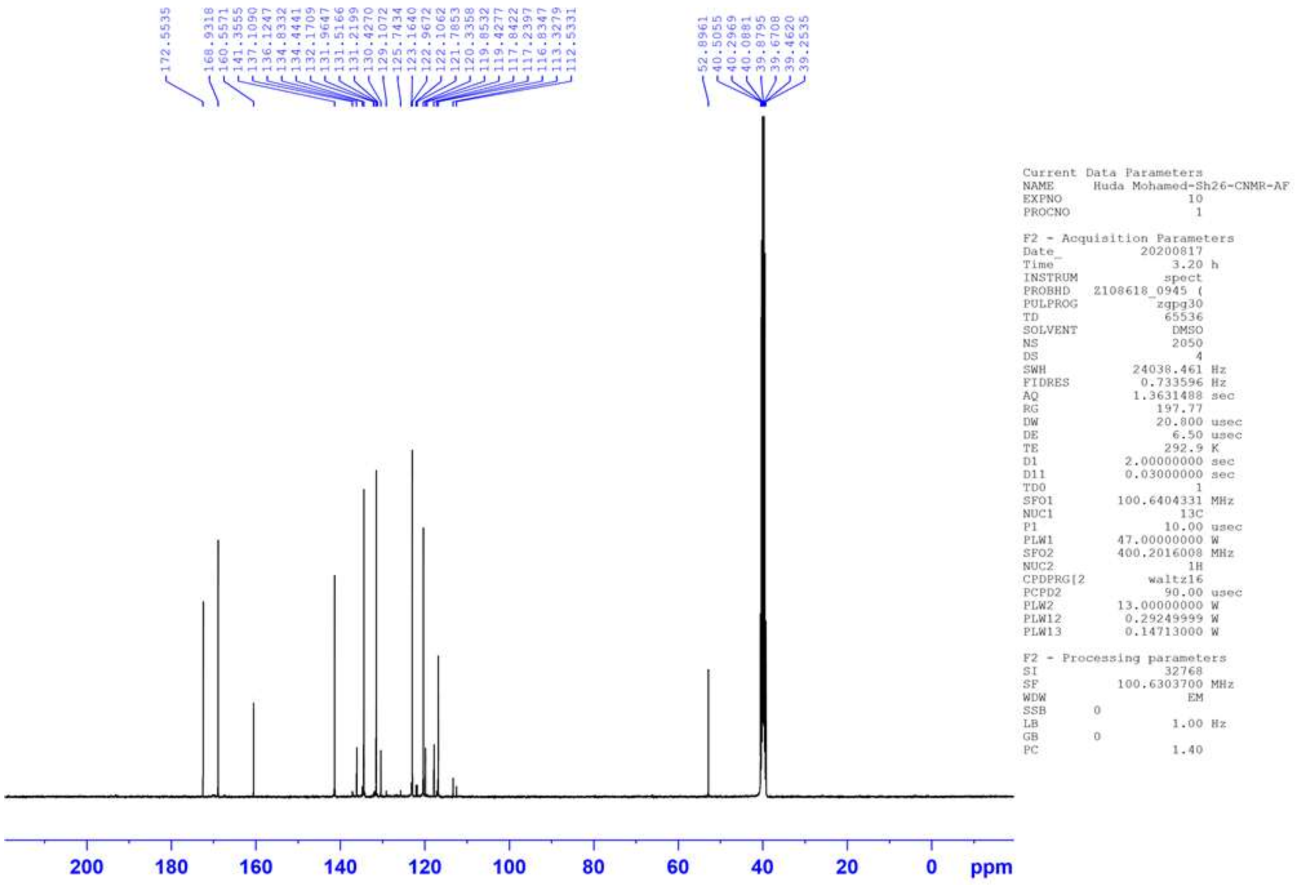

Figure 30 : ^{13}CNMR analysis of 8

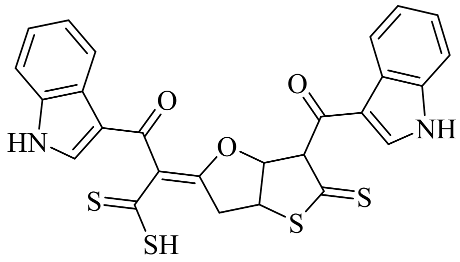

9

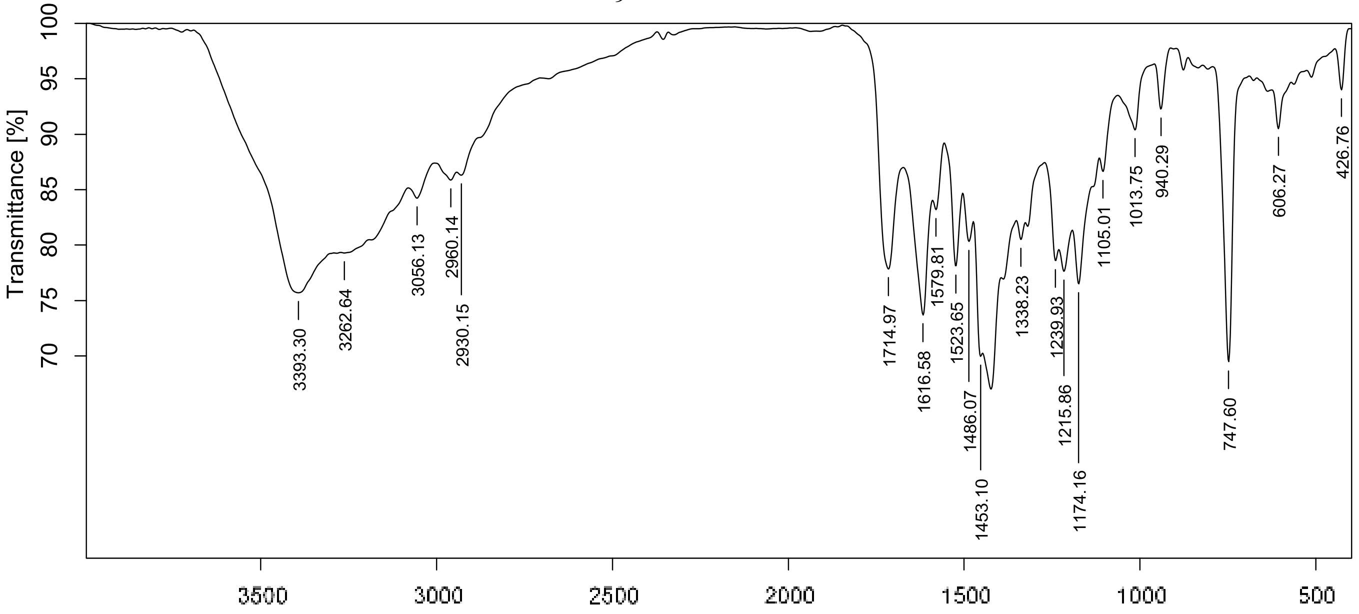

Figure 31 : IR analysis of 9

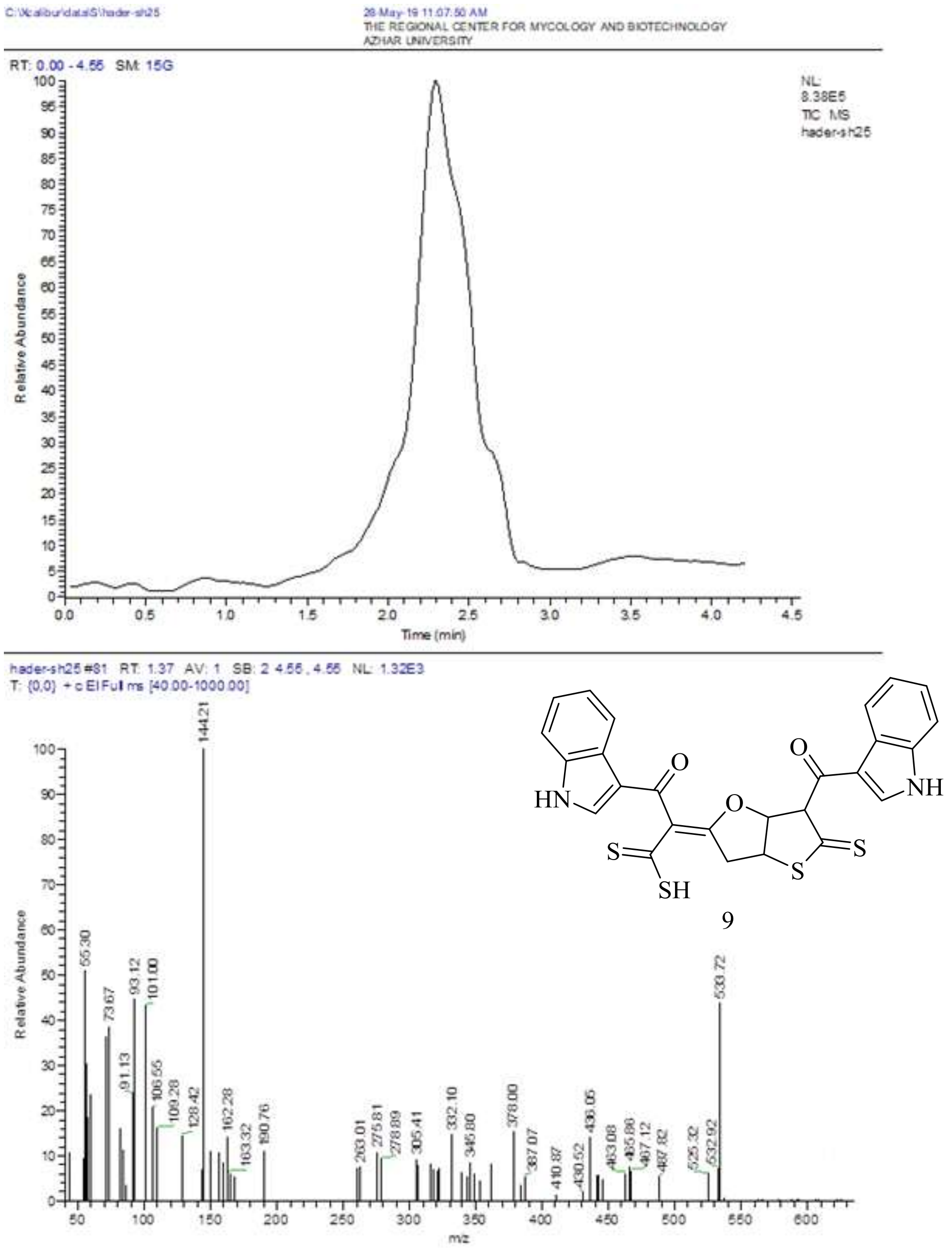

Figure 32 : Mass analysis of 9

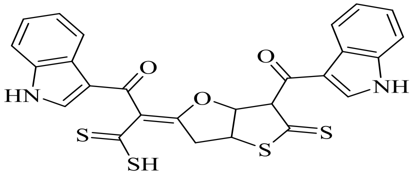

9

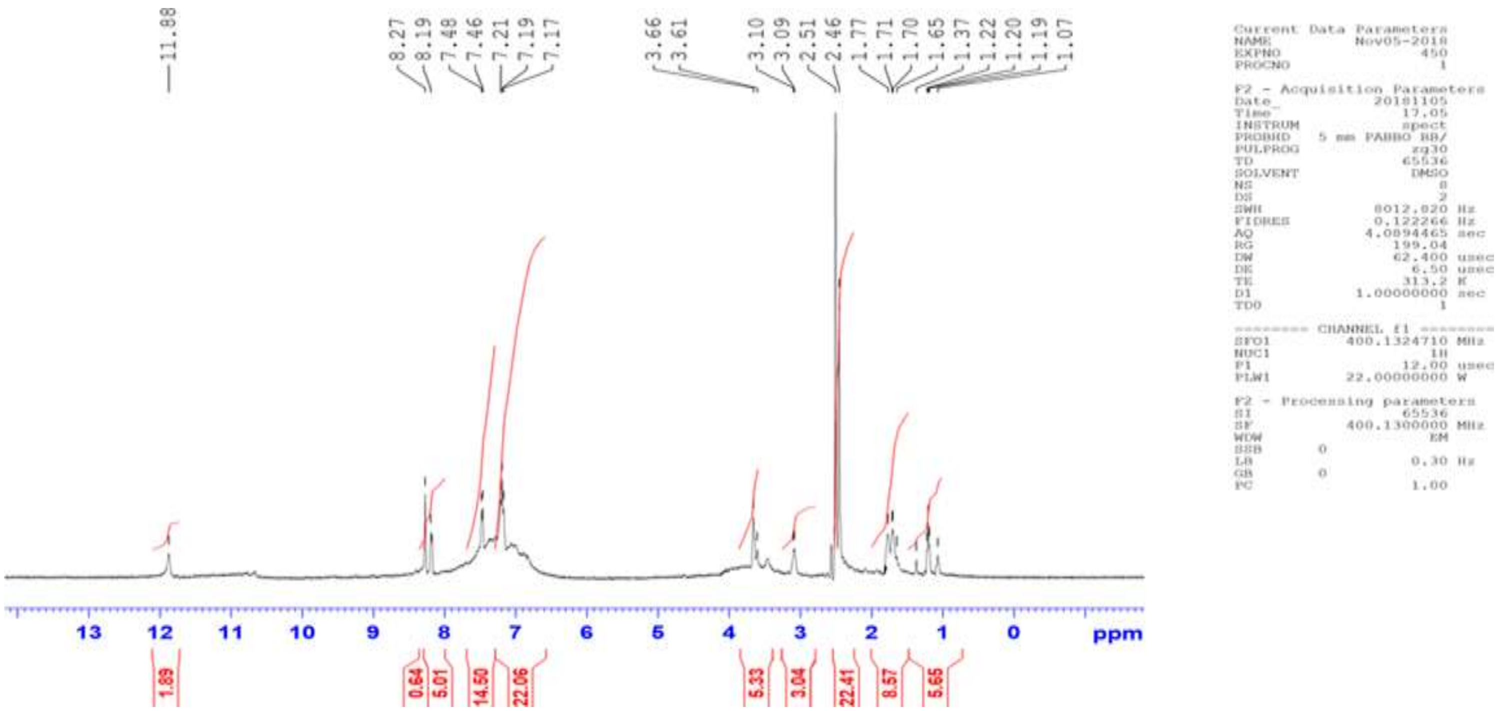

Figure 33: ^{1}HNMR analysis of 9

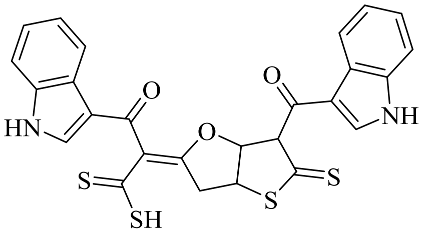

9

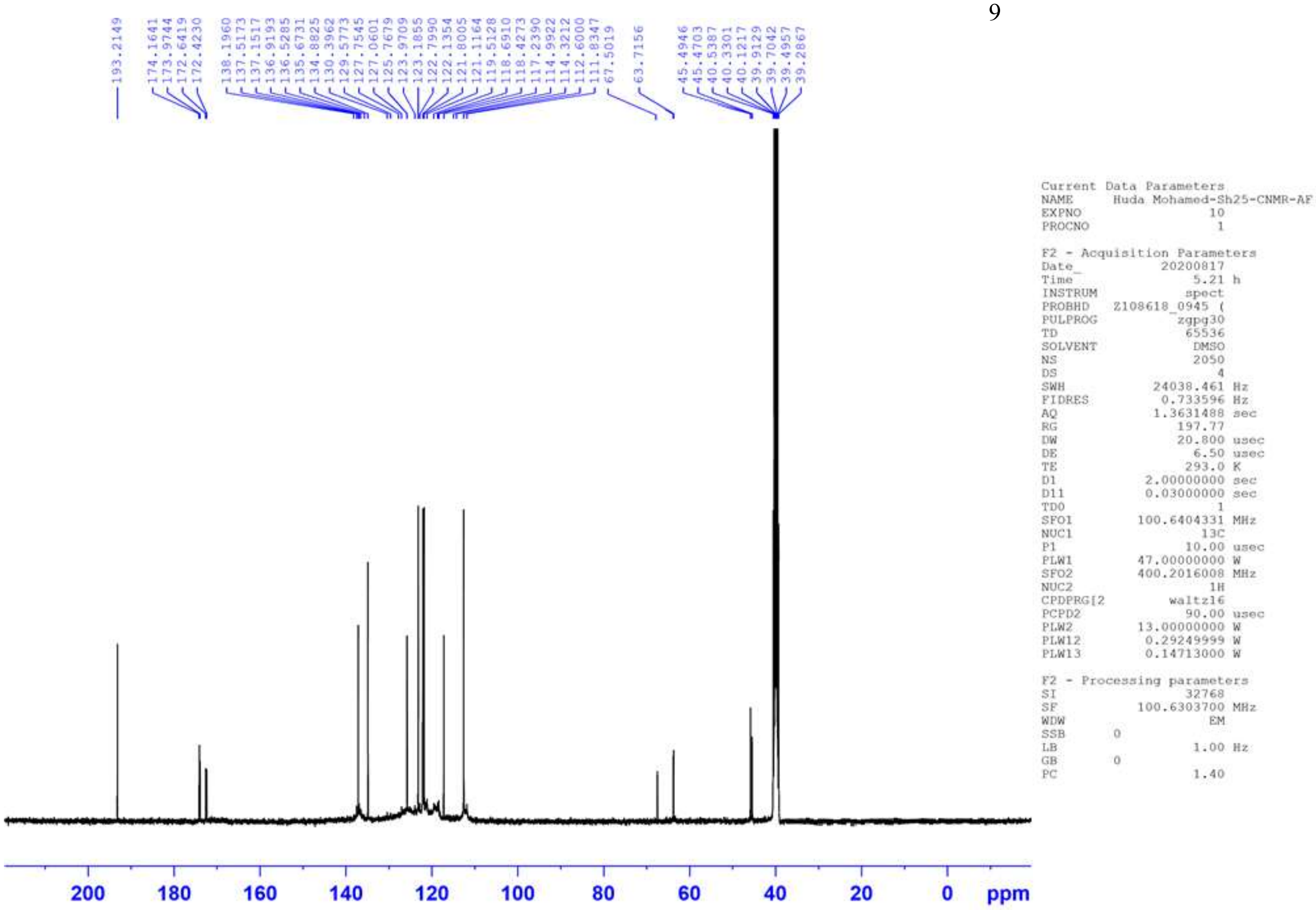

Figure 34 : ^{13}CNMR analysis of 9

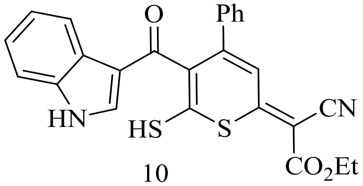

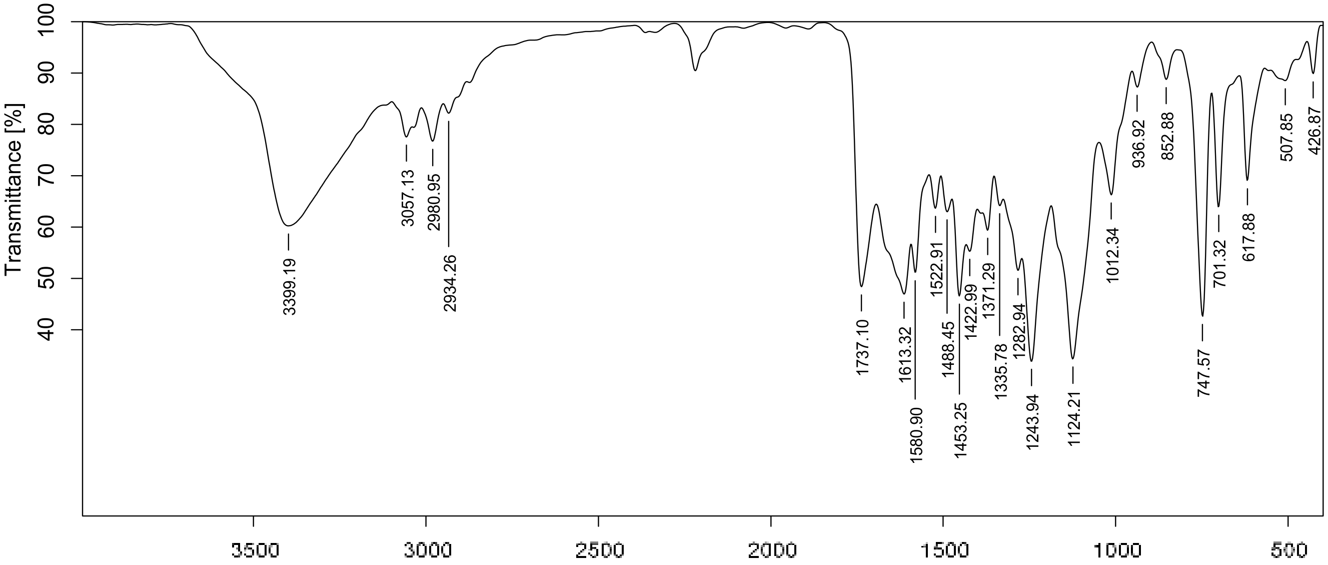

Figure 35 : IR analysis of 10

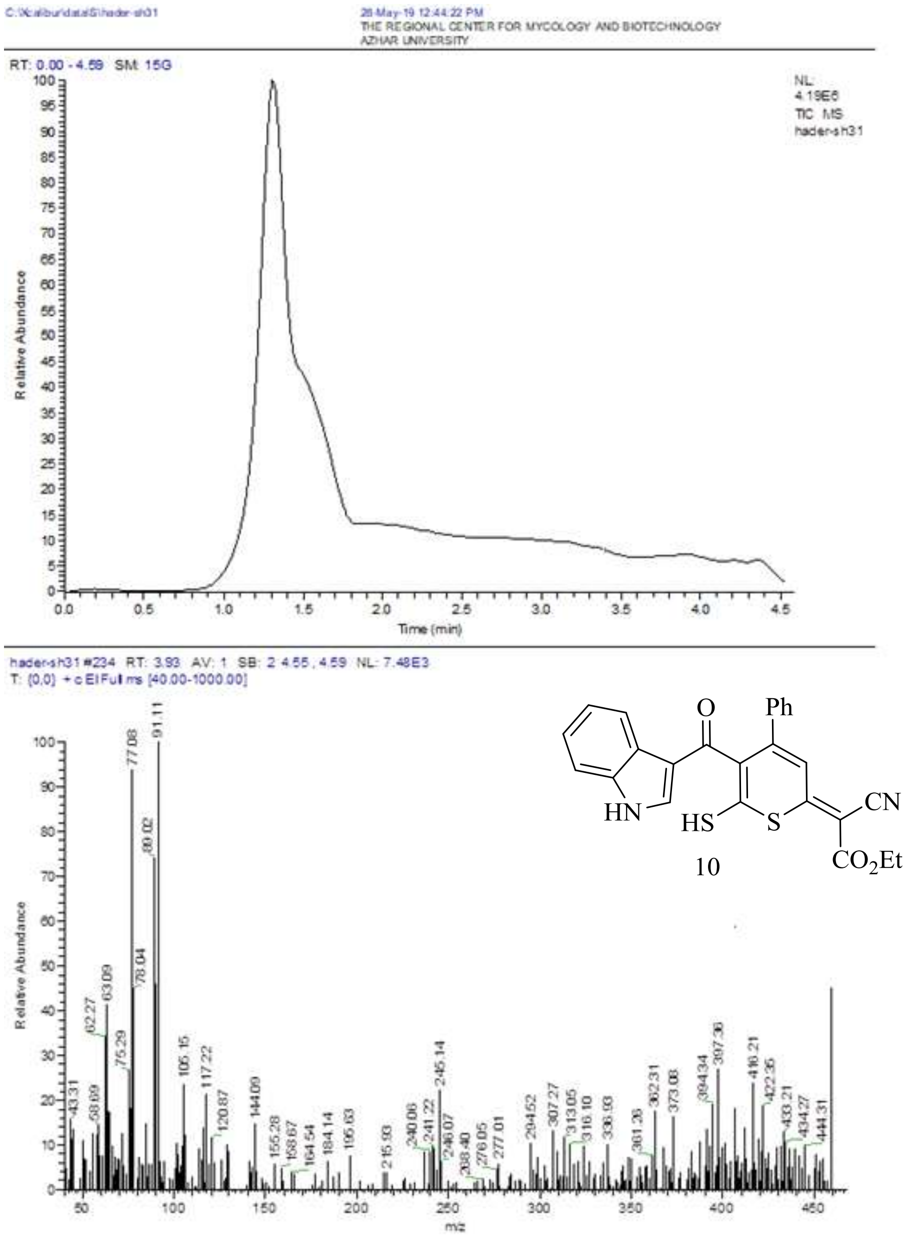

Figure 36 : Mass analysis of 10

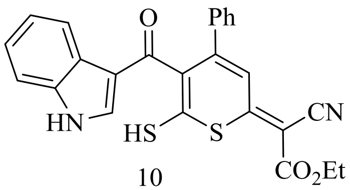

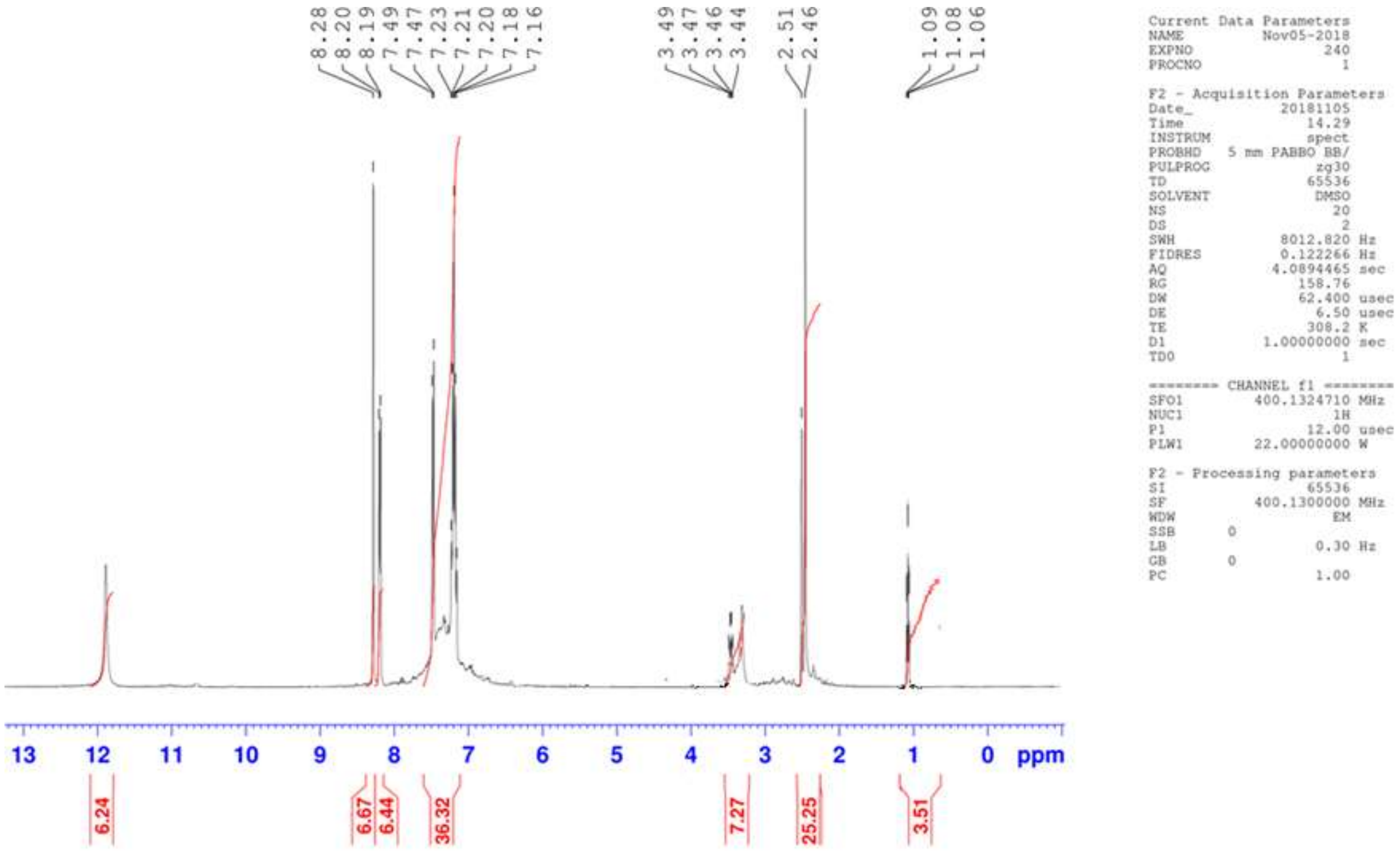

Figure 37 : ^{1}HNMR analysis of 10

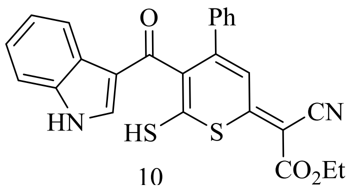

10

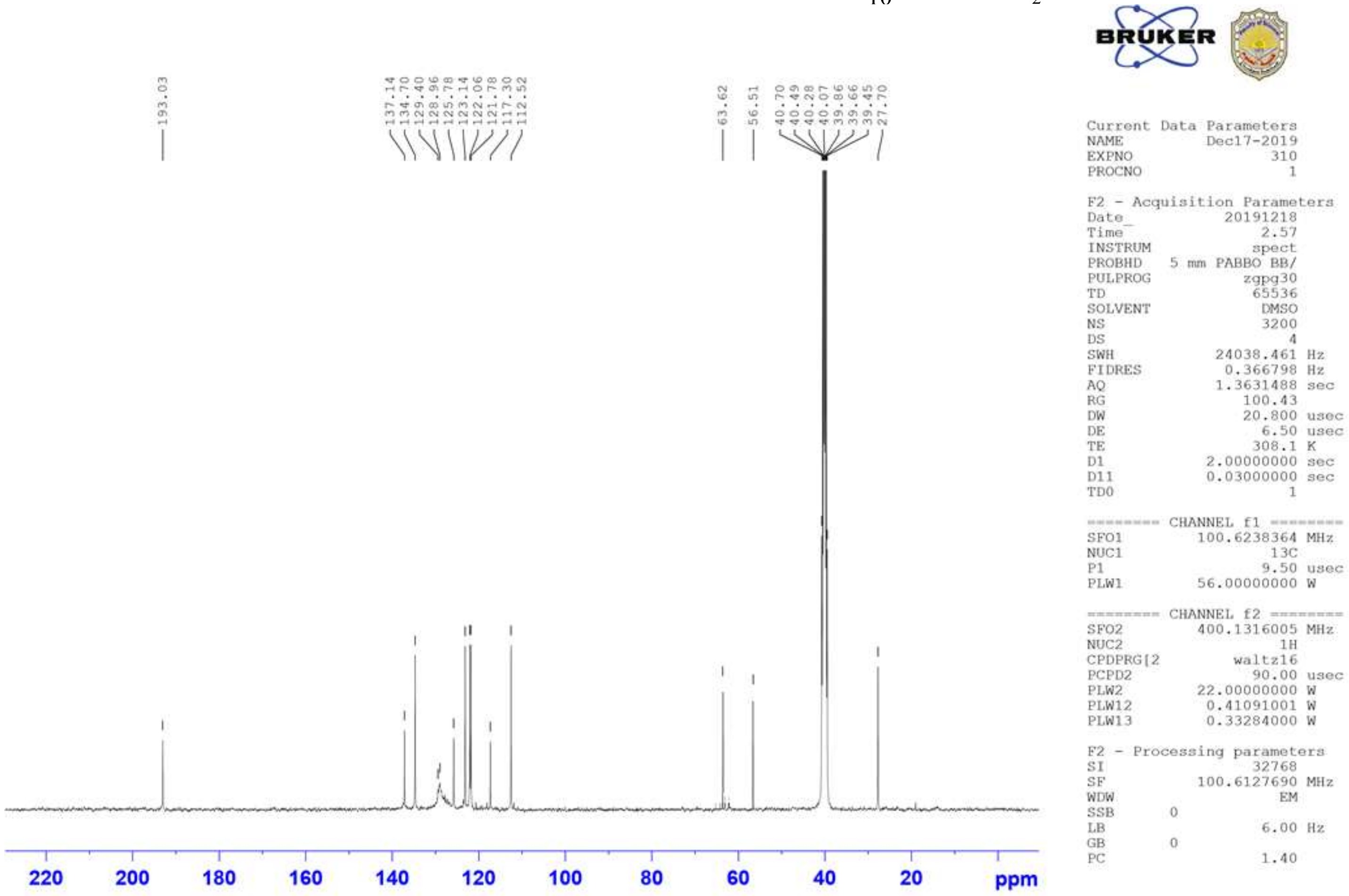

Figure38: ^{13}CNMR analysis of 10

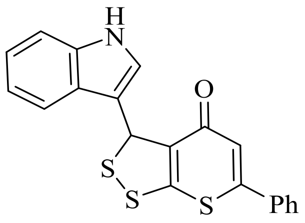

12

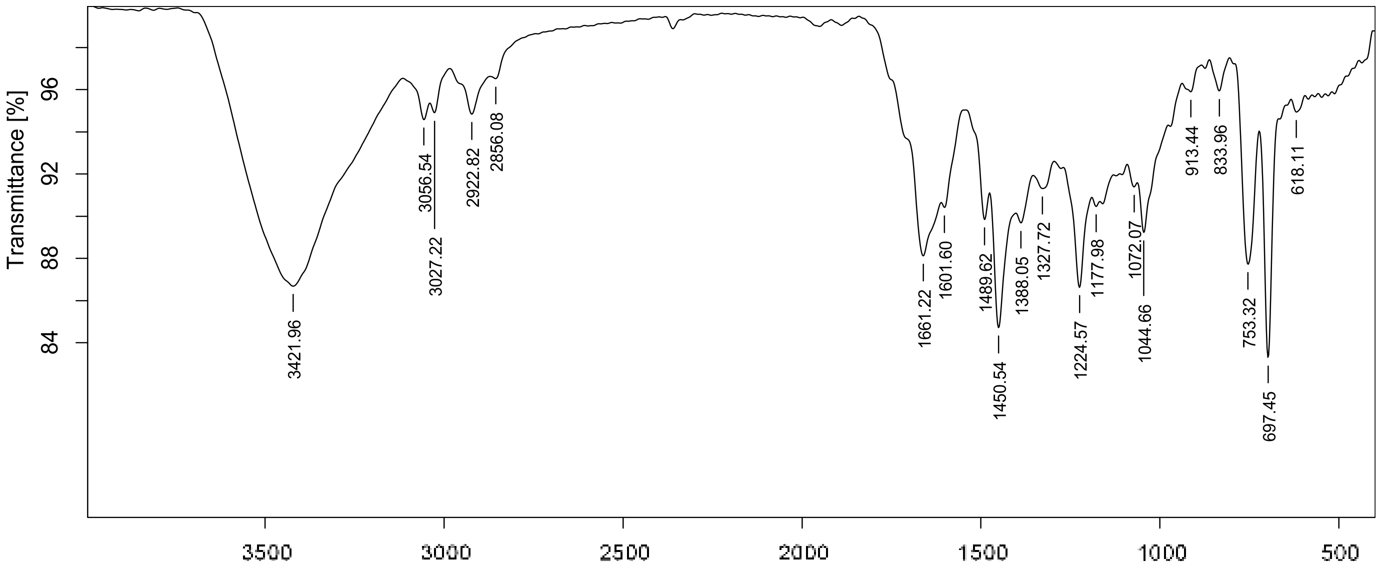

Figure 39: IR analysis of 12

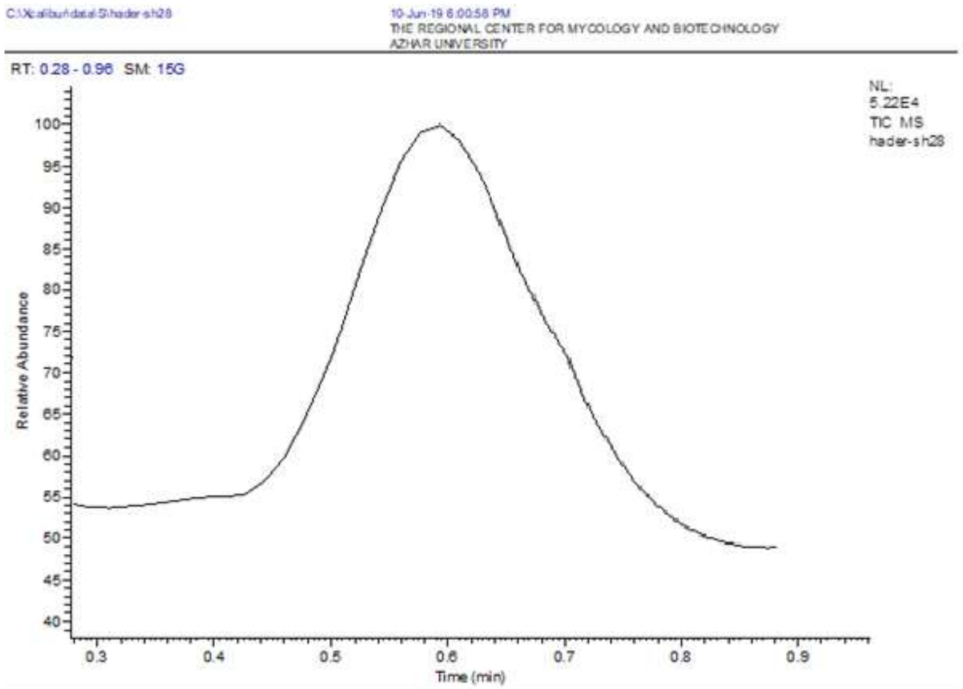

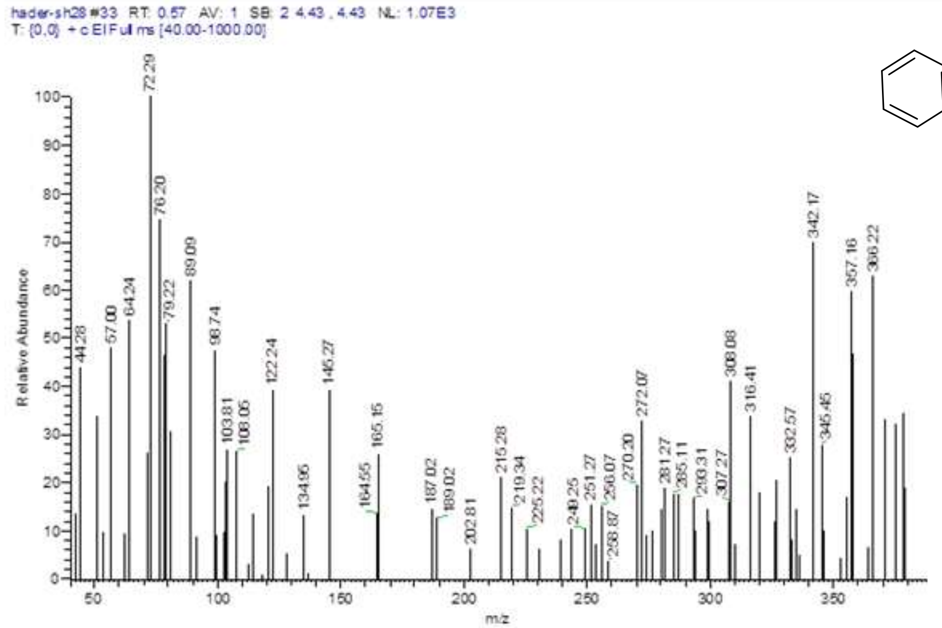

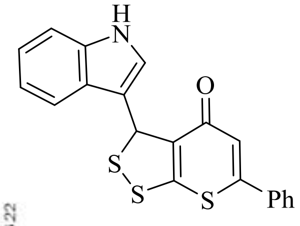

12

Figure 40 : Mass analysis of 12

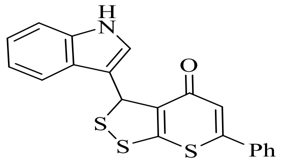

12

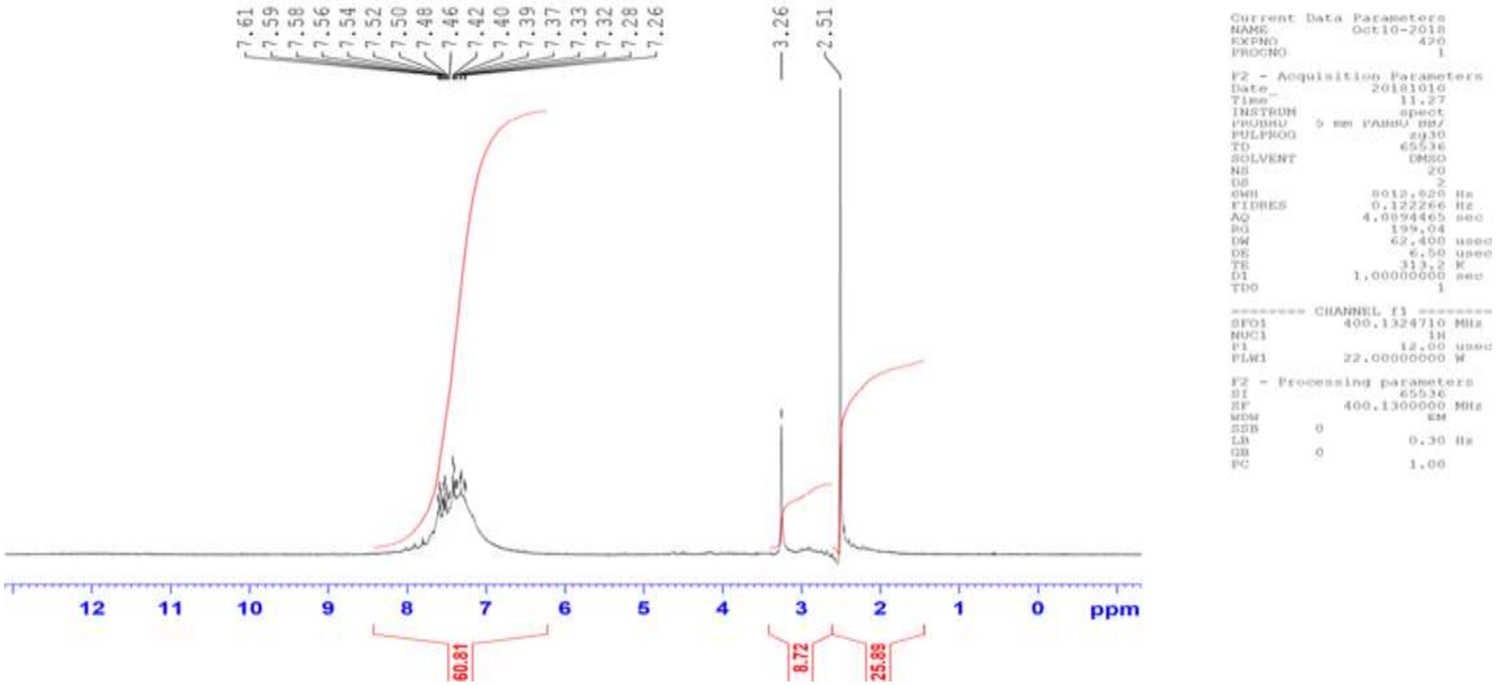

Figure 41 : ^{1}HNMR analysis of 12

**Standard drug** 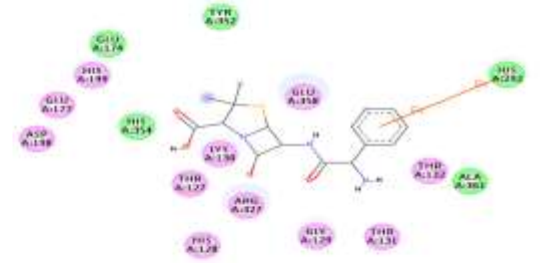	**Compound 1** 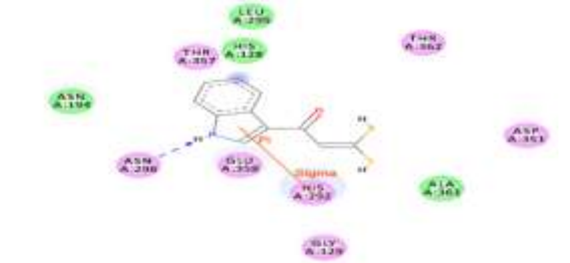
**Compound 2** 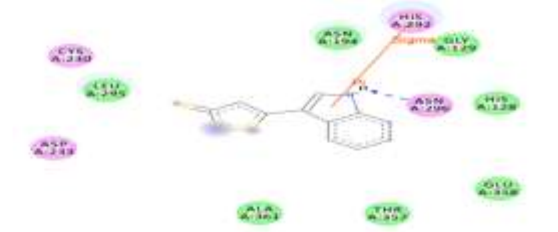	**Compound 3** 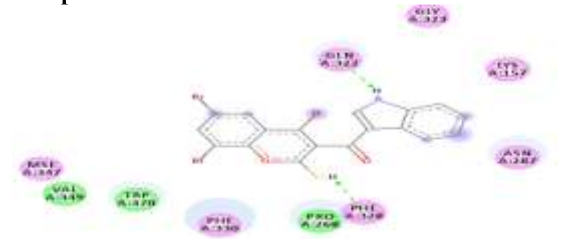
**Compound 4** 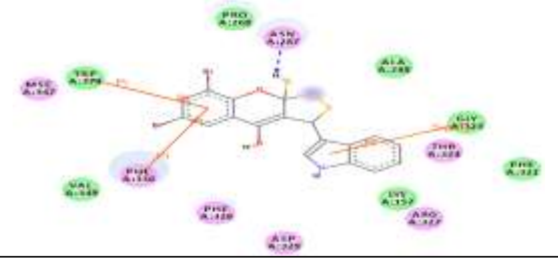	**Compound 5** 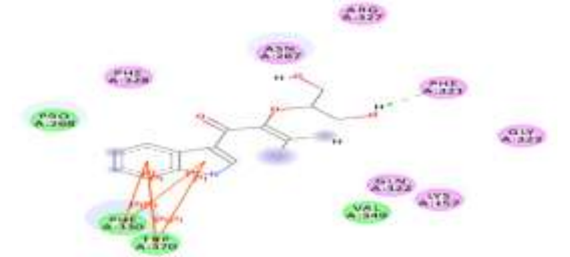
**Compound 6** 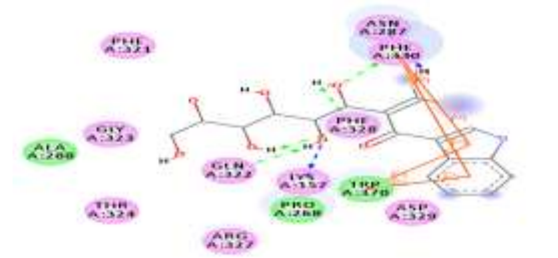	**Compound 7** 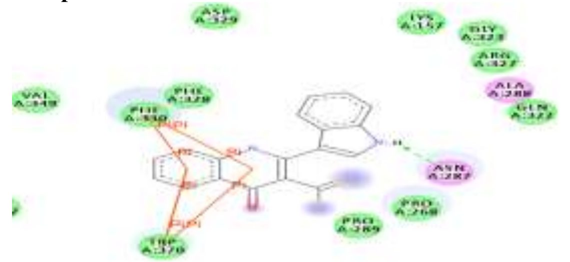
**Compound 8** 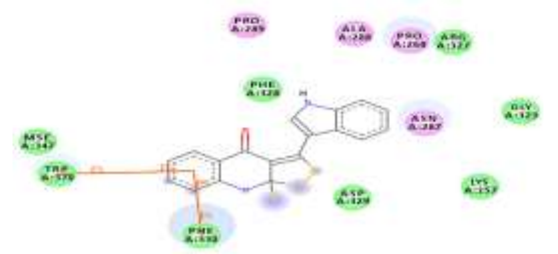	**Compound 10** 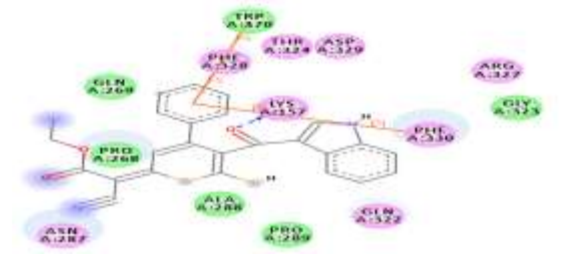
**Compound 12**	

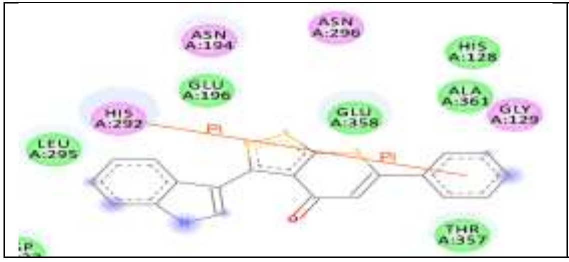

Figure S41. The 2D interaction between the other compounds with target enzyme 2F00.

Standard drug

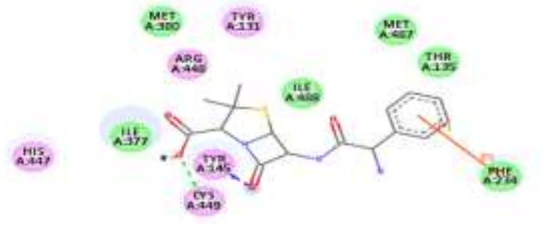

Compound 1

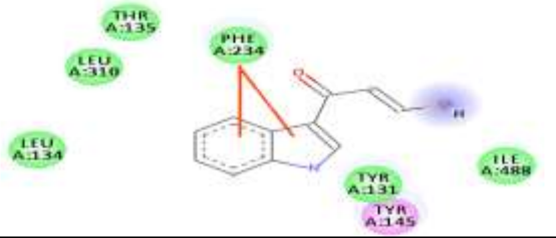

Compound 2

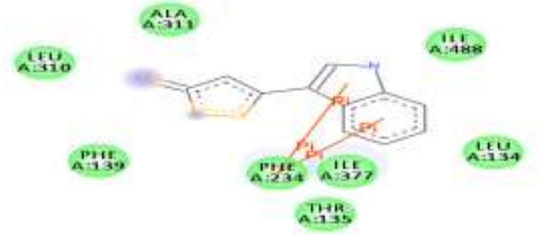

Compound 3

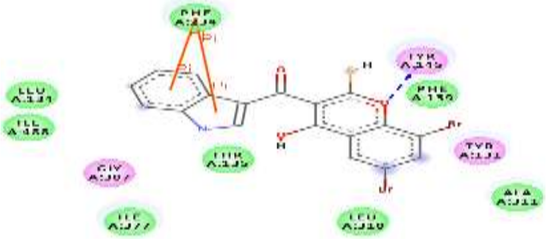

Compound 4

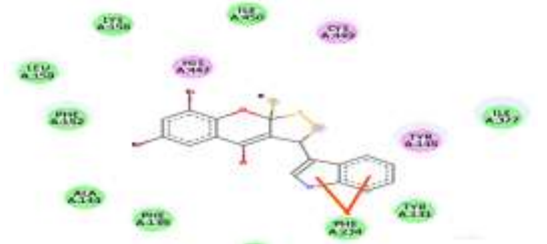

Compound 5

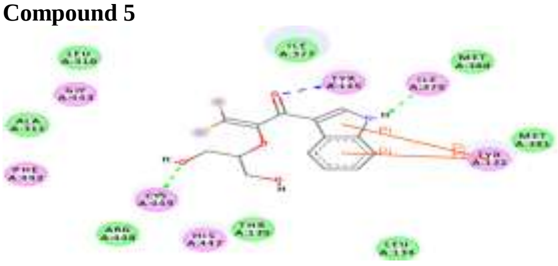

Compound 6

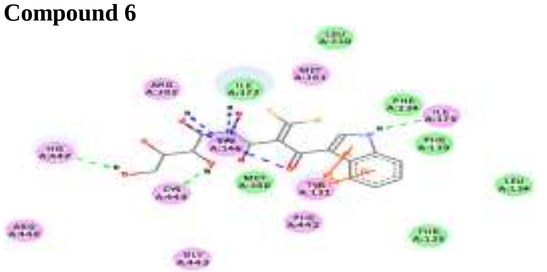

Compound 7

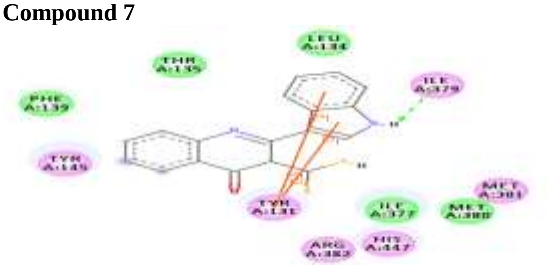

Compound 8

Compound 10

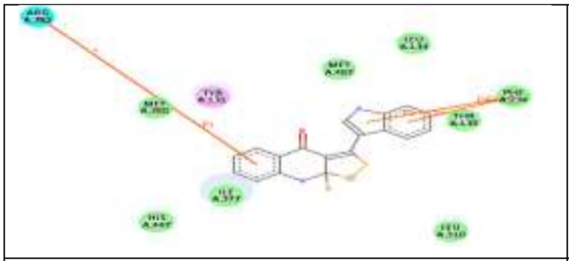

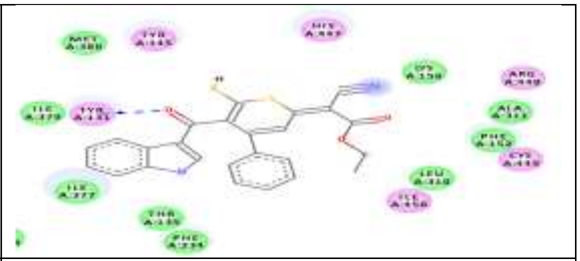

Compound 12

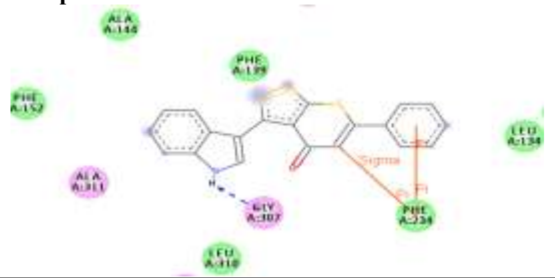

Figure S42. The 2D interaction between the other compounds with target enzyme 6uez.

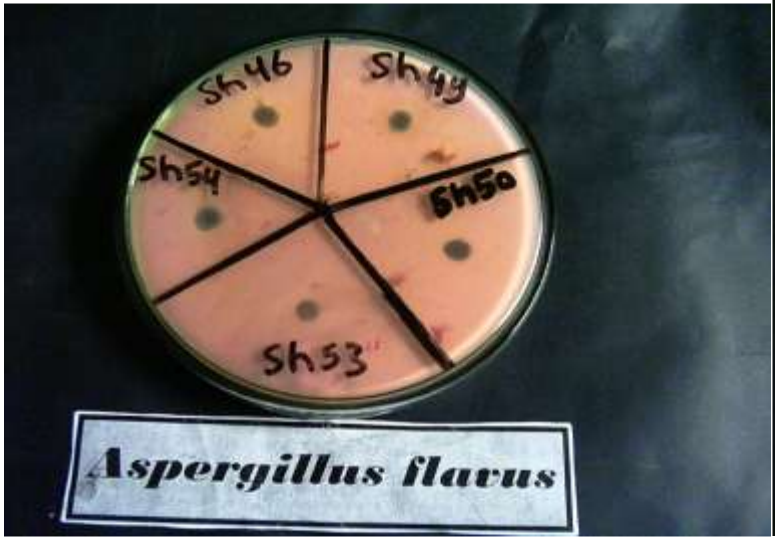
Sh46
Sh49
Sh54
Sh50
Sh53
Aspergillus flavus
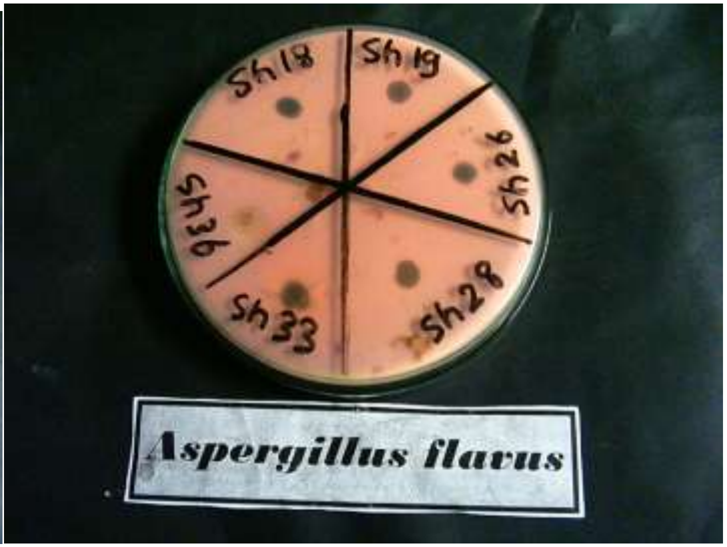
Sh18
Sh19
Sh26
Sh36
Sh33
Sh28
Aspergillus flavus
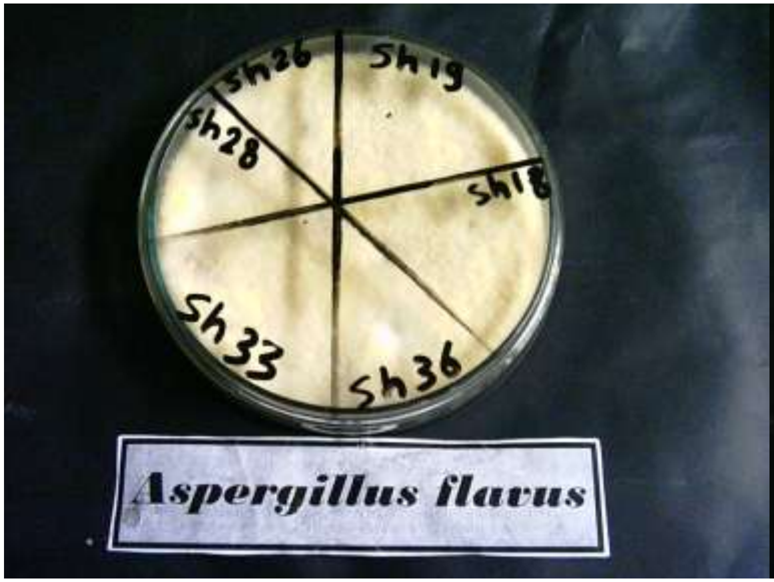
Sh26
Sh19
Sh28
Sh18
Sh33
Sh36
Aspergillus flavus
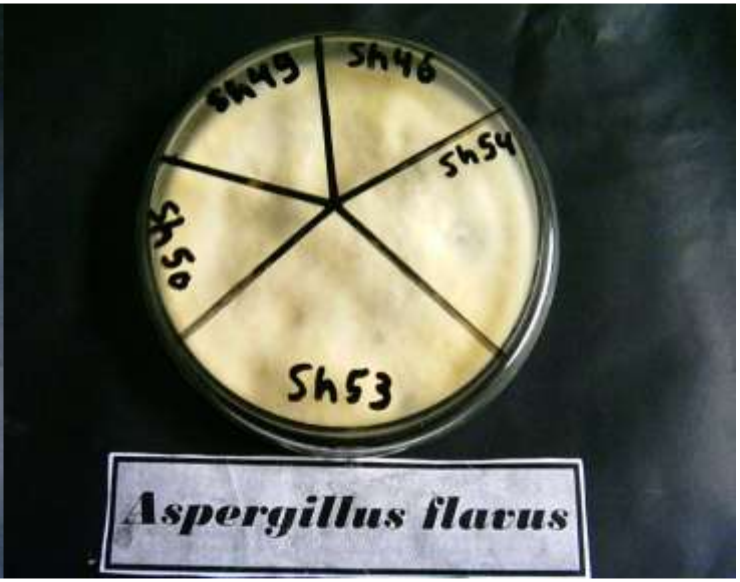
Sh49
Sh46
Sh54
Sh50
Sh53
Aspergillus flavus

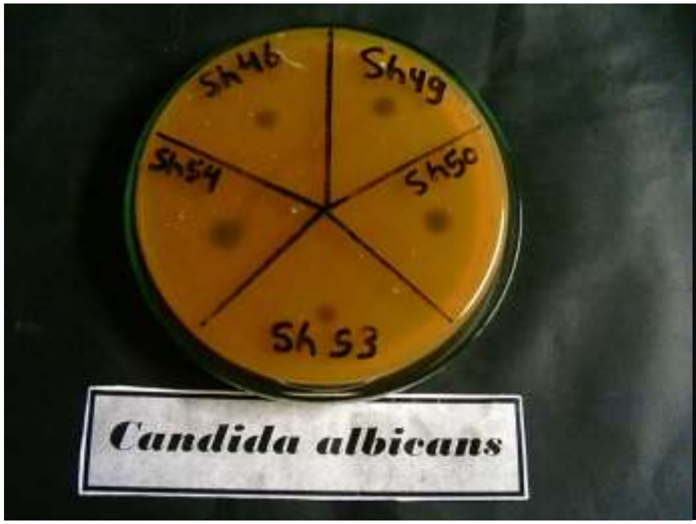
Sh46
Sh49
Sh54
Sh50
Sh53
Candida albicans

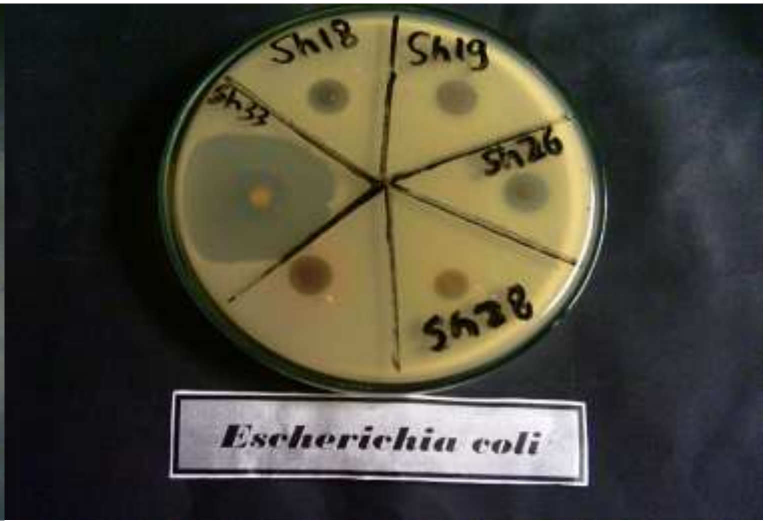
Sh18
Sh19
Sh33
Sh26
Sh28
Escherichia coli

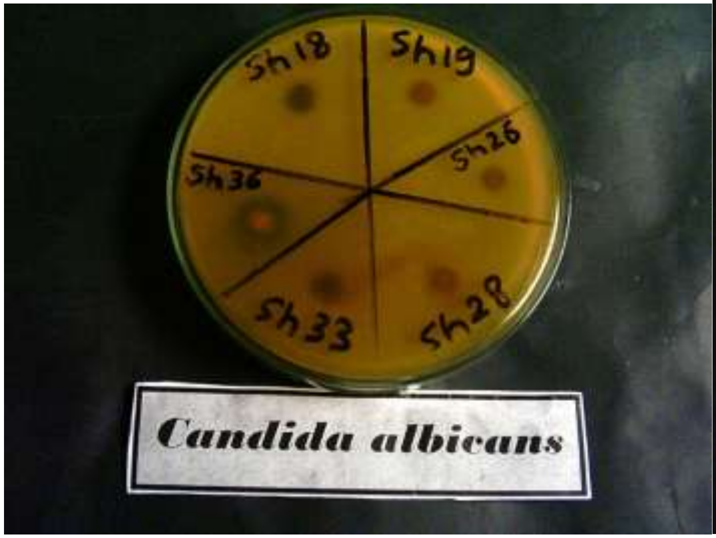
Sh18
Sh19
Sh26
Sh36
Sh33
Sh28
Candida albicans

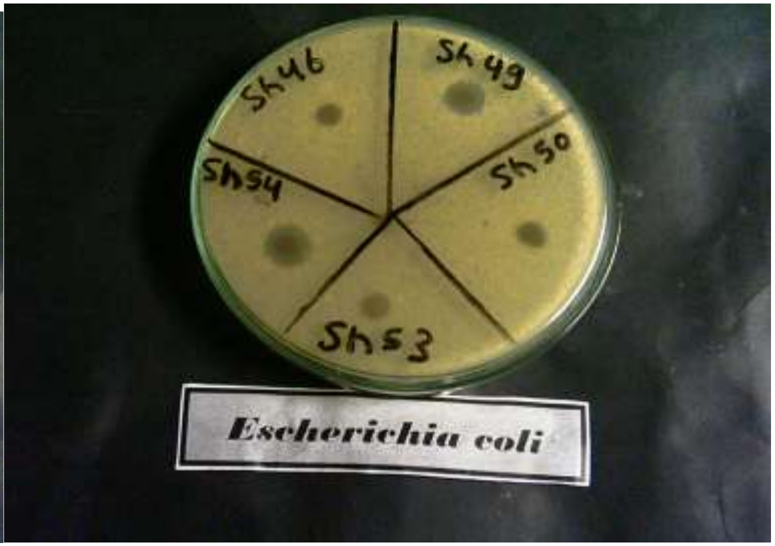
Sh46
Sh49
Sh54
Sh50
Sh53
Escherichia coli

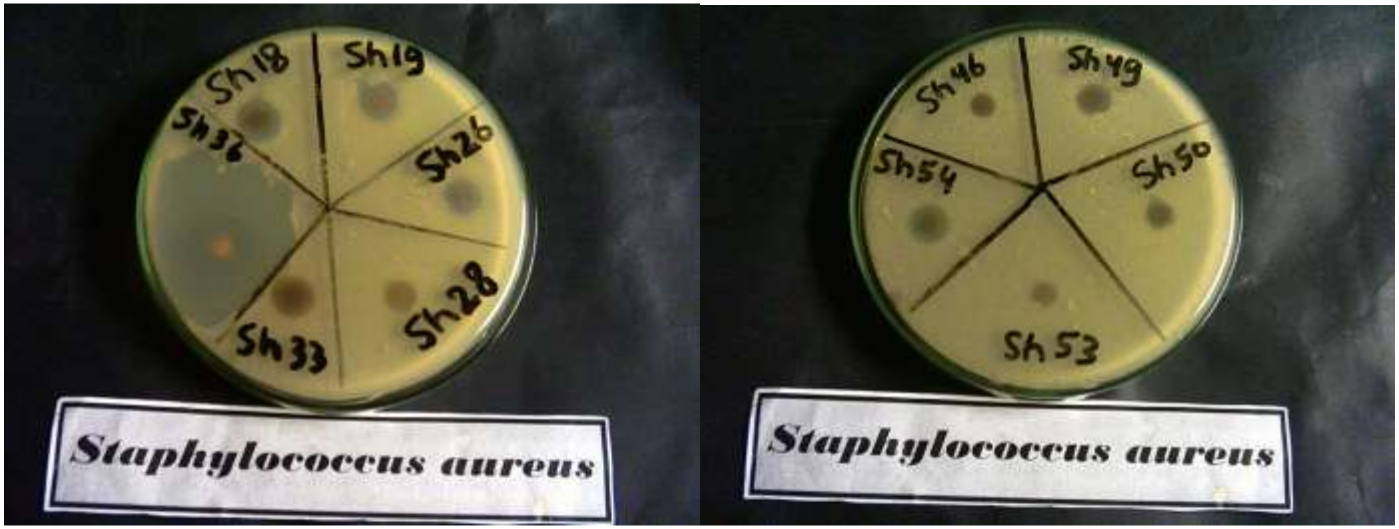

Figure S43. Antibacterial and antifungal activity of the compounds **1-12.**
