# Supplementary material for: Synthesis, Molecular Docking Analysis and in Vitro Biological Evaluation of Some New Heterocyclic Scaffolds-Based Indole Moiety as Possible Antimicrobial Agents
Source: Front Mol Biosci. 2022 Jan 17;8:775013. doi: 10.3389/fmolb.2021.775013 (PMC8801890; doi:10.3389/fmolb.2021.775013)
Supplement: Supplementary file 2 [file DataSheet1.docx]

**Supplementary data (Figure S1-** **Figure S43)**

**Synthesis, Molecular Docking Analysis and In vitro Biological Evaluation of Some New Heterocyclic Scaffolds-based Indole Moiety as Possible Antimicrobial Agents**

Entesar A. Hassan^1^, Ihsan A. Shehadi^2^, Awatef M. Elmaghraby^1^, Hadir M. Mostafa^1^, Salem E. Zayed^1^, Aboubakr H. Abdelmonsef ^1,*^

^1^ Chemistry Department, Faculty of Science, South Valley University, Qena 83523, Egypt

^2^ Chemistry Department, Faculty of Science, University of Sharjah, Sharjah 27272, UAE

*Correspondence; aboubakr.ahmed@sci.svu.edu.eg

| **Standard drug**  **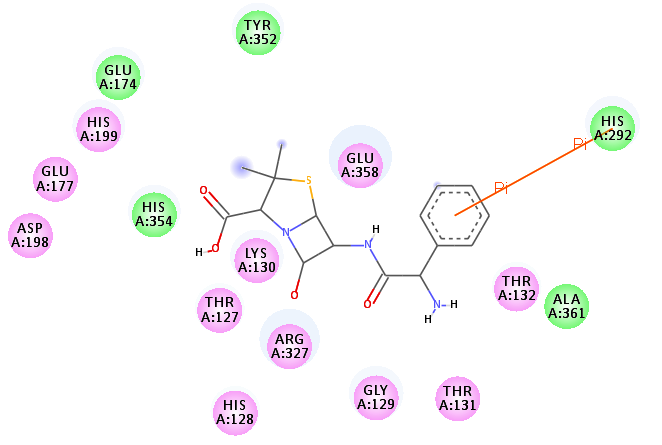** | **Compound 1**  **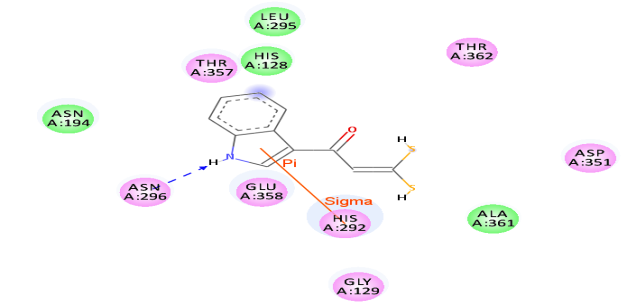** |
| --- | --- |
| **Compound 2**  **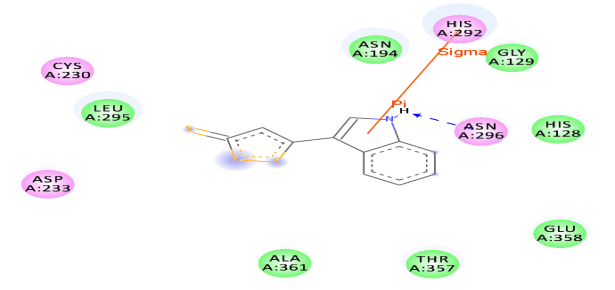** | **Compound 3**  **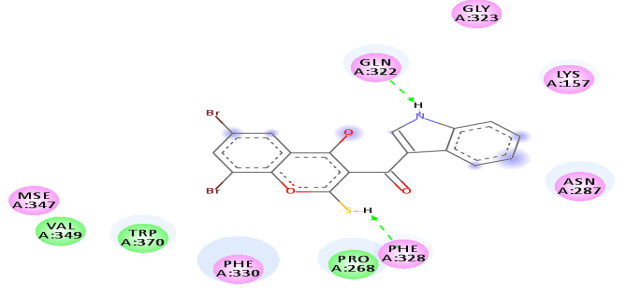** |
| **Compound 4**  **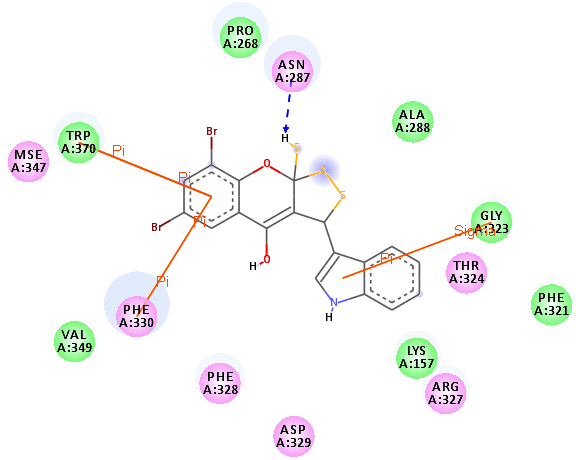** | **Compound 5**  **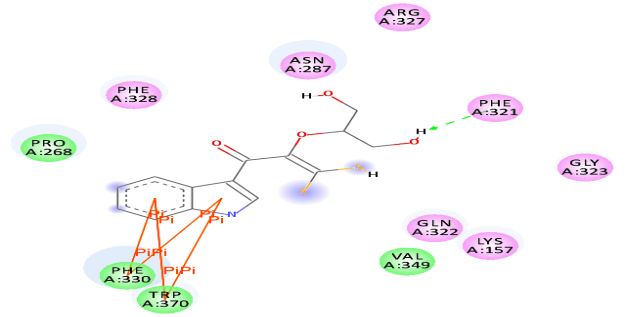** |
| **Compound 6**  **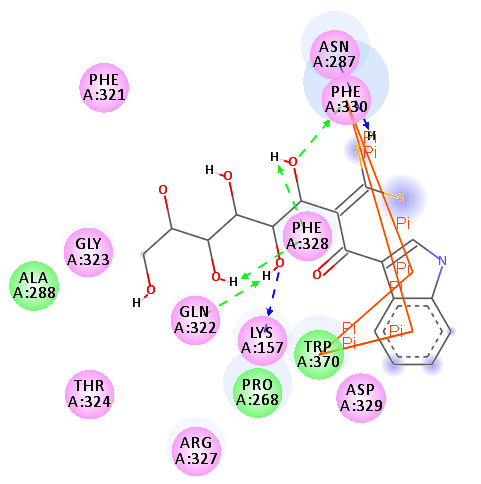** | **Compound 7**  **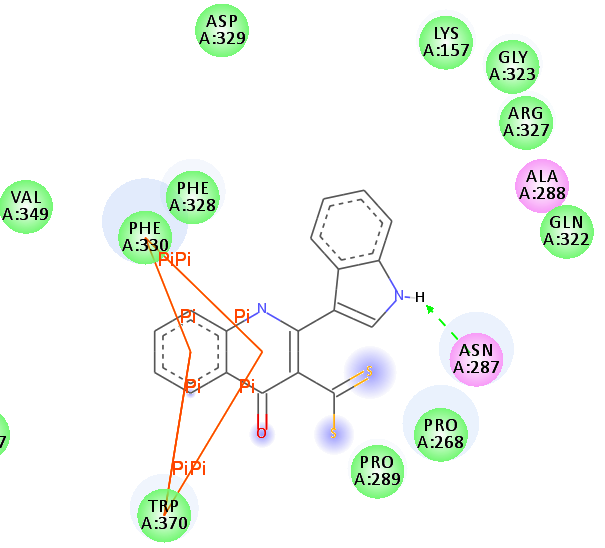** |
| **Compound 8**  **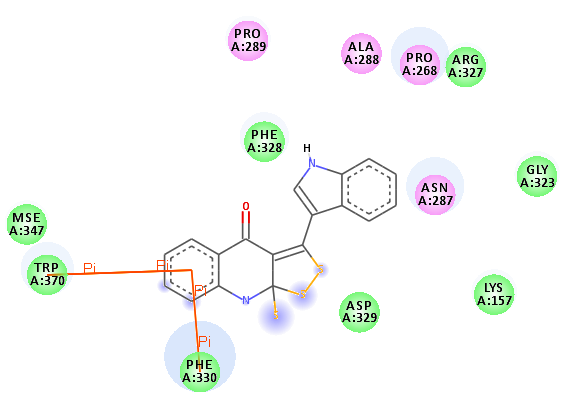** | **Compound 10**  **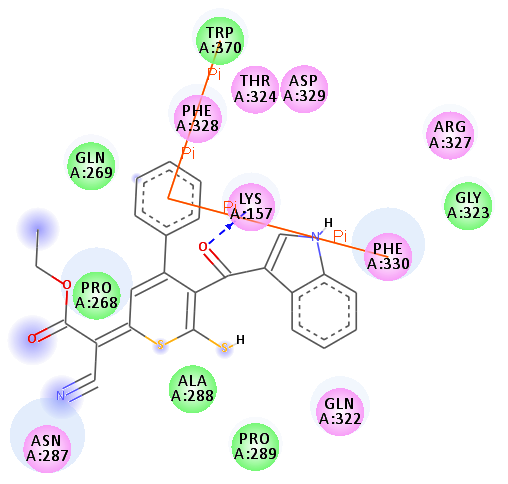** |
| **Compound 12**  **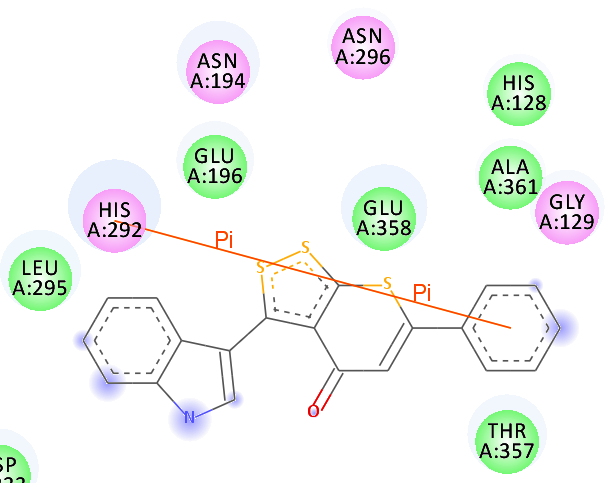** |  |

**Figure S41.** The 2D interaction between the other compounds with target enzyme 2F00.

| **Standard drug**  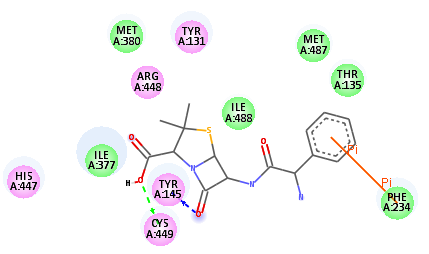 | **Compound 1**  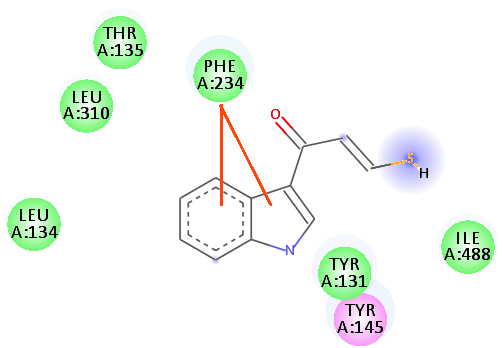 |
| --- | --- |
| **Compound 2**  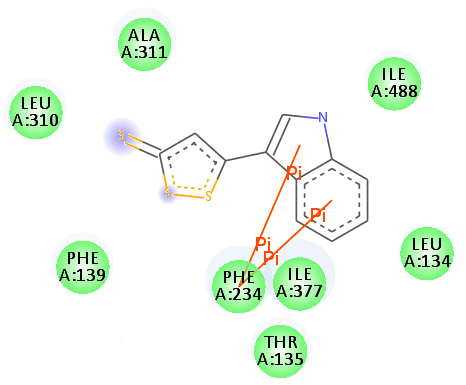 | **Compound 3**  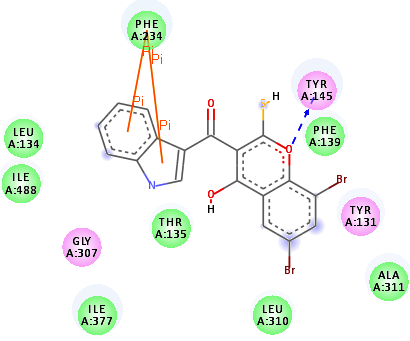 |
| **Compound 4**  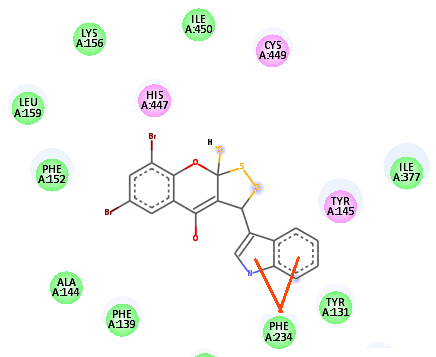 | **Compound 5**  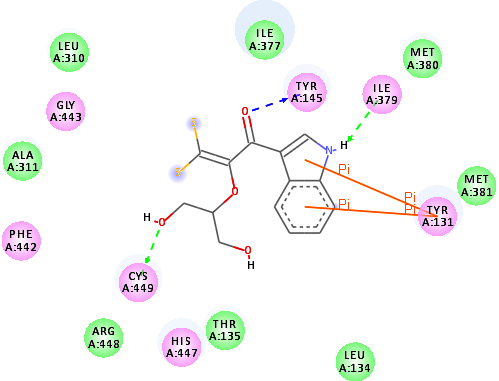 |
| **Compound 6**  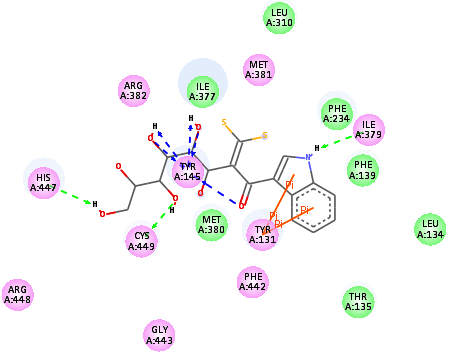 | **Compound 7**  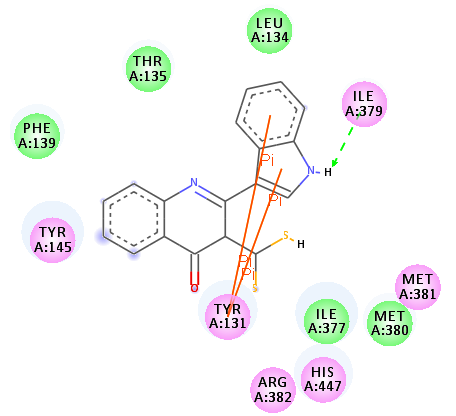 |
| **Compound 8**  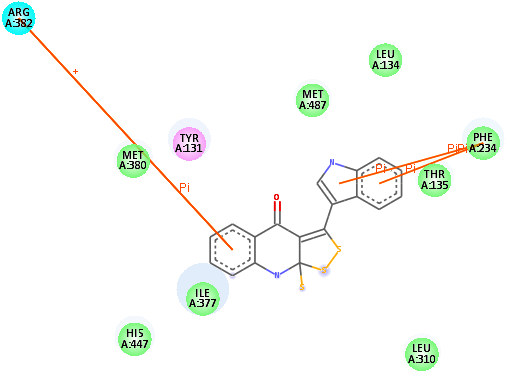 | **Compound 10**  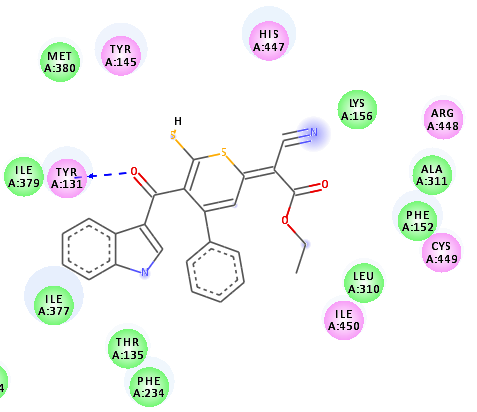 |
| **Compound 12**  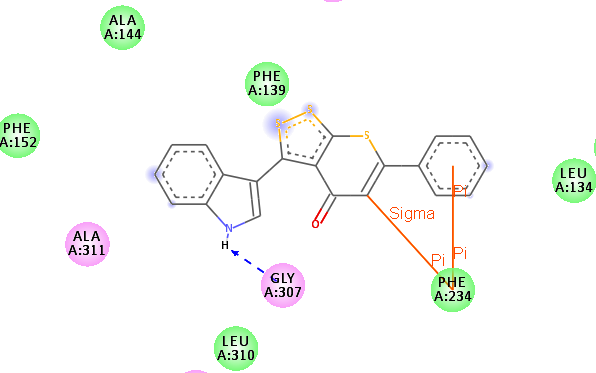 |  |

**Figure S42.** The 2D interaction between the other compounds with target enzyme 6uez.


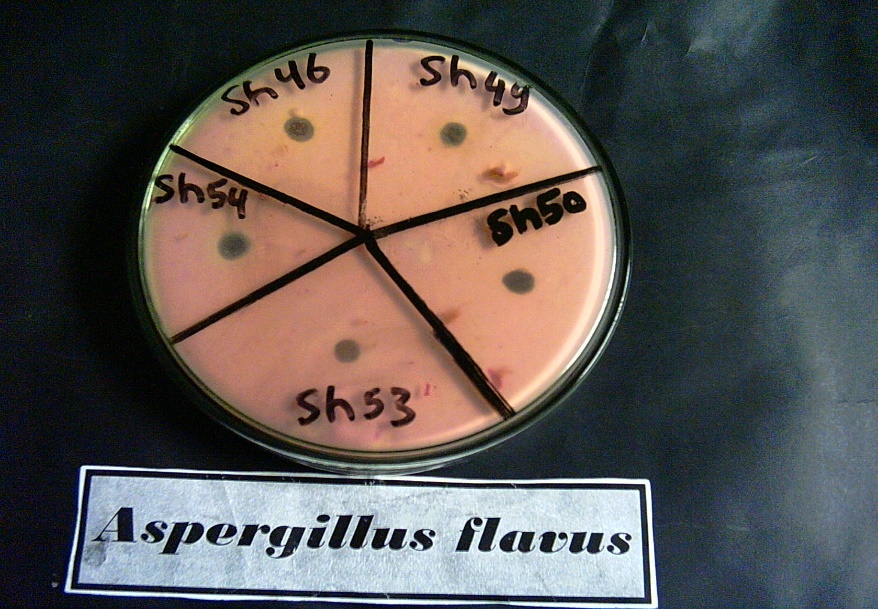

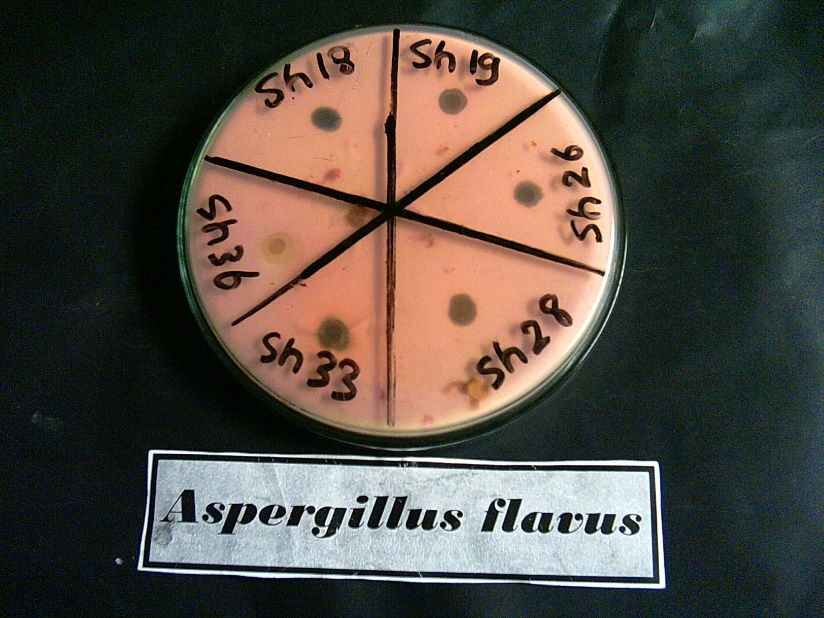


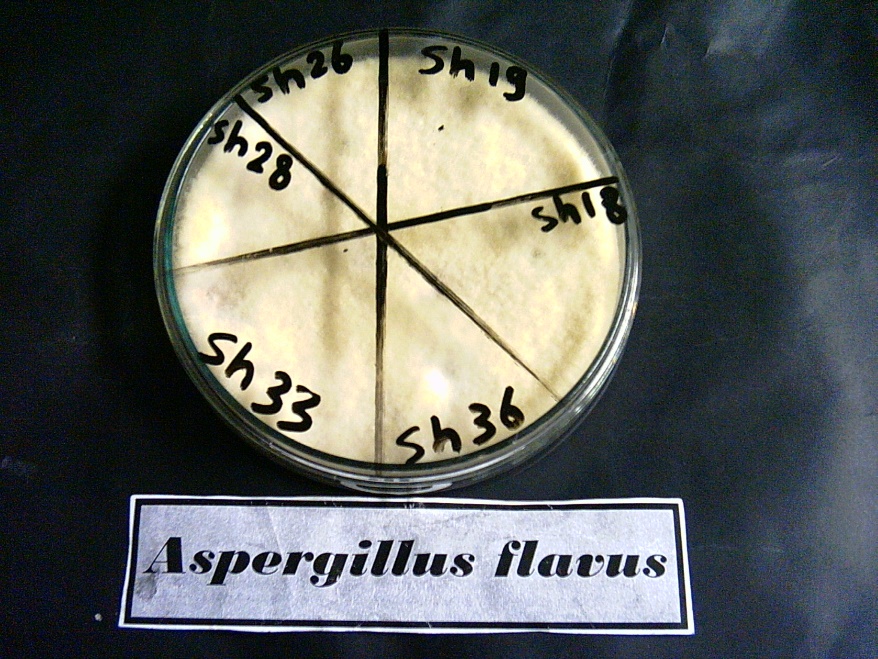

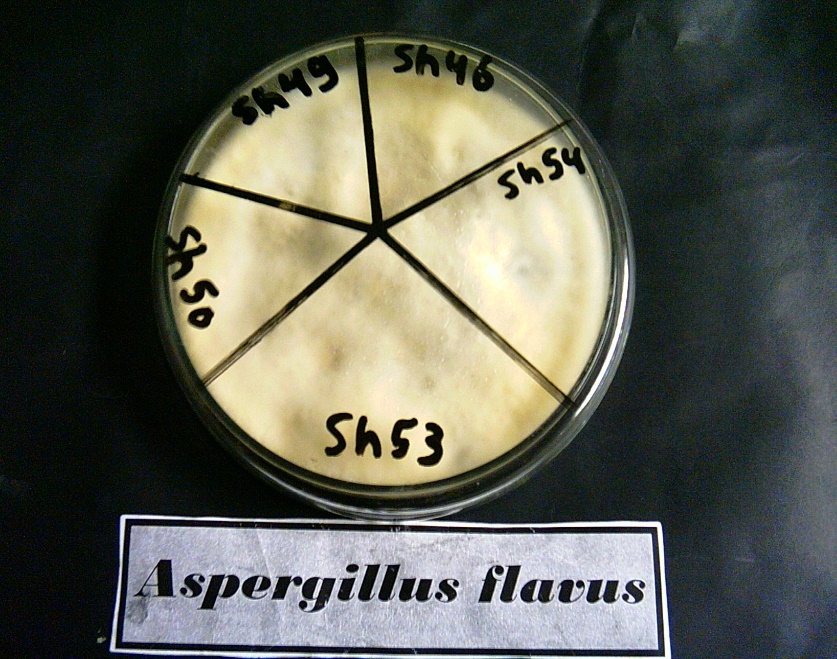


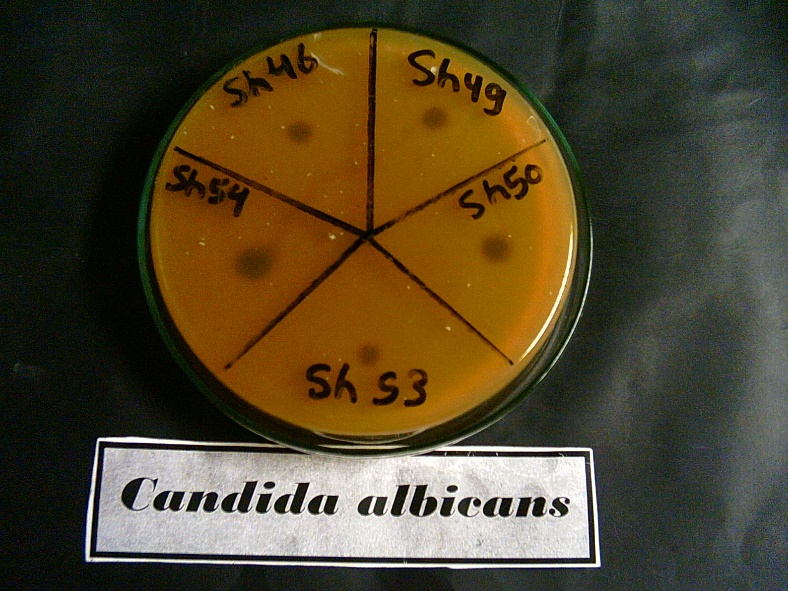

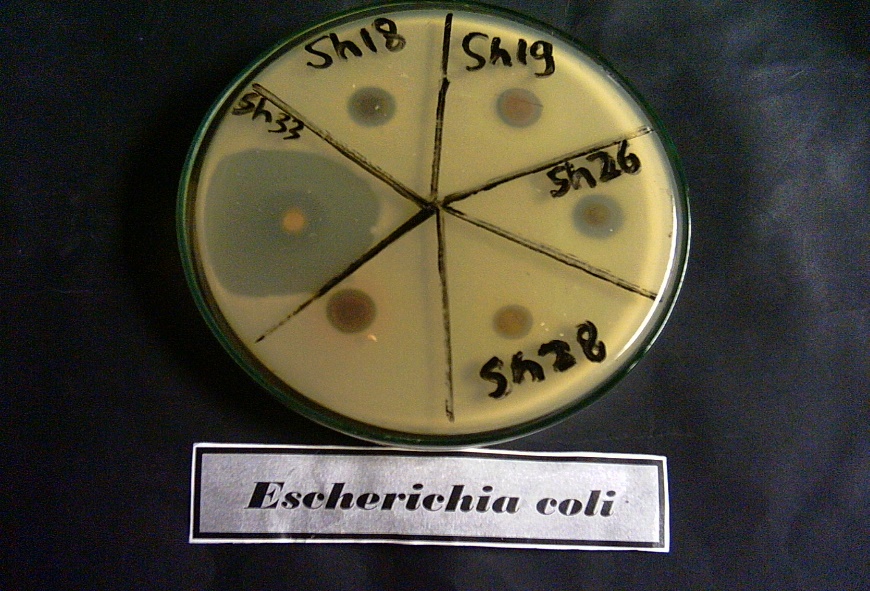


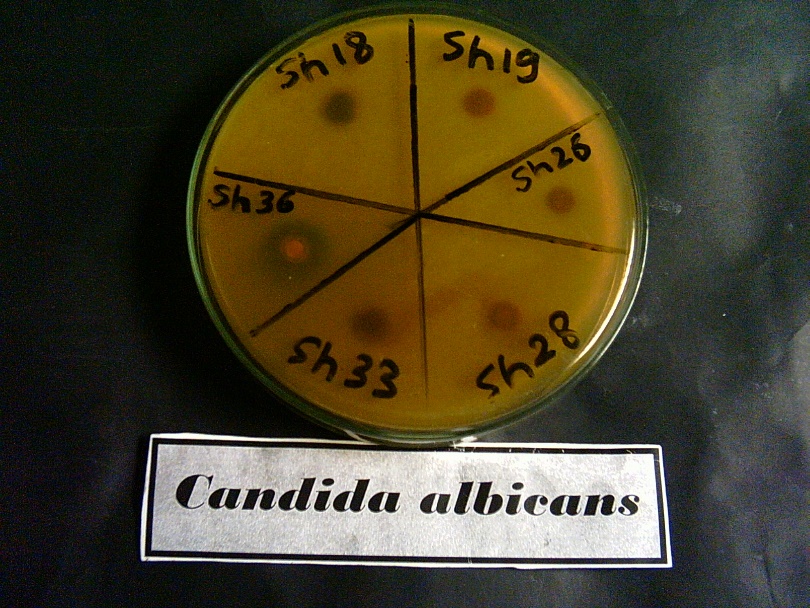

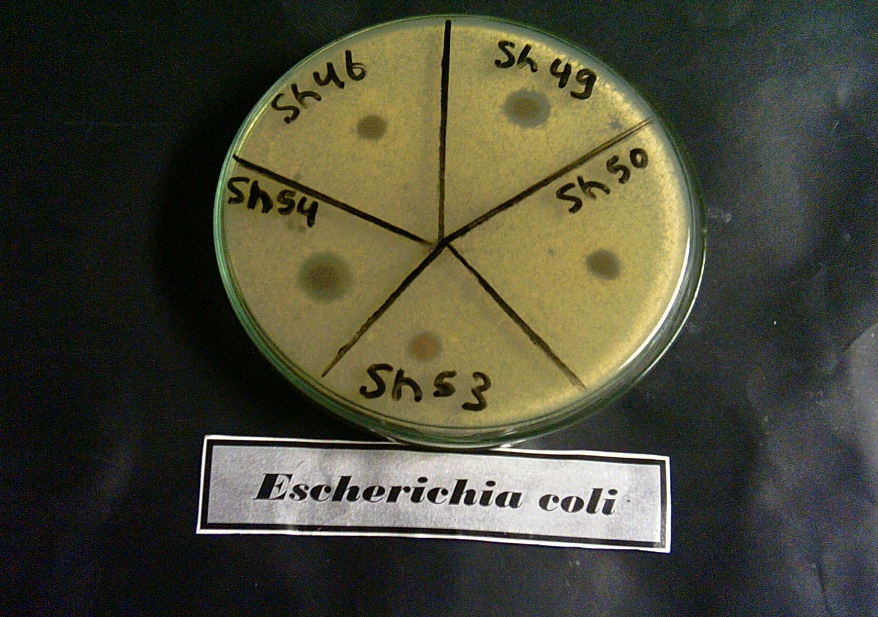


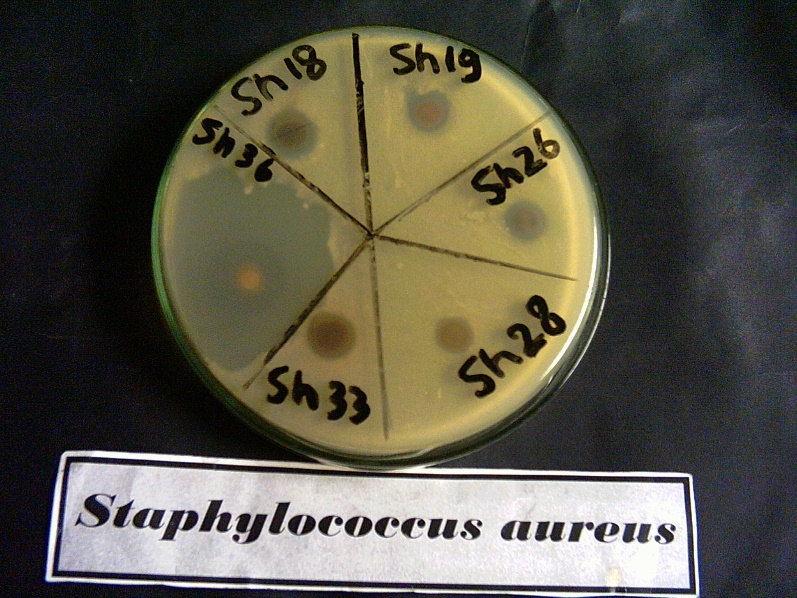

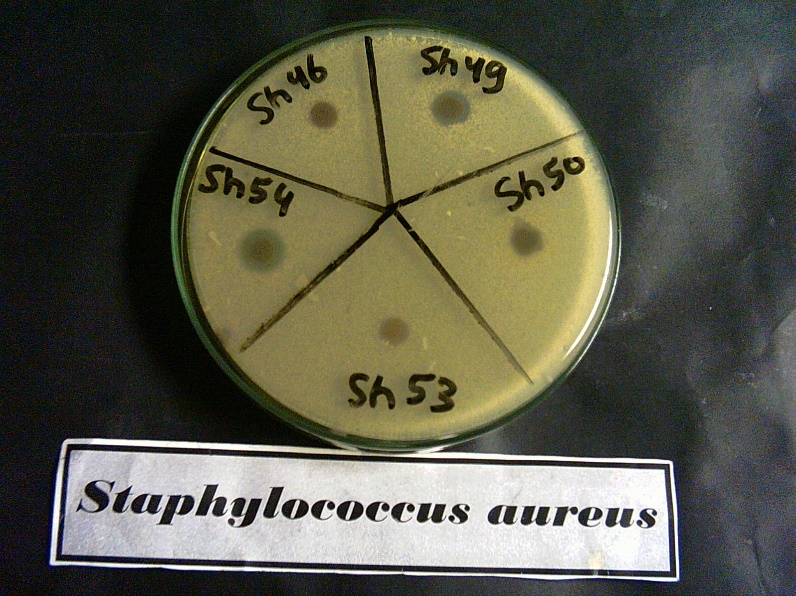


**Figure S43.** Antibacterial and antifungal activity of the compounds **1-12.**
